# The effectiveness of Chuna manual therapy based on radiographic malposition diagnosis in patients with non-acute low back pain: A study protocol for a randomized, assessor-blind, parallel-group, controlled trial

**DOI:** 10.1371/journal.pone.0347321

**Published:** 2026-05-11

**Authors:** JungHo Jo, Yeong Ha Jeong, Tae-Yong Park, Young Cheol Na, Jun-Su Jang, Minho Choi, Junghan Lee, Kang-san Kim, Yoonhee Kim, Seungkwan Choi, Wonbae Ha, Jin-Hyun Lee

**Affiliations:** 1 Department of Internal Medicine, College of Korean Medicine, Wonkwang University, Iksan-si, Jeonbuk-do, Republic of Korea; 2 Department of Neurosurgery, Catholic Kwandong University International St. Mary’s Hospital, Catholic Kwandong University College of Medicine, Incheon, Republic of Korea; 3 Institute for Integrative Medicine, Catholic Kwandong University International St. Mary’s Hospital, Incheon, Republic of Korea; 4 Digital Health Research Division, Korea Institute of Oriental Medicine, Daejeon, Republic of Korea; 5 Department of Korean Medicine Rehabilitation, College of Korean Medicine, Wonkwang University, Iksan-si, Jeonbuk-do, Republic of Korea; Lady Reading Hospital, PAKISTAN

## Abstract

**Background:**

Chuna manual therapy is a Korean therapeutic modality that is widely used for treating low back pain. First, spinal alignment is radiographically assessed, followed by treatment delivery as per the assessment. There is emerging evidence supporting radiograph-based diagnostic methods in Chuna manual therapy. However, there remains limited high-quality clinical evidence regarding the effectiveness of treatment tailored to specific diagnostic types. Accordingly, this study aims to evaluate the effectiveness and safety of Chuna manual therapy in patients with non-acute low back pain radiographically diagnosed with spinal malposition.

**Methods:**

This two-arm, parallel-group, assessor-blind, randomized controlled trial will include 46 participants with non-acute low back pain lasting ≥3 weeks and an average Numeric Rating Scale (NRS) score ≥4 from two hospitals. Participants will be randomly assigned in a 1:1 ratio to the usual care plus Chuna manual therapy intervention group (n = 23) or the usual care intervention group (n = 23). Participants in the intervention group will receive individualized Chuna manual therapy based on the radiographic diagnosis twice per week for 4 weeks. The primary outcome will be the change in the NRS score for low back pain at Week 4 and Week 12 compared with baseline. The secondary outcomes will include changes in the Oswestry Disability Index, Roland Morris Disability Questionnaire, EuroQol-5 Dimension, radiographic alignment indices, Range of Motion assessment, and safety evaluation.

**Discussion:**

This will be the first randomized controlled trial to investigate the effectiveness of Chuna manual therapy based on the radiographic diagnosis of spinal malposition in patients with non-acute low back pain. The findings may strengthen the evidence base for diagnostic and therapeutic processes in Chuna manual therapy.

**Trial registration:**

This study is registered with the Clinical Research Information Service of the Korea Disease Control and Prevention Agency. Registration number: KCT0011042.

## Introduction

### Background and rationale

Low back pain (LBP) is among the most prevalent musculoskeletal disorders; further, it is a major cause of activity limitation and disability, which imposes a substantial socioeconomic burden [1]. Most acute episodes of LBP subsequently improve; however, a considerable proportion of cases progress to non-acute or chronic LBP [2], which represents a major challenge in modern medicine. Conservative treatments include pharmacologic, physical, and injection therapies; however, they often have limited long-term effectiveness and may involve adverse effects. Accordingly, there is increasing demand for more effective and safer non-pharmacologic treatments [3].

There has been increasing interest in manual therapy as a crucial non-pharmacologic treatment for patients with non-acute and chronic LBP. Manual therapy includes spinal manipulation, joint mobilization, and soft tissue techniques; further, it has demonstrated beneficial effects in patients with chronic LBP [4,5]. Specifically, manual therapy has been shown to restore abnormal joint motion, relieve tension in surrounding muscles, and induce neurophysiological changes contributing to therapeutic effects [6,7].

Chuna manual therapy is a traditional Korean modality in which doctors use their hands, body, or auxiliary tools to push or pull the patient’s spine, joints, and muscles for correction of structural and functional problems. It has demonstrated clinical effectiveness and safety; further it is covered under the Korean national health insurance system for spinal disorders [8,9]. Specifically, Chuna manual therapy acts along meridian pathways to correct structural malpositions and exert therapeutic effects across various diseases, especially through pain reduction and functional improvement in musculoskeletal disorders [8,10].

In Chuna manual therapy, the diagnosis is based on both symptoms and the underlying structural cause of pain. Korean medicine doctors diagnose spinal malposition using palpation and radiographic imaging, followed by application of the appropriate Chuna manual therapy. In Chuna medicine, radiograph-based diagnosis has higher inter-rater and intra-rater agreement and diagnostic efficiency than palpation alone [11]. Additionally, quantitative radiographic angle measurements have been applied to determine malposition targets for Chuna manual therapy [12]. However, there remains limited clinical evidence regarding whether radiograph-based malposition diagnosis contributes to treatment effectiveness and functional improvement. Accordingly, this study will aim to evaluate the superiority and safety of a treatment intervention based on radiograph-based Chuna diagnosis.

### Objectives

The primary study objective is to evaluate the clinical effectiveness and safety of Chuna manual therapy guided by radiograph-based malposition diagnosis. We hypothesize that, among patients with non-acute LBP who demonstrate spinal malposition on plain radiographs, the addition of Chuna manual therapy to usual care (physical therapy, exercise, and lifestyle guidance) will yield superior pain reduction and functional improvement compared with usual care alone.

## Methods/design

### Trial design

**Fig 1:**
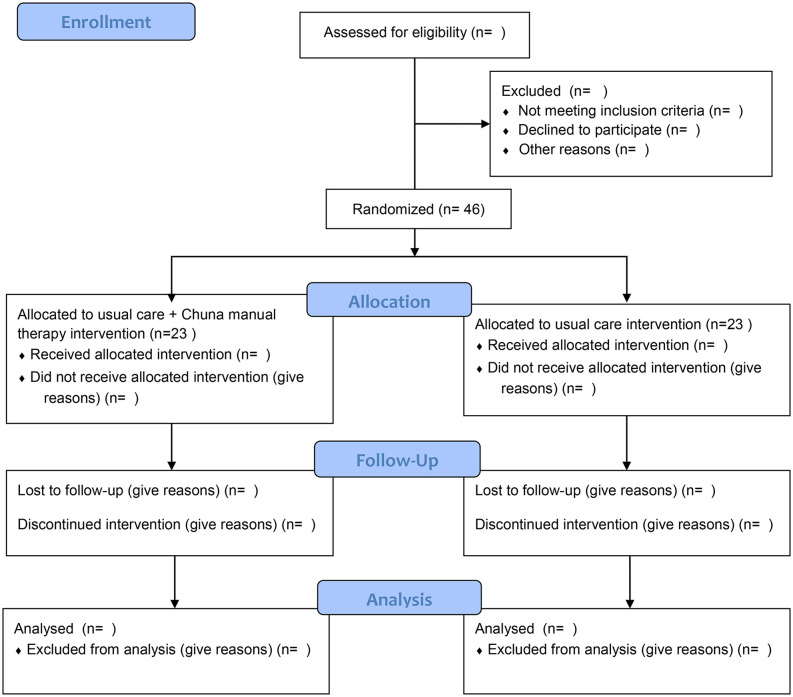
Flow chart of the study CONSORT Flow Diagram.

This will be a multicenter, two-arm, parallel-group, assessor-blind, randomized controlled trial. Patients with non-acute LBP for ≥3 weeks will undergo standardized L-spine standing anteroposterior and lateral radiography to determine the presence or absence of malposition based on the Chuna manual therapy diagnostic criteria [12,13] (S1 Checklist). Inter-rater reliability of the radiograph-based malposition diagnosis has been previously evaluated using the same diagnostic criteria and trained assessors [11,12]. Because the assessors involved in the prior reliability study will continue to participate in the present trial, an additional formal inter-rater reliability assessment has not been planned. Participants who have malposition and meet the relevant criteria will be randomly assigned in a 1:1 ratio to the usual care plus Chuna manual therapy intervention group (Group I) or the usual care intervention group (Group II). The study period will comprise screening, a 4-week treatment period, and an 8-week follow-up period. This protocol has been developed in accordance with the SPIRIT guidelines, and the schedule of enrolment, interventions, and assessments is summarized in the SPIRIT schedule (Table 1). The anticipated flow of participants through screening, randomization, allocation, follow-up, and analysis is illustrated in the CONSORT flow diagram (Fig 1).

**Table 1:** SPIRIT schedule.

Period		Screening	Baseline^a)^	Treatment								Post	Early Termination
												Treatment	
Visit		Visit 1	Visit 2	Visit 3	Visit 4	Visit 5	Visit 6	Visit 7	Visit 8	Visit 9	Visit 10	Visit 11	Early Withdrawal
Day		−7 ~ –0	0	1–3	4–6	7–10	11–14	15–18	18–21	22 ~ 24	25–28	81-84	
Written informed consent		√											
Demographics		√											
Medical history and medication history		√											
Screening tests^b)^		√											
Confirmation of inclusion and exclusion criteria		√											
Randomization (assignment of registration number)			√										
Physical examination and vital signs		√	√	√	√	√	√	√	√	√	√	√	√
Assessment of concomitant medication^c)^				√	√	√	√	√	√	√	√	√	√
Treatment	Usual care^d)^ + Chuna manual therapy intervention^e)^			√	√	√	√	√	√		√		
	Usual care intervention			√	√	√	√	√	√		√		
Primary outcome assessment^f)^			√								√	√	√
Secondary outcome assessment^g)^			√								√	√	√
Exploratory effectiveness assessment^h)^												√	√
Adverse event monitoring				√	√	√	√	√	√	√	√	√	√
Safety assessment				√	√	√	√	√	√	√	√	√	√

a)Screening and baseline tests may be conducted on the same day (Visit 1 and Visit 2 can be merged as Visit 1). If screening and baseline are performed separately, 1–2 weeks are generally required between the screening test and start of active treatment.

b)Screening tests include demographics, medical history, physical examination, vital signs, radiographic-guided malposition diagnosis based on Chuna medicine criteria, and confirmation of inclusion and exclusion criteria.

c)>At each treatment or assessment visit, we will monitor for concomitant medication use and adverse events.

d)Usual care comprises ICT, exercise therapy, and lifestyle education.

e)Chuna manual therapy will be applied according to standardized procedures based on the location and type of malposition in accordance with the Chuna medicine criteria.

f)NRS assessment for LBP.

g)NRS score for leg pain, ODI, Korean version of the RMDQ, Quality of Life evaluation using the EQ-5D, physical examination (thoracolumbar range of motion), and radiographic evaluation (radiographic evaluation at Visit 2 will not be separately performed but will be replaced by the screening radiograph obtained at Visit 1).

h)Within 8 weeks after treatment completion, we will assess the proportion of participants who undergo additional invasive medical procedures related to LBP [injection therapy (including nerve block procedures), radiofrequency ablation], use of strong opioid analgesics, and lumbar surgery. Exploratory assessments will not be performed for early termination cases. For dropout cases, the exploratory assessment will be performed at 8 weeks after dropout.

Note: A visit window of ±3 days may be allowed due to weekends or public holidays.

*Protocol version and identifier:* Version 1.1, dated June 04, 2025; Protocol ID: Chuna-RCT-V1.0.

### Ethical considerations

• This study will be conducted in accordance with the ethical principles of the Declaration of Helsinki and Korean Good Clinical Practice. The protocol has been reviewed and approved by the Institutional Review Board of each participating institution: Catholic Kwandong University International St. Mary’s Hospital (IRB No. IS25EIMI0027; approval date: 12 May 2025) and Wonkwang University Korean Medicine Hospital (IRB No. 2025−06; approval date: 1 October 2025). All participants will be fully informed about the study purpose, procedures, potential risks and benefits, and written informed consent will be obtained before any study-related procedures are performed.
• Participants who require evaluation or treatment for worsening low back pain or trial-related adverse effects during or after trial completion will receive appropriate care at the study institutions in accordance with institutional policies. Participants may return for assessment at any time per the investigator’s instruction to monitor for delayed adverse events.
• Medical expenses for injuries or harms considered related to trial participation will be covered in accordance with the trial insurance contract and institutional policies. Procedures for reporting, evaluating, and compensating trial-related harms will follow the terms of the insurance and applicable regulations.

### Participants, interventions, and outcomes

#### Study setting.

This multicenter clinical trial will be conducted at two hospitals: Catholic Kwandong University International St. Mary’s Hospital and Wonkwang University Korean Medicine Hospital. This study will recruit 46 participants. Each institution will enroll participants in a 1:1 ratio across both groups, with 12 participants being recruited at the Catholic Kwandong University International St. Mary’s Hospital and 34 participants at the Wonkwang University Korean Medicine Hospital. Site-specific recruitment targets were allocated a priori based on each site’s expected patient flow, feasible recruitment capacity (e.g., average outpatient volume and anticipated enrolment rate), and operational considerations. In addition, we established a minimum target of 12 participants for the smaller site to support stable estimation of variability (rule of 12) [14]. An unequal distribution of participants across sites was considered acceptable because the overall target sample size (46 participants) was determined to achieve the planned statistical power, and the impact of allocation on power has been examined in multicenter/clustered trial settings [15].

### Eligibility criteria

**Inclusion criteria:**

- Adults aged 19–70 years
- Patients with non-acute LBP for ≥ 3 weeks with an average Numeric Rating Scale (NRS) score ≥4 over the past week
- Patients with a radiograph-based diagnosis of malposition
- Participants who voluntarily provided written informed consent

**Exclusion criteria:**

• Patients diagnosed with serious specific conditions that may cause LBP, including spinal tumor metastasis, acute fracture or dislocation, or moderate-to-severe scoliosis
• Patients with structural abnormalities of the lumbar spine (e.g., sacralization or lumbarization), a history of osteoporosis, or suspected fracture
• Patients who have undergone lumbar surgery within the past 3 months
• Patients with other chronic diseases that may interfere with treatment effects or result interpretation (including chronic renal failure)
• Patients with progressive neurologic deficits or severe neurologic symptoms, including cauda equina syndrome
• Patients with internal fixation or stabilizing devices from previous lumbar surgery
• Patients with contraindications to interferential current therapy (ICT), including cancer-related pain or implanted pacemaker
• Patients currently taking steroids, immunosuppressants, psychiatric medications, or other medications that could affect study outcomes
• Patients who have received Chuna manual therapy, strong opioid analgesics, anesthetic drugs, or other invasive treatments (including acupuncture or injections) within the past week
• Patients who have undergone lumbar nerve block procedures within the past 2 months
• Patients who are pregnant, breastfeeding, or planning pregnancy
• Patients expected to undergo surgery, procedures, or treatments that may influence outcome assessment during the 4-week intervention period
• Individuals considered inappropriate for trial participation at the discretion of the investigators

### Discontinuation and withdrawal criteria

• Participants are not allowed to take the following medications during the study period:
  ◦ Strong opioid analgesics or local anesthetics outside the study clinician’s prescription. However, non-steroidal anti-inflammatory drugs, muscle relaxants, and other medications prescribed as needed may be permitted based on the clinician’s judgment(See Concomitant care and rescue medication below)
  ◦ Any other medication considered by the study clinician to pose a risk to the patient or influence the study outcomes
• Participants who request discontinuation of the intervention or withdraw their consent to trial participation
• Occurrence of a serious adverse event that is considered by the investigator as sufficient grounds for discontinuation
• Identification of a major protocol violation during the trial, including a newly discovered violation of inclusion or exclusion criteria
• Participants who fail to attend at least 5 of the 8 scheduled treatment sessions
• Any other circumstance considered by the principal investigator or study clinician as sufficient grounds for trial discontinuation

### Early treatment criteria

• If a participant’s symptoms improve during the trial and they request discontinuation, with the study clinician determining that further intervention is unnecessary. Participants who are terminated early for this reason will not be classified as discontinuation or dropout cases and will be considered fully compliant with treatment.

### Interventions

#### Experimental group (Group I: usual care plus Chuna manual therapy intervention).

Participants in the experimental group will receive Chuna manual therapy in combination with usual care twice per week for 4 weeks.

*Chuna Manual Therapy*: Chuna manual therapy will be administered by licensed Korean medicine doctors who have graduated from an accredited Korean medicine medical college and are experienced in Chuna clinical practice. Based on the radiograph-based malposition diagnosis, individualized correction techniques will be applied following standardized Chuna treatment techniques developed through prior research and expert consensus [16]. In case more than one correction technique is available for a specific malposition within the standardized operating procedures (SOPs), the clinician will select the most appropriate method from the predefined SOP framework [16] based on the specific anatomical presentation, ensuring both clinical relevance and methodological consistency. If the clinician determines that additional Chuna manual therapy to non-lumbar areas such as the pelvis is necessary, the procedure may be performed and recorded in the case report form. Chuna procedures absent from the predefined SOPs may not be applied to the lumbar spine. Prior to Chuna manual therapy, ultrasonography will be used to confirm the exact lumbar level for accurate contact. Each Chuna manual therapy session will last ≈15–20 min.

*Usual Care* ICT will be applied to the lumbar region (device model STT-570, StraTek, South Korea or EF-160, OG Giken Co, Japan). Briefly, four ICT pads will be attached in a crossed configuration around the most painful lumbar region that allows convergence of the interferential current at the pain site. ICT will be applied for 15 min using medium frequency alternating current (4000–4100 Hz). Treatment intensity will be adjusted based on the patient’s sensation to avoid discomfort or pain. Additionally, at every visit, participants will receive 15-min education sessions and guided self-exercise for LBP relief and prevention. These include instructions on proper posture and ergonomic habits as well as pelvic tilt exercises, lumbar rotation without weight-bearing, knee-to-chest exercises, and trunk lifting. To ensure the standardization of the control group intervention across participating institutions, a rigorous SOP was established. Identical educational materials for self-exercise and lifestyle modification were distributed to all sites, and all researchers and practitioners followed the SOP to provide uniform instructions for posture, ergonomic habits, and specific exercises. This protocol was designed to minimize institutional variability and maintain the consistency of the usual care provided to all participants. To ensure the consistent delivery of usual care, a checklist is used at every visit to monitor whether all prescribed interventions (ICT and exercise/ education) are appropriately administered according to the SOP. This monitoring procedure ensures that the standard of usual care remains identical for participants in both Group I and Group II throughout the trial.

### Control group (Group II: usual care intervention)

Participants in the control group will only receive usual care. Usual care will comprise ICT, exercise guidance, and lifestyle education, which will be provided twice per week for 4 weeks. This group will not receive Chuna manual therapy.

### Rescue medication

From the screening visit onward, if intolerable pain occurs, the study investigator may allow the use of non-opioid rescue medications—specifically limited Non-steroidal anti-inflammatory drugs (NSAIDs) and skeletal muscle relaxants—while opioid analgesics and anesthetics are prohibited. To reduce adverse effects associated with rescue medication, gastroprotective agents may be co-administered. Whenever rescue medication is taken, the date and dose must be recorded in the Case Report Form (CRF)

*Permitted classes of rescue medication* Skeletal muscle relaxants, Anticonvulsants, NSAIDs, non-opioid analgesics, Agents for neuropathic pain, Antidepressants, Antipyretics, Weak opioids, Gastroprotective agents

### Outcomes

#### Primary outcome.

The primary outcome will be the change in the NRS score for LBP from baseline (Visit 2) to Week 4 (Visit 10) and Week 12 (Visit 11).

#### Secondary outcomes.

Patient-reported outcomes: Changes from baseline (Visit 2) to Week 4 (Visit 10) and Week 12 (Visit 11) in the following measures will be evaluated: NRS score for leg pain, Oswestry Disability Index (ODI), Roland Morris Disability Questionnaire (RMDQ), and EuroQol-5 Dimension (EQ-5D).

Physical Examination: Thoracolumbar range of motion will be assessed at baseline (Visit 2), Week 4 (Visit 10), and Week 12 (Visit 11), including flexion, extension, lateral flexion (right and left), and rotation (right and left).

Radiographic examination: Standardized L-spine standing anteroposterior and lateral radiographs will be obtained at baseline (Visit 2), Week 4 (Visit 10), and Week 12 (Visit 11). Relative segmental angular values (flexion, extension, lateral bending, rotation) and malposition diagnosis values for each vertebra will be compared.

#### Exploratory outcomes.

Rate of additional procedures or surgery: At 8 weeks after treatment completion, we will assess the proportion of participants in each group who undergo additional LBP-related invasive medical procedures, including injection therapy (such as nerve block procedures), radiofrequency ablation, use of strong opioid analgesics, and lumbar surgery.

Rate of early treatment termination: The proportion of participants in each group who meet early termination criteria due to symptom improvement.

Rescue medication analysis: Between-group comparison of the type, dose, and timing of rescue medication use.

Additionally, we will assess the treatment responder rate, i.e., the proportion of participants who achieve ≥ 2-point reduction in LBP NRS from baseline. Regarding safety assessment, all adverse events will be recorded at every visit. Further, we will evaluate abnormalities in physical examination and vital signs, as well as subjective or objective abnormal responses.

### Sample size calculation

The sample size for this study was calculated based on Cohen’s effect size derived from previously reported results [17]. In the prior study, the LBP NRS scores in the experimental and control groups were 3.02 ± 1.72 and 1.36 ± 1.75 (p < 0.001), respectively. Based on these values, the calculated effect size (Cohen’s d) was 0.96. Using a two-tailed test with a significance level (α) of 0.05 and 80 percent power (β), the following formula was applied: Here, Zα/2 is 1.96 for a two-tailed test at α = 0.05, and Zβ is 0.84 for 80 percent power. Based on this calculation, a minimum of 18 participants per group would be required. Anticipating a dropout rate of 20%, the final sample size was set at 23 participants per group, yielding a total of 46 participants.

### Recruitment

Participants will be recruited through advertisements approved by the Institutional Review Boards (IRBs) of each center. Recruitment notices will be posted on bulletin boards and in outpatient clinics within each center.

### Randomization and blinding

#### Sequence generation and allocation concealment.

Randomization will be performed using a computer-generated random allocation sequence created by an independent statistical expert. Stratified block randomization will be used, with stratification by study site; subsequently, participants will be assigned to each group in a 1:1 ratio. Allocation results will be placed in sealed, opaque envelopes and delivered to investigators by the study coordinator. Allocation will remain concealed until the moment of participant registration. The random allocation sequence will be held only by the independent statistician and the independent allocator; personnel who enroll participants and those who assign interventions at the sites will have no access to the sequence and will open the next sequentially numbered, opaque, sealed envelope only at the time of participant registration, with the action/time recorded.

#### Blinding.

Given the nature of the interventions, treatment providers and participants could not be blinded. However, outcome assessors and statistical analysts performing data analysis will remain blinded to group allocation.

### Data collection and management

All data will be recorded in electronic Case Report Forms. Regular monitoring will be conducted to verify data accuracy and completeness. Data management will comply with IRB policies and regulatory requirements. All data will be anonymized to protect personal information.

### Statistical methods

Primary analyses will follow the intention-to-treat principle using the full analysis set (FAS), defined as all randomized participants with at least one post-baseline efficacy assessment and analyzed according to assigned groups. The per-protocol set (PPS), defined as participants without major protocol deviations who complete the primary endpoint, will be used for supportive analyses. The safety set will include all participants who receive ≥1 session of the allocated intervention.

Demographic and baseline characteristics will be summarized using descriptive statistics. Continuous variables will be presented as mean ± standard deviation or median and interquartile range depending on the normality of data distribution. Categorical variables will be presented as frequency and percentage. Between-group comparisons of continuous variables will be performed using the independent t test or Wilcoxon rank sum test. Between-group comparisons of categorical variables will be conducted using the chi-square test or Fisher’s exact test. The full analysis set will be the primary analysis population, while the per protocol set will be used as a supportive analysis.

All statistical tests will be two-tailed with a significance level of 5%; further, two-sided 95% confidence intervals will be presented. The primary efficacy outcome will be the change in the LBP NRS score from baseline. Within-group comparisons will be performed using the paired t test or Wilcoxon signed rank test. Between-group comparisons will be performed using an analysis of covariance model, with the baseline NRS score as a covariate. Here, we will report the least square mean difference, 95% confidence interval, and p-value.

Secondary efficacy outcomes will include changes in the NRS score for leg pain, ODI, EQ-5D, and RMDQ from baseline. These will be analyzed using the same approach as the primary outcome. Exploratory effectiveness outcomes include the rate of additional invasive procedures or surgery and the rate of early treatment termination. Between-group comparisons for proportions will be conducted using the chi-square test or Fisher’s exact test. Between-group comparisons of the rescue medication type and frequency will be performed using the independent t test or Wilcoxon signed rank test. Treatment responders will be defined as participants with a ≥ 2 reduction in the NRS score from baseline, which represents the minimal clinically important difference. Appropriate post hoc analyses will be conducted based on the responder group.

Primary analyses will use the FAS, with PPS analyses as supportive. For continuous outcomes in the FAS, missing data will be handled using a mixed model for repeated measures (MMRM), which uses all available repeated-measures data without ad hoc imputation. The primary analysis will be conducted under the missing-at-random (MAR) assumption, while non-continuous outcomes will be analyzed without imputation. Sensitivity analyses will include complete-case analyses and responder analyses to assess robustness; responders are defined a priori as participants with a ≥ 2-point reduction in LBP NRS from baseline. In addition, sensitivity analyses under alternative missing-data assumptions (e.g., multiple imputation with delta adjustment) will be performed to assess the robustness to potential departures from the MAR assumption.

Safety evaluation will include descriptive statistics for the number of adverse events, number of participants experiencing adverse events, severity, and causality; additionally, non-parametric methods may be applied if necessary. Statistical analyses for this study will be performed after study completion. An interim analysis is not planned for this study

### Trial status

This manuscript is based on protocol version 1.1, dated June 04, 2025. Participant recruitment began on August 26, 2025 and the trial is currently ongoing. Study interventions are being delivered according to the protocol, but no data have yet been analyzed. Recruitment and follow-up are expected to be completed by approximately June 30, 2026.

## Discussion

This rigorously designed randomized controlled trial will evaluate the clinical effectiveness and safety of Chuna manual therapy guided by radiograph-based malposition diagnosis in patients with non-acute LBP. The study findings are expected to elevate the level of scientific evidence for Chuna manual therapy and crucially contribute toward the objectivity and reproducibility of treatment.

### Core significance of this study: evaluation of the clinical validity of diagnostic concepts

The diagnostic concept of “radiographic malposition” investigated in this study should be interpreted within the biopsychosocial (BPS) model, which is widely used in contemporary low back pain management. While previous clinical studies have primarily focused on the overall effectiveness of Chuna manual therapy [17], the clinical validity of the underlying diagnostic concepts used in manual medicine remains controversial [18].

The study aimed not to replace existing clinical guidelines but provide complementary data that may enhance therapeutic precision by quantifying radiographic alignment features using objective criteria, which are often not addressed in detail by standard protocols. We propose that “malposition” should be viewed not as a fixed pathological deformity, but as a potential biomechanical marker reflecting the patient’s structural stress that may help inform standardized decisions about the direction and intensity of Chuna interventions.

By visualizing and quantifying individual biomechanical imbalances through objective digital markers, this study seeks to strengthen the assessment within the “biological” domain of the BPS model by incorporating quantifiable biomechanical measures. This approach is particularly significant given that recent guidelines often caution against over-interpreting static structural deviations due to their sometimes weak correlation with subjective clinical symptoms [19]. Ultimately, by observing the correlation between structural alignment changes and subjective clinical improvement (NRS), we aim to explore how structural alignment changes relate to clinical improvement and provide complementary evidence to support the scientific basis of Chuna manual therapy within an integrated pain management context.

### Efforts to ensure objectivity through standardized procedures

Another strength of this study is the active use of SOPs to improve reliability. In the diagnostic process, standardized radiographic positioning and diagnostic methods based on prior research and expert consensus will be applied. Similarly, standardized Chuna techniques based on the malposition type have been pre-specified in the protocol. By applying SOPs for both diagnosis and treatment, this will minimize bias and maximize reproducibility, which is a common challenge in clinical research regarding manual therapies. These procedures are expected to serve as foundational data for developing clinical guidelines and education in Chuna manual therapy.

### Clinical implications and future directions

The findings of this study will highlight the potential of “personalized manual therapy” for LBP. Rather than applying uniform treatment to all patients, individualized treatment effects will be verified based on radiograph-based subgroup classification, which may improve both patient satisfaction and adherence. Furthermore, by adopting a practical clinical research model in which Chuna manual therapy is provided as an add-on to clinically validated usual care, our findings may serve as key clinical evidence for expanding the scope of health insurance coverage for Chuna manual therapy from treatment to diagnostic application.

### Limitations

Despite these strengths, this study has inherent limitations. First, this study was designed as a pragmatic trial to evaluate the “add-on” effectiveness of Chuna manual therapy in a real-world clinical setting. Consequently, we did not employ a sham control group, which may introduce performance bias or expectation effects on the primary outcome, the NRS score. Consequently, non-specific effects (e.g., additional time, physical touch, and patient–provider interaction) cannot be fully separated from the specific effects of Chuna manual therapy. Establishing a validated “inactive” sham for manual therapy is technically challenging, and in the South Korean medical context—where Chuna manual therapy is a highly familiar, insurance-covered treatment—an imperfect sham could lead to unintentional unblinding and high dropout rates. To mitigate these inherent limitations of a pragmatic design, we incorporated objective radiographic markers (radiographic malposition) as key secondary outcomes. Unlike self-reported pain scores, these structural spinal alignments are less susceptible to placebo effects, providing more reliable evidence regarding the specific therapeutic mechanisms of Chuna manual therapy. Second, the relatively short intervention (4 weeks) and follow-up (8 weeks) periods may be insufficient for evaluating long-term effects. Third, although standardized positioning will be used to minimize error, radiographs represent only a static structural alignment and may not fully reflect three-dimensional spinal configuration or dynamic functional aspects. Fourth, although Chuna manual therapy can be applied to the spine and extremities in clinical practice, this study is limited to the lumbar spine; therefore, it will not evaluate the full diagnostic or therapeutic scope of Chuna manual therapy. Lastly, the protocol involves three radiographic assessments to monitor structural changes, which is more frequent than that in routine clinical practice for low back pain. While this frequency was necessary to investigate the therapeutic mechanisms of Chuna manual therapy, it presents a practical burden and potential ethical considerations regarding cumulative radiation exposure. Future studies should consider optimizing the number of imaging sessions or exploring low-dose protocols to balance diagnostic need with patient safety.

## Conclusion

This randomized controlled trial has been rigorously designed to evaluate the clinical validity and safety of radiograph-based diagnostic concepts in Chuna medicine with application of standardized interventions throughout the diagnostic and treatment phases. This study will provide essential foundational evidence for radiographic diagnostic methods in Chuna manual therapy and manual medicine, as well as contribute to establishing more effective and systematic manual therapeutic strategies.

### Quality control and data monitoring

To enhance study quality, independent monitoring will be conducted by the Clinical Trial Center of Kyung Hee University Korean Medicine Hospital, which is independent from the study institutions and research team. Throughout the study period, the CRO will oversee management of all trial-related files (case report forms, informed consent forms, adverse event reports) and perform regular monitoring to ensure compliance with the study protocol.

### Trial registration and protocol access

This study was publicly registered before initiation through the Clinical Research Information Service (CRIS) of the Korea Disease Control and Prevention Agency (https://cris.nih.go.kr/) to ensure transparency and prevent publication bias (Registration number: KCT0011042). The registered protocol is accessible to the public through the CRIS website. Any major protocol amendments during the study will be approved by the IRB and updated in CRIS.

### Dissemination

The study results will be submitted for publication in peer-reviewed academic journals and presented at national and international academic conferences.

## Supporting information

S1 Appendix Radiographic positioning and diagnostic criteria for malposition in Chuna medicine. This appendix describes the standardized radiographic acquisition methods (anteroposterior and lateral views) and the quantitative diagnostic threshold values used to define lumbar malpositions in Chuna medicine.
(DOCX)

S1 Checklist SPIRIT 2025 checklist of items to address in the randomized trial protocol. This checklist summarizes the reporting of all recommended items for clinical trial protocols according to the SPIRIT 2025 statement, with page numbers indicating where each item is addressed in this protocol.
(DOCX)

S2 Appendix Full trial protocol (original Korean version). (PDF)

S3 Appendix Full trial protocol (English translation version). (PDF)
