# Supplementary material for: The effectiveness of Chuna manual therapy based on radiographic malposition diagnosis in patients with non-acute low back pain: A study protocol for a randomized, assessor-blind, parallel-group, controlled trial
Source: PLoS One. 2026 May 11;21(5):e0347321. doi: 10.1371/journal.pone.0347321 (PMC13160342; doi:10.1371/journal.pone.0347321)
Supplement: S1 Appendix — This appendix describes the standardized radiographic acquisition methods (anteroposterior and lateral views) and the quantitative diagnostic threshold values used to define lumbar malpositions in Chuna medicine. (DOCX) [file pone.0347321.s001.docx]

**S2 Appendix. Radiographic positioning and diagnostic criteria for malposition in Chuna medicine**

1. **Radiographic acquisition method**
2. AP view
3. Patient preparation:

- The participant stands barefoot with the feet shoulder width apart; further, the pelvic center line is aligned with the center line of the radiographic equipment.
- The hips are positioned parallel to the radiographic equipment.

1. Positioning:

- The mid-sagittal plane of the pelvis is aligned with the radiographic center line and the hip is centered in the radiographic field. The participant flexes and extends the head twice, followed by assumption of a neutral position.
- The participant closes and opens the eyes and looks naturally forward. Abnormal pelvic posture (for example, left–right tilt) is not artificially corrected.

1. **Beam location and distance:**

- The radiographic beam is centered at the L3 level at a standard source-to-image distance of 101.6 cm (40 inches).

- The beam angle is generally maintained horizontally

1. Lateral view
2. Patient preparation and positioning:

- The participant stands with the feet shoulder width apart and maintains the shoulders and pelvis perpendicular to the radiographic device.
- The participant moves the head twice to set a neutral head posture.
- The arms are crossed in front of the chest to minimize changes in the pelvic or thoracic posture without correction of the abnormal lumbar posture.

1. Beam location and distance:

- The radiographic beam is centered at the L3 level at a standard source-to-image distance of 101.6 cm (40 inches).

- The beam angle is generally maintained horizontally.

1. **Diagnostic criteria for malposition in Chuna medicine**

Malposition diagnosis in Chuna medicine is based on the relative angular relationship of each superior vertebral body to the immediately inferior vertebral body at each segmental level. Quantitative cut-off values for determining the presence of malposition were derived from previous research. Segments demonstrating an angular value that exceeds the diagnostic cut-off threshold are classified as having malposition at that level (Fig S1 in S2 Appendix).

For rotation malposition at L5, given the lack of a lower lumbar segment to reference, the right–left distance difference between the sacral ala and the S2 tubercle is used only when the measured value falls within the normal range defined in previous research. If the value falls outside the normal range, the measurement will be recorded in the case report form; however, it will be treated as missing for diagnostic and statistical purposes.


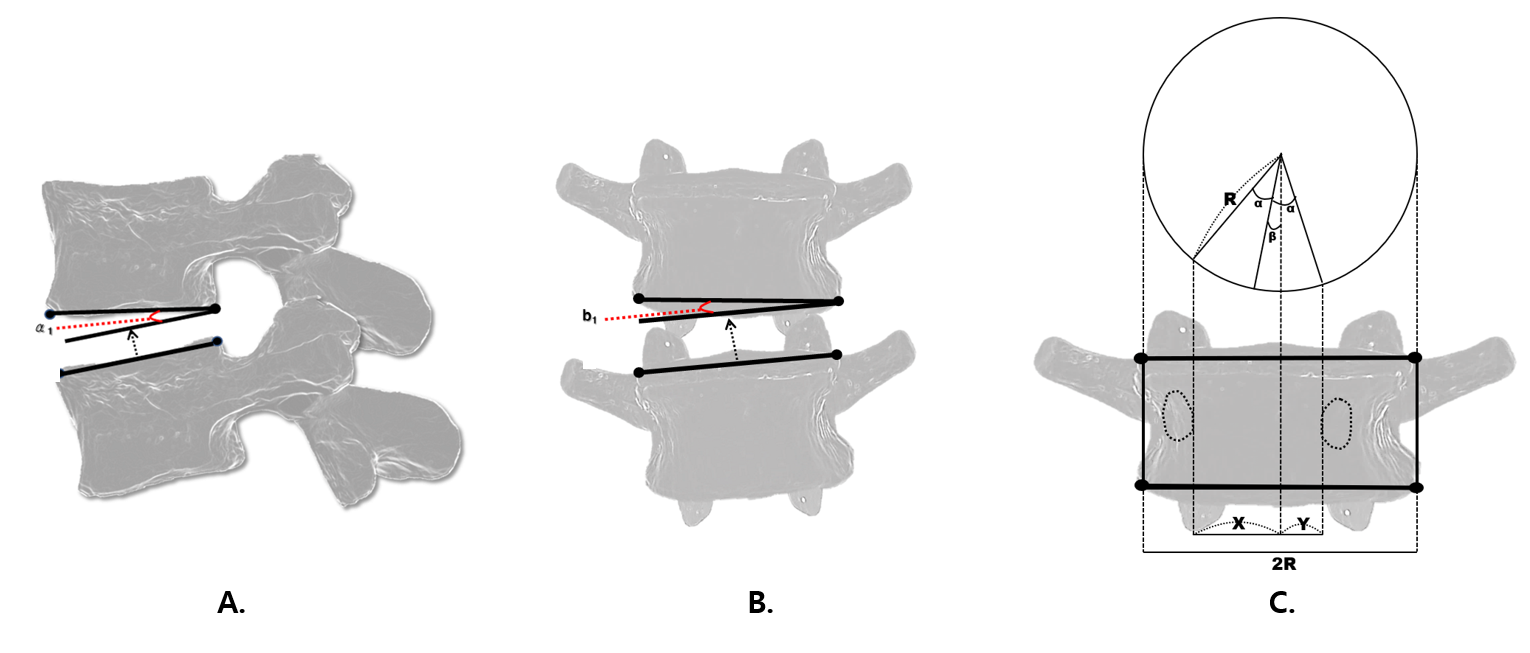


**Fig S1. Methods for measuring malposition angles.** (A) flexion/extension, (B) lateral bending, and (C) rotation angle. a in (A): flexion/extension angle; b in (B): lateral bending angle; β in (C): rotation angle.

**Appendix Table 1. Diagnostic threshold values for malposition in Chuna medicine**

|  | Vertebral body | | | | |
| --- | --- | --- | --- | --- | --- |
|  | L1 | L2 | L3 | L4 | L5 |
| Flexion(°) | 0.64 | 3.56 | 5.29 | 8.90 | 8.53 |
| Extension(°) | 10.46 | 14.95 | 17.61 | 20.76 | 24.47 |
| Lateral bending(°) | 2.21 | 2.10 | 1.92 | 2.06 | 2.31 |
| Rotation(°) | 9.49 | 5.19 | 4.59 | 5.87 | 7.13 |
