# Supplementary material for: The effectiveness of Chuna manual therapy based on radiographic malposition diagnosis in patients with non-acute low back pain: A study protocol for a randomized, assessor-blind, parallel-group, controlled trial
Source: PLoS One. 2026 May 11;21(5):e0347321. doi: 10.1371/journal.pone.0347321 (PMC13160342; doi:10.1371/journal.pone.0347321)
Supplement: S2 Appendix — (PDF) [file pone.0347321.s003.pdf]

임 상 시 험 계 획 서

비급성 요통 환자에서 단순방사선영상 변위 진단에 따른 추나의학적 치료 효과 탐색
(무작위배정, 평행 설계, 평가자 맹검)

The Effectiveness of Chuna Manual Therapy Based on Radiographic Malposition Diagnosis in Patients with Non-acute Low back Pain
(A randomized controlled, two-arm, parallel study, assessor-blind)

Version 1.1 (DATE: 2025.06.04)

▶ 임상시험정보

비급성 요통 환자에서 단순방사선영상 변위 진단에 따른 추나의학적 치료 효과탐색 (무작위배정, 평행 설계, 평가자 맹검)

The Effectiveness of Chuna Manual Therapy Based on Radiographic Malposition Diagnosis in Patients with Non-acute Low back Pain (A randomized controlled, two-arm, parallel study, assessor-blind)

▶ 임상시험실시기관 및 시험자정보

시험조정기관: 가톨릭관동대학교 국제성모병원(인천광역시 서구 심곡로 100번길 25)
시험실시기관: 가톨릭관동대학교 국제성모병원(인천광역시 서구 심곡로 100번길 25)
원광대학교 한방병원(전라북도 익산시 무왕로 895)

시험조정자

이진현 KMD, PhD 가톨릭관동대학교 의과대학 의학과/
가톨릭관동대학교 국제성모병원 한의과

임상시험책임자

이진현 KMD, PhD 가톨릭관동대학교 국제성모병원 한의과

하원배 KMD, PhD 원광대학교 한방병원 한방재활의학과

▶ 약어 및 용어의 정의

I 군(통상치료+추나요법군)	4 주간 8 회의 통상적인 통상치료 및 추나요법을 시행하는 군
II 군(통상치료군)	4 주간 8 회의 통상치료만을 중재로 시행하는 군
경피적 전기신경자극치료 (경근중주파요법, ICT)	Interferential Current Therapy
추나요법	한의사에 의해 수행되는 한국식 수기 치료법
중재군	Arm, 치료군의 조합에 따라 분류한 군
치료군	Treatment group, 시험약의 구성에 따라 분류한 군
CBP	Chiropractic Biophysics
DICOM	Digital Imaging and Communications in Medicine
BMI	Body mass index 체질량 지수
ODI	oswestry disability index 오스웨스트리 기능 장애 평가
RMDQ	Roland Morris Disability Questionnaire
EQ-5D	European Quality of Life 5 Dimension
ROM	Range of motion 관절 가동범위
IRB	Institutional Review Board, 임상시험심사위원회
AE	Adverse Event, 이상반응
ITT	Intent-to-treat, 전체 환자
PP	Per-protocol, 프로토콜 기반 모델
PR	The proportion of responders

목 차

1. 임상시험의 명칭 13
2. 임상시험실시기관명 및 주소 13
3. 임상시험 의뢰자 13
4. 임상시험의 목적 및 배경 13
5. 시행되는 중재에 대한 설명 16
6. 대상자의 포함기준, 제외기준, 목표한 대상자의 수 및 그 근거 25
7. 시험기간 26
8. 시험방법 26
9. 관찰 및 검사 항목 28
10. 중지/탈락 및 임상시험 조기 종료 기준 30
11. 치료순응도 30
12. 구제약물 31
13. 임상시험 조기 중단 기준 31
14. 임상시험 종료 후 대상자의 진료 및 치료기준 31
15. 유효성평가 및 안전성 평가 31
16. 통계분석 37
17. 이상반응의 보고방법 및 평가기준 39
18. 자료 관리 43
19. 대상자 동의서 양식 43
20. 피해자 보상에 대한규약 43
21. 대상자의 안전 보호에 관한 대책 43
22. 기타 임상시험을 안전하고 과학적으로 실시하기 위하여 필요한 사항 44

<참고문헌> 45

▶ 임상시험요약표

제 목	비급성 요통 환자에서 단순방사선영상 변위 진단에 따른 추나의학적 치료 효과 탐색(무작위배정, 평행 설계, 평가자 맹검)<br>The Effectiveness of Chuna Manual Therapy Based on Radiographic Malposition Diagnosis in Patients with Non-acute Low back Pain (A randomized controlled, two-arm, parallel study, assessor-blind)
목 적	비급성 요통 환자에서 단순방사선영상 변위 진단에 따른 추나의학적 치료 효과에 대한 근거 확보
임상시험 조정자	가톨릭관동대학교 의과대학 의학과/가톨릭관동대학교 국제성모병원 한의과 이진현
임상시험 책임자	가톨릭관동대학교 국제성모병원 한의과 / 이진현<br>원광대학교 한방병원 한방재활의학과 / 하원배
임상시험담당자	[별지1]서식 연구자 및 담당자의 성명 및 직명 참고
임상시험 의뢰자	연구자주도임상시험
실시기관	가톨릭관동대학교 국제성모병원, 원광대학교 한방병원
분석기관	가톨릭관동대학교 의과대학 의학과/가톨릭관동대학교 국제성모병원 한의과
임상시험 대상자 및 연구방법	**1. 포함기준(Inclusion Criteria)**<br>① 연령: 만 19세 이상 만 70세 이하<br>② 비급성(발병 후 3주이상 경과) 요통으로 최근 일주일간의 평균 통증 강도 Numeric Rating Scale (NRS) ≥4 인 자<br>③ 단순방사선 영상을 통해 추나의학적 변위가 있는 것으로 확인된 환자<br>④ 임상시험 참여에 자발적으로 동의하고 동의서에 서명한 자<br><br>**2. 제외기준(Exclusion Criteria)**<br>① 요통의 원인이 될 수 있는 심각한 특정 질병을 진단 받은 경우 (종양의 척추 전이, 급성 골절 및 척추탈구, 중증도 이상의 척추의 측만증 등)<br>② 요추의 구조적 이상 (sacralization, lumbarization), 골다공증의 기왕력이 있거나, 골절이 의심되는 경우<br>③ 요추 수술 후 3개월 이내의 환자인 경우<br>④ 치료효과나 결과의 해석을 방해할 수 있는 다른 만성적인 질환이 있는 경우 (만성 신부전)<br>⑤ 진행성의 신경학적 결손이 있거나, 마미증후군 등의 심각한 신경학적 증상이 동반된 경우<br>⑥ 요추 수술로 내부 고정 및 안정 장치가 있는 경우<br>⑦ ICT 치료의 금기증인 경우(암성 동통, 심박조정기 착용환자 등)<br>⑧ 현재 스테로이드제제, 면역억제제, 정신질환 약물 또는 연구 결과에 영향을 줄 수 있는 기타 약물을 복용하고 있는 경우<br>⑨ 최근 1주일 이내에 추나치료를 받았거나, 강한 마약성 진통제 및 마취제 등의 약물을 투여받은 경우 또는 침, 주사 등 침습적 치료를 받은 경우<br>⑩ 최근 2개월이내에 요추부에 신경차단술 시술을 받은 경우<br>⑪ 임신중이거나 모유 수유중인 자 또는 임신을 계획 중인 경우<br>⑫ 시술기간 (4주) 동안 담당의사의 지시 없이 연구 결과 판정에 영향을 미칠 수 있는 수술이나 시술, 약물 등의 치료를 받을 예정인 경우<br>⑬ 기타 연구담당자가 판단하기에 임상시험 참여에 부적절 할 것으로 판단한 자

3. 중지 및 탈락 및 임상시험 조기 종료기준

○ 중지/탈락 기준

① 대상자가 중재방법 유효성을 평가하는데 영향을 줄 것으로 예상되는 아래와 같은 의약품은 스크리닝 이후부터 임상시험 완료시까지 복용할 수 없다.

· 강한 마약성 진통제(strong opioid), 임상시험 담당의가 투약과는 별도로 투여된 국소 마취제 및 스테로이드제제.
단, 임상의의 판단을 기초로 필요에 의해 처방되는 소염진통제, 근이완제 등은 투약가능 (11. 구제약물 참조)

· 기타 임상시험담당의가 판단하기에 환자에게 위험을 초래하거나 연구의 평가결과에 비뚤림을 유발할 수 있을 것이라 판단되는 약물

② 대상자가 임상시험 중 임상시험 중재 시술의 중단을 요구하거나, 시험참여 동의를 철회하는 경우

③ 중대한 이상반응이 발생하여 시험자가 시험을 계속할 수 없다고 판단하는 경우

④ 임상시험 중 포함/제외 기준 등 중대한 계획서 위반 사항이 새롭게 발견되는 경우

⑤ 임상시험 조기 종료 기준에 부합하지 않지만 예정된 8 회 치료 중 5 회 이상 참여를 시행치 아니한 경우

⑥ 기타 이유로 대상자가 임상시험을 지속하기 어려울 것으로 보여 임상시험책임자/담당자가 시험을 중지하여야 한다고 판단한 경우

○ 조기 종료

① 임상시험 기간 중 증상이 호전되어, 환자 개인적 요청이 있고 임상시험 담당 의료진의 판단에 의해 더 이상의 시술이 필요 없다고 판단되는 경우에는 임상시험을 조기 종료 할 수 있다.

② 임상 시험이 조기 종료된 대상자는 중지/탈락에 포함시키지 않으며, 치료에 모두 순응한 것으로 평가한다.

4. 대상자 수

- 총 대상자수: 46명 : 다기관 모집
  · 가톨릭관동대학교 국제성모병원 : 12명
  · 원광대학교 한방병원 : 34명

군별	대상자 수	예정된 시술 중재
I	23명	통상치료 (ICT+운동지도) + 추나요법
II	23명	통상치료 (ICT+운동지도)

I. 군 :
- 2회/주 간격으로 4주간 총 8회의 통상치료(ICT + 운동 및 생활습관 교정) + 추나요법을 시행

II. 군 :
- 2회/주 간격으로 4주간 총 8회의 통상치료(ICT + 운동 및 생활습관 교정)만 시행

- 설정근거
- 본 연구의 표본 크기는 선행 유사연구에서 보고된 결과를 바탕으로 Cohen의 효과 크기를 기반으로 산출하였다. 선행연구에서 추나요법+통상치료 그룹의 요통 NRS 점수는 3.02±1.72였으며, 통상치료 그룹은 1.36±1.75였다($p<0.001$). 이 데이터를 바탕으로 효과 크기(Cohen's d)를 계산한 결과, d=0.96이었다. 양측 검정(two-tailed test), 유의수준(α) 0.05, 검정력(power) 80%를 적용하여 다음 공식을 사용하였다:

$$n = 2 \times [(Z_{\alpha/2} + Z_{\beta})^2/d^2]$$

여기서 $Z_{\alpha/2}$는 유의수준 0.05에 대한 양측 검정 Z값인 1.96이고, Z_{β}는 검정력 80%에 대한 Z값인 0.84이다. 계산 결과, 각 그룹당 필요한 최소 표본 수는 18명으로 산출되었다. 연구 과정에서 예상되는 20%의 탈락률을 고려하여, 최종적으로 각 그룹당 23명, 총 46명의 대상자를 모집하기로 하였다.

5) 연구 일정 및 방법

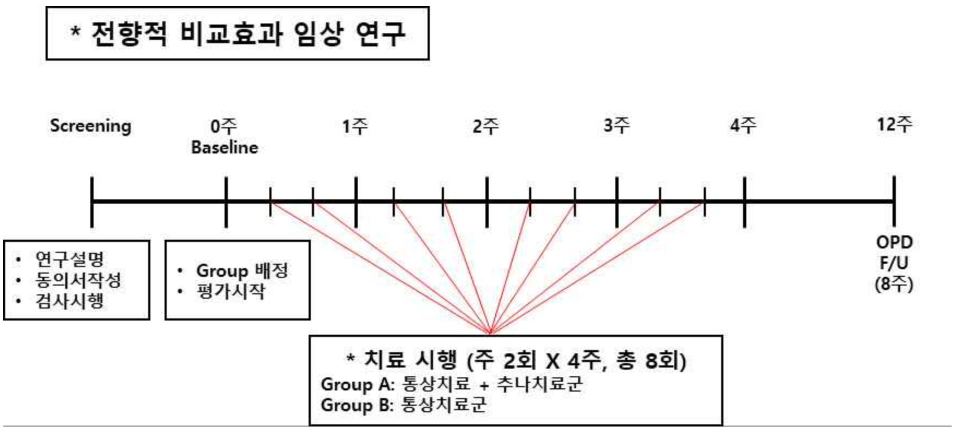

1) Screening Visit(visit 1, day –7 ~ 0)

대상자의 적합성 스크리닝 검사는 서면동의서를 작성한 대상자에 한해 실시된다. 검사 결과 다음 항목에 의해 임상적으로 유의한 이상이 있는 대상자는 제외한다.
(단 screening visit에서 연구 등록이 확정된 경우 해당일에 baseline test을 동시 수행 가능)

① 인구학적 조사 : 성별, 나이, 신장, 체중, BMI, 흡연력, 음주력
② 병력 조사 : 요통의 기간, 약물복용, 과거 병력
③ 신체검사, 활력징후
일반상태, 영양상태, 피부/점막, 눈, 이비인후계, 갑상선, 폐, 심장/순환계, 복부, 신장/비뇨생식계, 신경/정신계, 척추/사지/종양, 말초순환, 림프계 등에 대한 문진 및 신체검사를 시행한다.
④ 혈압(좌위), 맥박수, 체온을 측정한다(반드시 급격한 체위 변동 없이 5분 이상 좌위를 유지한 상태에서 혈압과 맥박수를 측정).
⑤ 영상검사 및 추나 변위 유무 파악
· 6-1.임상시험에 활용되는 단순방사선영상 기반 추나의학적 진단 방법 항목의 Chiropractic Biophysics표준화 절차에 따라 L-spine AP & Lateral standing view 영상을 촬영.
· 영상 DICOM 파일을 바탕으로 추나의학적 변위 유무 및 유형을 확인
· 선정/제외기준 확인 : NRS검사, 기존 병력, 치료력 등을 확인하여 선정/제외 기준을 평가

2) Baseline Visit(visit 2, day 0) - Visit 1과 같은날 시행가능

Screening test를 통과하여 임상시험에 참여하게 된 환자의 경우블록 무작위배정을 실시하여 대상자 번호를 부여한 뒤 다음과 같은 평가를 진행한다.

(1) 환자의 주관적 증상, 기능 제한, 삶의 질에 대한 설문평가
· NRS (요통 및 하지통으로 구분지어 측정)
· Oswestry disability index (ODI)
· European Quality of Life 5 Dimension (EQ-5D)-5L,
· Roland Morris Disability Questionnaire (RMDQ)]
(2) 이학적 평가
· 요추의 관절가동범위 검사 (ROM)
: Flexoin, Extension, lateral flexion (Rt. & Lt.), Rotation (Rt. & Lt.)
(3) 활력징후
- 혈압(좌위), 맥박수, 체온을 측정하며, 측정 전에는 반듯이 급격한 체위 변동 없이 5분 이상 좌위를 유지한다.

3) 치료방문 (통상치료+추나요법군, 통상치료군 동일 방문 - 총 8회)

(1) 통상치료+추나요법군 (I군, visit 3, 4, 5, 6, 7, 8, 9, 10)
- Baseline visit 직후부터 2회/주 간격으로 4주간, 총 8회 시행
(2) 통상치료군 (II군, visit 3, 4, 5, 6, 7, 8, 9, 10)
- Baseline visit 직후부터 2회/주 간격으로 4주간, 총 8회 시행
(3) 매 시술전 활력증후를 측정한다.

- 혈압(좌위), 맥박수, 체온을 측정하며, 측정 전에는 반듯이 급격한 체위 변동 없이 5분 이상 좌위를 유지한다.

(4) 시술전 이상반응을 확인한다.

- 이전 방문 이후 발생한 이상반응, 타각적 이상반응에 대해서 확인한다.

4) **<u>평가 방문 (I군, II 군 동일 - visit 2, 10, 11)</u>**

스크리닝을 통과한 모든 대상자는 baseline visit, 치료시행 종료 후(baseline 이후 4주) 모든 시술이 종결된 8주 (baseline 후 12주) 후 외래를 방문하여 아래 검사를 실시한다.

① 활력징후

② 환자의 주관적 증상 및 기능 평가

- 1차 유효성 평가 및 2차 유효성 평가를 시행

: 환자의 통증 강도 및 기능 평가 설문지 활용 NRS, ODI, EQ-5D-5L, RMDQ

③ 이학적평가

- 요추 ROM : Flexoin, Extension, lateral flexion (Rt. & Lt.), Rotation (Rt. & Lt.)

④ 영상평가

- 표준화 절차에 따른 L-spine AP & Lateral standing view 영상을 촬영.
- 촬영된 영상을 바탕으로, dicom labeling 프로그램을 통해 추나의학적 변위 유형 진단을 위한 추체별 상대적 각도(flexion, extension, lateral bending, rotation) 및 추나의학적 변위 진단 결과 값을 비교.

⑤ 탐색적 유효성 평가

연구 종료 후 추가적인 의과의 침습적 시술[주사치료(신경차단술) 고주파 열응고술 등] 시행 여부, 강한마약성 진통제 사용여부(strong opioid), 허리 수술 여부에 대한 조사를 시행

⑥ 이상반응 모니터링

5) **<u>조기 종료 방문 (Early Discontinuation Visit)</u>**

임상시험참여 후 중재를 1회 이상 실시한 이후 조기 종료 기준에 부합하여 임상시험이 종료된 환자는 마지막 시술 8주 뒤 시험기관을 일정 외 방문하여 평가방문을 시행한다 (단, 평가방문을 원칙으로 하되, 환자의 요청이 있는 경우 신체검사, 활력징후, 이학적평가, 영상평가를 제외한 최대한의 항목을 대해서는 전화 상담으로 대체할 수 있음). 중지/탈락기준에 의거하여 시험을 조기에 중지하거나 시험대상자가 탈락하는 경우에는 별도의 추적관찰 평가를 시행치 않는다.

연구에 활용되는 치료 기술 및 시험 방법

(참고) 중재에 대한 세부 정보는 6-2) 임상시험에 활용되는 치료 술기 항목 참조.

○ 통상치료 (경근 중주파 치료 + 운동 및 생활습관 교정)

(1) 경근 중주파 치료(간섭파 전류치료; Interferential Current Therapy, ICT)

- 2024년도 한국보건의료원(NECA)에서 발행된 의료기술재평가보고서에서 요통에 효과 및 안정성이 있다고 보고한 ICT를 통상치료로 활용
- 연구에 enrollment 된 이후 통증부위에 2회/주 간격으로 4주간, 회당 15분, 총 8회를 시행

(2) 운동 및 생활습관 교정

- 골반경사운동, 체중부하없이 허리돌리기, 무릎당기기, 상체들기 등의 운동 15분 정도 지도
- 요통의 예방자세, 올바른 작업자세와 관련한 환자 교육자료 제공 및 설명

○ 추나요법

(1) 시술 부위 및 방법

· Screening 방문시 확인된 요추의 변위 위치를 확인 후 해당 척추분절에 변위유형에 따른 SOP에 제시된 표준 추나기법을 적용 (필수 추나)

· 정확한 시술부위 적용을 위해 추나요법 시술 전 초음파 기기를 통해 예정된 시술 부위 확인 및 부위 표시

· 한 변위에 대한 복수 이상의 교정방법이 SOP에 제시되어 경우 시술자의 판단에 따라 적절한 기법을 적용

· 시술자의 판단에 요추를 제외한 다른 부위(골반)에 대한 추가적인 추나요법이 필요하다고 사료되는 경우 관련 기법을 시행할 수 있으며, 관련 내용을 증례기록서에 기록할 예정임 (단, 요추부에는 제시된 표준작업지침 이외의 추나 치료는 불가)

(2) 시술 시간 및 횟수

- 2회/주 간격으로 4주간, 총 8회 (1회치료당 15분 내외)의 치료를 시행.

평 가 방 법	1. 유효성 평가<br>▶ 1차 유효성 평가 : 요통에 대한 NRS<br>임상시험 참여 후 0주, 4주, 12주째 (visit 2, 10, 11) 에 임상시험에 참여하기 전과 비교하여 해당시점까지 시험대상자의 요통에 대한 NRS 평가를 시행한다.<br><br>▶ 2차 유효성 평가<br>1) 설문평가<br>· 0주, 4주, 12주의 하지통에 대한 NRS, Oswestry disability index (ODI), European Quality of Life 5 Dimension (EQ-5D), Roland Morris Disability Questionnaire (RMDQ) 평가를 시행한다.<br><br>2) 이학적 평가<br>· 0주, 4주, 12주 시점에 의 흉요추 ROM 평가를 시행하며, 굴곡(flexion), 신전(extension), 측굴(lateral flexsion –우,좌), 회전(rotation- 우,좌)로 나누어 시행한다.<br><br>3) 영상 평가<br>· baseline test(0주), 4주, 12주째시점에서 표준화 절차에 따른 L-spine AP & Lateral standing view 영상을 촬영<br>· 촬영된 영상을 바탕으로, 한국한의학연구원에서 제공하는 dicom labeling 프로그램을 통해 추나의학적 변위 유형 진단을 위한 추체별 상대적 각도(flexion, extension, lateral bending, rotation) 및 추나의학적 변위 진단 결과 값을 비교한다.<br><br>4) 탐색적 유효성 평가<br>① 추가 시술 또는 수술 시행율 평가<br>: 모든 임상시험 대상자를 대상으로 처치 종료 8주 후 최종 방문일에 허리통증과 관련한 의과의 침습적 시술[주사치료(신경차단술) 고주파 열응고술 등] 시행 여부, 강한마약성 진통제 사용여부(strong opioid), 허리 수술 여부를 조사<br>② 치료 조기 종결율 평가<br>: 증상 호전으로 치료 종결 기준에 부합하여, 치료가 조기 종결된 환자수를 기반으로 각 군별 연구 조기 종료 비율을 평가.<br>③ 구제 약물 사용 분석<br>: 각 피험자별 사용된 구제약물의 유형, 투여량, 투여시기에 대한 정보를 수집하여 군간 비교를 시행<br><br>5) 치료 반응군(responder), 비반응군(non-responder) 평가<br>① Baseline에서 0, 4, 12주 후 평가에서 minimal clinically important difference (CID)를 NRS의 감소량 2로 설정하여 치료에 반응한 집단과 비반응 집단을 구분<br>② 증상 호전으로 치료 조기 종료 기준에 부합하여 임상시험이 조기 종료된 경우를 치료 반응군으로 분류.<br><br>6) 안전성 평가<br>신체검사, 활력징후(Vital signs) 등의 이상, 자·타각 증상 등 이상반응을 평가한다.
통 계 분 석	1) 인구학적 정보 및 치료 전 특성에 대한 분석 방법<br>인구학적 정보 및 특성에 대해 기술 통계량을 기술한다. 연속형 자료는 정규성 만족 여부에 따라 관측치 수, 평균, 표준편차 또는 중앙값, 25%분위수, 75%분위수로 나타내고, 범주형 자료의 경우 빈도수와 백분율로 나타낸다. 연속형 자료에 대한 군간 평균차이 검정을 위하여 정규성 가정을 만족하면 Independent t-test 를 시행하고, 만약 정규성 가정을 만족하지 않으면 Wilcoxon's rank sum test 를 시행한다. 그리고 범주형 자료에 대하여 군간 차이 검정을 위하여 Chi-square test를 시행하거나 셀 기대도수가 5 미만인 경우가 25%를 넘을 경우 Fisher's exact test를 시행한다.<br><br>2) 유효성 평가<br>유효성 분석은 모든 분석 대상자군(FAS)를 주 분석군으로 하고, 순응 임상시험대상자군(PP sets)을

보조 분석군으로 병행하여 분석한다. 모든 통계 검정은 유의수준 5%에서 양측 검정으로 수행하는 것을 원칙으로 하며, 양측 95% 신뢰구간을 제시한다. 각 치료군별 및 방문별로 기술통계량을 제시하며, 기저치 대비 각 방문과의 차이에 대해서는 기술통계량 및 95% 신뢰구간을 제시한다.

가. 일차 유효성 평가 변수 분석

▶ 각 측정 시점의 요통과 관련하여 NRS 점수의 기저시점(0주) 대비 치료 종료(4주)와 추적관찰(12주)에서의 변화량에 대하여 치료군별 기술 통계량(시험대상자 수, 평균, 표준편차)을 제시

▶ 기저시점(0주) 대비 치료종료(4주) 시점에서의 요통의 NRS 변화량에 대한 각 군내 검정은 정규성 만족 여부에 따라 paired t-test 또는 wilcoxon signed rank test를 실시한다. 대조군 대비 치료군의 효과는 기저시점 대비 치료종료 시점의 NRS변화량에 대한 치료군을 effect로 하고, 기저시점 NRS 점수를 공변량으로 포함하는 ANCOVA model을 이용하여 분석한다. 대조군 대비 치료군간 least-square mean(LSM) 차이를 구하고, 그 차이에 대한 양측 95% 신뢰구간 및 p-value를 제시한다.

나. 이차유효성 평가변수분석

▶ 이차유효성 평가는 다음과 같은 항목을 시행하며, 일차 유효성 평가변수의 분석 방법을 준용해서 기저시점(0주) 대비 치료 종료(4주)와 추적관찰(12주) 사이의 변화량을 측정한다.

① 기저시점(0주) 대비 치료 종료(4주)와 추적관찰(12주)에서의 하지통증의 NRS 변화량

② 요통과 관련한 주관적 통증 강도 및 삶의 질 의 평가

- 기저시점(0주) 대비 치료 종료(4주)와 추적관찰(12주)에서의 ODI, EQ-5D, RMDQ의 변화량

다. 탐색적 유효성 평가

▶ 연구 종료 후 신경차단술의 추가 시술 또는 수술 여부와 관련한 평가

시술 종료 후 8주이내의 기간에 환자의 통증 제어를 위한 추가적인 시술 (예: 신경차단술)이 수행되었거나, 수술이 시행된 경우에 대해 조사를 시행하여, 이를 바탕으로 추가 시술율을 평가하고자 함. 군간 차이가 확인될 경우 사후 검증을 시행한다. 군간 추가 시술과 수술의 시행율 비교는 Chi-square test 또는 Fisher's exact test등을 실시한다.

추가 시술 시행율 = (추가적인 시술 또는 수술을 시행한 환자/전체 연구를 종료한 환자)*100

▶ 치료 조기 종결율 평가

예정된 시술이 종료되기 전 치료 종결 기준에 부합하여, 치료가 조기 종결된 경우 각 군별 연구 조기 종료 비율을 평가하고자 한다. 치료조기 종료율의 군간 비교는 Chi-square test 또는 Fisher's exact test 등을 실시한다.

연구 조기 종료 비율 = (연구가 조기 종료된 환자 / 전체 연구를 종료한 환자)*100

▶ 구제 약물 사용 분석

각 피험자별 사용된 구제약물의 유형, 투여량, 투여시기에 대한 정보를 수집하여 각 군 별 구제약의 종류에 따른 사용 횟수 및 사용량에 대한 군간 비교는 independent t test 혹은 Wilcoxon signed rank test를 시행한다.

라. Respondor 및 Non-responder 구분 및 Post-hoc analysis

▶ 치료 반응자의 정의

① Baseline에서 평가시일 까지의 NRS의 감소량을 기반으로 구체적인 minimal clinically important difference (CID)인 NRS 2를 기준으로 한다. 따라서 다음과 같이 정의한다.

NRS 2 이상의 통증감소가 이루어진 환자를 CID 로 규정하고 이를 바탕으로 치료반응집단 / 비반응 집단으로 구분

② 앞서 언급한 기준을 바탕으로, 증상 호전으로 인한 연구가 조기 종결된 경우 치료 반응 집단으로 합계한다.

▶ Post-hoc analysis

	결정된 Respondor 및 Non-responder의 기준을 기준으로 각 치료군 별 치료 결과차이에 대해 통계전문가를 통한 적절한 Post-hoc analysis를 시행한다.<br><br>3) 안전성 평가<br>이상반응 발생에 대해서 발생건수, 발생한 대상자수, 중증도(severity), 중재와의 인과관계를 용량군에 따라 기술통계학적으로 분석하고 필요에 따라 비모수적 방법을 적용할 수 있다.<br><br>4) 참고 (중대한 이상반응 보고 관련)<br>- 추나요법시술 후 예측되는 부작용으로는 시술부위 통증의 악화, 피로감 등이며, 드물게 신경손상 및 골절등이 발생할 수 있다. ICT의 경우 시술부위 피부 자극이나, 화상, 수포, 시술 후 불편감 등이 있을 수 있다. 본 연구에서 시술과 관련한 이상 반응의 범주는 이와 같은 내용을 기본으로 하며, 추나요법 시술과 관련한 이상 반응의 범주는 통증의 악화, 피로감, 골절, 신경손상으로 하며, 통상치료군의 경우 화상, 수포, 통증의 악화까지를 이상반응의 범주로 설정한다.

► 임상시험일정요약

Period	Screening	Baseline[1]	Treatment								Post Treatment	조기종료 중도탈락
방문	Visit 1	Visit 2	Visit 3	Visit 4	Visit 5	Visit 6	Visit 7	Visit 8	Visit 9	Visit 10	Visit 11	
day	-7~-0	0	1-3	4-6	7-10	11-14	15-18	18-21	22~24	25-28	81-84	
서면동의서	√											
인구학적 조사	√											
병력, 기타병력 및 약물투여력 조사	√											
스크리닝 검사 2)	√											
선정기준/제외기준 확인	√											
무작위배정 (등록번호부여)	√											
신체검사 및 활력징후 확인	√	√	√	√	√	√	√	√	√	√	√	√
약물투여력 확인 3)			√	√	√	√	√	√	√	√	√	√
치료: 통상치료 4)+ 추나요법군 5)			√	√	√	√	√	√	√	√		
치료: 통상치료군			√	√	√	√	√	√	√	√		
1차 유효성평가 6)		√								√	√	√
2차 유효성평가 7)		√								√	√	√
탐색적 유효성 평가 8)											√	√
이상반응 모니터링			√	√	√	√	√	√	√	√	√	√
안전성 평가			√	√	√	√	√	√	√	√	√	√

1) Screening test 및 Baseline test는 동일한 날짜에 시행이 가능함 (visit 1,2가 visit 1로 통합 가능). screening test와 baseline test가 분리되어 시행될 경우 screening test 후 Active Treatment까지 1~2주간 소요됨.
2) 인구학적 조사, 병력조사, 신체검사, 활력징후, 단순방사선영상을 통한 추나의학적 변위 유무 검사, 선정/제외 기준 파악
3) 치료 및 평가를 위한 방문시 매번 병용투여약물 투여력, 이상반응 모니터링을 수행한다.
4) 통상치료는 Interferential Current Therapy (ICT), 운동요법, 생활습관 교육으로 이루어짐.
5) 추나요법은 추나의학적 변위 진단의 부위 및 유형에 따라 표준화된 추나요법을 적용함.
6) 요통에 대한 Numeral rating scale NRS평가
7) 하지통에 대한 NRS, 오스웨스트리 기능장애 평가(Oswestry disability index; ODI)), 한국형 Roland Morris Disability Questionnaire (RMDQ) 평가지, 삶의 질(European Quality of Life 5 Dimension)평가, 이학적 평가(흉요추 관절가동범위 검사), 영상평가 (visit 2에서의 영상평가는 별도로 시행하지 않고 visit 1의 screening 검사로 대체함)
8) 최종 시술 종료 8주 이내에 추가적인 허리통증과 관련한 의과의 침습적 시술[주사치료(신경차단술) 고주파 열응고술 등] 시행 여부, 강한마약성 진통제 사용여부(strong opioid), 허리 수술 여부를 조사한다. 탐색적 평가는 조기 종료 환자에게는 시행하지 않으며, 중도탈락 환자의 경우 탈락 8주 후 해당 평가를 시행한다.

※ 주말 및 공휴일 등의 사유로 ±3일의 visit window가 허용될 수 있다

1. 임상시험의 명칭

비급성 요통 환자에서 단순방사선영상 변위 진단에 따른 추나의학적 치료 효과 탐색(무작위배정, 평행 설계, 평가자 맹검)
The Effectiveness of Chuna Manual Therapy Based on Radiographic Malposition Diagnosis in Patients with Non-acute Low back Pain (A randomized controlled, two-arm, parallel study, assessor-blind)

2. 임상시험실시기관명 및 주소

임상시험조정기관: 가톨릭관동대학교 국제성모병원 (인천광역시 서구 심곡로 100 번길 25)
임상시험실시기관: 가톨릭관동대학교 국제성모병원(인천광역시 서구 심곡로 100 번길 25)
원광대학교 한방병원(전라북도 익산시 무왕로 895

3. 임상시험 의뢰자

보건복지부 (보건산업진흥원)

4. 임상시험의 목적 및 배경

1) 임상시험의 목적

본 연구는 비급성요통환자 (Non-acute low back pain)에 대한 단순방사선영상 변위진단에 따른 추나의학적 치료 적용의 효과(effectiveness) 및 안전성을 평가하는데 있음.

2) 임상시험의 배경 및 개요

□ 비급성 요통

○ 질환의 배경

- 요통은 전세계적으로 매우 흔한 근골격계 질환(일생동안 70~80%의 사람에게 영향을 미침)으로, 심각한 통증, 병가 증가, 사회적 비용 증가를 초래하는 주요 건강 문제임
- 특히, 비급성요통은 사회에 상당한 사회 경제적 문제를 야기하는 것으로 알려져 있음

○ 요통의 비침습적 통상치료

- 요통에 대한 비침습적 통상치료에 대한 최신 임상 진료 지침(Clinical Practice Guidelines, CPGs)은 약물 치료, 심리 치료, 물리 치료, 수기 치료 및 교육 치료를 포함한 다양한 방법을 제시하고 있으며, 이중 척추 수기요법(manual medicine) 치료는 만성, 아급성 및 급성 요통환자에게 낮은에서 중간정도의 근거로 권장되고 있음.

□ 추나요법

- 추나요법(Chuna Manipulative Therapy, CMT)은 해부학적 구조와 기능 간의 균형을 회복하기 위한 기술을 포함하며, 한의사에 의해 수행되는 한국식 수기 치료법임.
- 추나요법은 2019년 이후 근골격계질환에 대해 국내 건강보험 급여의 보장을 받고 있으며, 다양한 연구에 의해 유효성 및 안정성을 검증 받았음.
- 추나요법의 시술전 시행되는 진단은 크게 촉진에 의한 수기진단과 진단기기(영상의학적 진단기기, 체형분석 기기)를 활용한 진단으로 나뉘고 있으나, 각 진단방법들의 객관성, 진단자간-진단자내 진단재현성, 진단 결과에 따른 치료효과에 따른 결과 분석 등에 대한 결과는 매우 부족한 상황임.

□ 영상자료 기반 추나의학적 진단방법과 관련한 선행연구

○ 국내 임상연구

(1) 개요

- 현재까지 영상의학적 자료 기반 추나요법의 진단 방법은 **진단체계 구축, 추체기반 인공지능 진단프로그램 개발, 추나의학적 진단 일치도 및 AI 프로그램 적용 임상연구**로 시행되고 있음.
- 전문가 합의과정을 통해 도출된 표준 SOP 기반을 바탕으로 시행한 **추나의학적 영상 진단은 수기진단방법에**

비해 높은 진단자간 일치도를 나타내는 것으로 확인됨.

(2) 연구요약

<단순방사선 영상 기반 추나의학적 진단방법의 선행 연구 요약>

단계	분류	논문/연구 제목	연구개요
1	진단 체계 구축 (2011~2014)	척추변위 명명체계에 대한 문헌 고찰	- 추나의학적 척추변위 명명체계를 분류하여 제시함
		단순 방사선 영상검사를 통한 추나의학적 진단 방법	- 추나의학적 **영상진단 방법에 대한 기본 방법**을 제시
2	추체 마커 기반 AI 진단 프로그램 개발 (2019~)	추나 인공지능 프로그램 개발을 위한 추나 진단 인공지능 프로그램 개발을 위한 데이터베이스 구축 연구	1. **추체 기준 추나진단 기초 바이오마커 산정**<br>2. 영상자료 기반 **추나 진단 인공지능 프로그램의 개발** 연구
		합성곱 신경만을 이용하여 요추의 추나의학적 진단 기준점이 되는 추체 특징점 검출에 대한 연구	1. 요추부위 영상을 활용한 학습평가<br>2. **추체 특징점 자동 검출 알고리즘 및 다항 곡선 적합을 이용하여 전위 진단 알고리즘 개발**
3	임상연구 (2020~2022)	추나의학의 임상 진단 자료 확보 및 인공지능 진단 기술 개발을 위한 기반 구축 연구	1. **영상자료 기반 추나의학적 진단의 높은 유효성 확인**<br>2. **인공지능 프로그램의 활용 가능성 탐색**

○ **국외 연구 분석**

(1) 추체 변위 진단과 관련한 외국 선행 연구 분석

- 카이로프락틱, 정골의학 등에서 영상의학적 진단기기를 활용하는 것으로 알려져 있지만, 영상의학적 진단 도구를 활용한 진단 일치도 등에 관련한 연구는 부족한 실정임
- 수기의학적 척추 배열 진단과 관련하여 촉진을 통한 진단은 신경근골격의학 및 정골의학에서 동일한 전공 수련과정을 받은 전문가들 사이에서도 높은 오차를 나타냈다는 연구도 있고, 전후 비교평가에서 일치도가 높게 나왔다는 연구가 있는등 연구에 따라 상이한 내용을 보고한 경우가 많음. **이러한 연구별 상이한 차이가 발생한 이유는 촬영시 측정 자세의 표준화 시켰는지에 대한 여부**가 중요한 변수였을 것으로 사료됨.

(2) 추체 위치 파악과 관련한 인공지능 프로그램관련 연구

- 외국에서 시행된 추체 위치 파악과 관련한 인공지능 프로그램 개발 연구는 척추의 측만증 평가를 위한 평가에 치중한 경우가 많으며, **수기의학적 관점의 척추 배열을 평가하고 변위를 진단하는 형태의 연구는 부재함.**

○ **핵심 선행 연구 분석**

① **단순 방사선 영상 검사를 통한 추나의학적 진단 방법 (2014)**

- 추나의학적 수기적 진단의 한계를 보완하고, 진단의 객관성 및 재현성을 담보할 수 있는 새로운 형태의 진단 방법으로 단순방사선 영상을 활용한 진단방법을 제시
- 단순방사선 영상상 나타나는 골성 구조물을 포괄적으로 고려하여 추나의학적 변위 진단을 시행

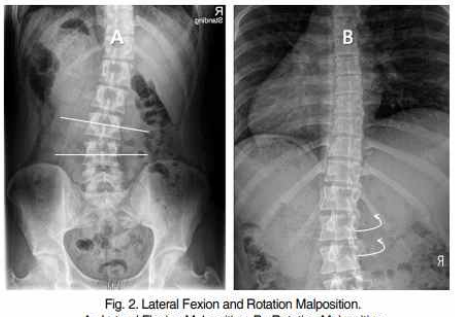

Fig. 2. Lateral Fexion and Rotation Malposition.
A : Lateral Flexion Malposition, B : Rotation Malposition

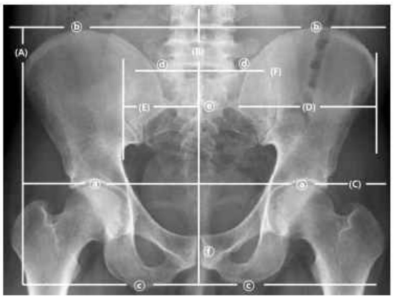

Fig. 5. X-ray Indicators of Sacroiliac Malposition.

<단순방사선 영상을 활용한 추나의학 진단방법>

(출처 : 이진현 외. 단순 방사선 영상 검사를 통한 추나의학적 진단방법. 척추신경추나의학회지. 2014;9(1):1-14.)

② **추나의학적 진단방법(촉진, 영상진단, AI 프로그램)들간의 진단 일치도 비교 연구 (IF-3.992)**

- **가톨릭관동대학교 국제성모병원에서 시행한 선행 연구**
- **추나의학의 진단방법의 일치도 비교를 통해 촉진을 통한 진단보다, X-ray 영상 자료를 활용한 추나의학적 진단이 진단자간 진단일치도가 높음**을 확인.
- **인공지능 프로그램을 활용할 경우 추나의학의 전문가의 진단 진단결과와 유사해지는 것 확인**

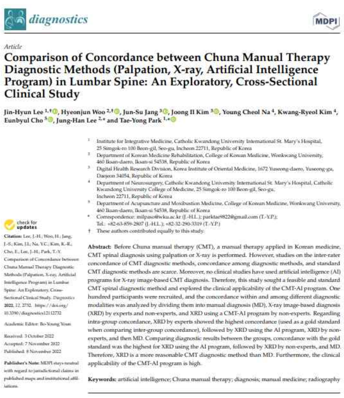

diagnostics MDPI

Article

Comparison of Concordance between Chuna Manual Therapy Diagnostic Methods (Palpation, X-ray, Artificial Intelligence Program) in Lumbar Spine: An Exploratory, Cross-Sectional Clinical Study

Jin-Hyun Lee [1,†], Hyeonjun Woo [2,†], Jun-Su Jang [3], Joong Il Kim [3], Young Cheol Na [4], Kwang-Ryeol Kim [4], Eunbyul Cho [5], Jung-Han Lee [2,*] and Tae-Yong Park [1,*]

1 Institute for Integrative Medicine, Catholic Kwandong University International St. Mary's Hospital, 25 Simgok-ro 100 Beon-gil, Seo-gu, Incheon 22711, Republic of Korea
2 Department of Korean Medicine Rehabilitation, College of Korean Medicine, Wonkwang University, 460 Iksan-daero, Iksan-si 54538, Republic of Korea
3 Digital Health Research Division, Korea Institute of Oriental Medicine, 1672 Yuseong-daero, Yuseong-gu, Daejeon 34054, Republic of Korea
4 Department of Neurosurgery, Catholic Kwandong University International St. Mary's Hospital, Catholic Kwandong University College of Medicine, 25 Simgok-ro 100 Beon-gil, Seo-gu, Incheon 22711, Republic of Korea
5 Department of Acupuncture and Moxibustion Medicine, College of Korean Medicine, Wonkwang University, 460 Iksan-daero, Iksan-si 54538, Republic of Korea
* Correspondence: milpaso@wku.ac.kr (J.-H.L.); parktae9822@gmail.com (T.-Y.P.); Tel.: +82-63-859-2807 (J.-H.L.); +82-32-290-3319 (T.-Y.P.)
† These authors contributed equally to this study.

check for updates

Citation: Lee, J.-H.; Woo, H.; Jang, J.-S.; Kim, J.I.; Na, Y.C.; Kim, K.-R.; Cho, E.; Lee, J.-H.; Park, T.-Y. Comparison of Concordance between Chuna Manual Therapy Diagnostic Methods (Palpation, X-ray, Artificial Intelligence Program) in Lumbar Spine: An Exploratory, Cross-Sectional Clinical Study. Diagnostics 2022, 12, 2732. https://doi.org/10.3390/diagnostics12112732

Academic Editor: Bo-Young Youn

Received: 3 October 2022
Accepted: 7 November 2022
Published: 8 November 2022

Publisher's Note: MDPI stays neutral with regard to jurisdictional claims in published maps and institutional affiliations.

Abstract: Before Chuna manual therapy (CMT), a manual therapy applied in Korean medicine, CMT spinal diagnosis using palpation or X-ray is performed. However, studies on the inter-rater concordance of CMT diagnostic methods, concordance among diagnostic methods, and standard CMT diagnostic methods are scarce. Moreover, no clinical studies have used artificial intelligence (AI) programs for X-ray image-based CMT diagnosis. Therefore, this study sought a feasible and standard CMT spinal diagnostic method and explored the clinical applicability of the CMT-AI program. One hundred participants were recruited, and the concordance within and among different diagnostic modalities was analyzed by dividing them into manual diagnosis (MD), X-ray image-based diagnosis (XRD) by experts and non-experts, and XRD using a CMT-AI program by non-experts. Regarding intra-group concordance, XRD by experts showed the highest concordance (used as a gold standard when comparing inter-group concordance), followed by XRD using the AI program, XRD by non-experts, and then MD. Comparing diagnostic results between the groups, concordance with the gold standard was the highest for XRD using the AI program, followed by XRD by non-experts, and MD. Therefore, XRD is a more reasonable CMT diagnostic method than MD. Furthermore, the clinical applicability of the CMT-AI program is high.

Keywords: artificial intelligence; Chuna manual therapy; diagnosis; manual medicine; radiography

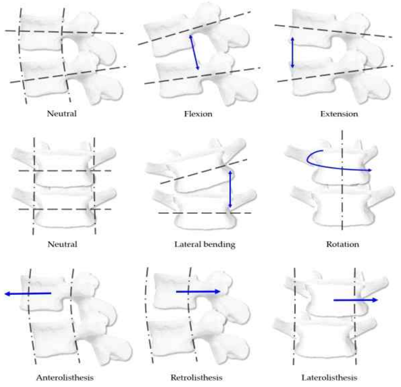

(추나의학적 변위진단 체계)

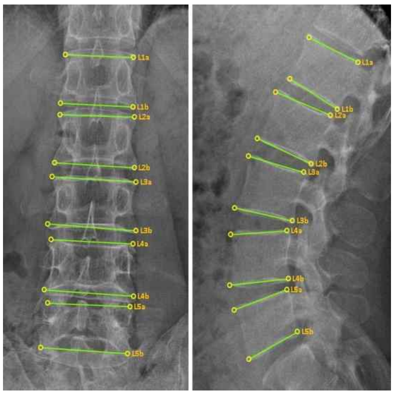

(AI 프로그램 특징점 도출)

<추나의학적 진단방법간 일치도 비교평가 연구>

(출처 : Jin-hyun Lee, et al. Comparison of Concordance between Chuna Manual Therapy Diagnostic Methods (Palpation, X-ray, Artificial Intelligence Program) in Lumbar Spine: An Exploratory, Cross-Sectional Clinical Study. Diagnostics (Basel). 2022 Nov 8;12(11):2732. doi: 10.3390/diagnostics12112732.)

□ 본 연구의 필요성 및 강점

○ **영상의학적 평가에 추나의학적 중재적용에 대한 유효성을 평가 연구의 필요**

- 현재까지 추나의학적 영상평가에 대한 선행연구는 개요 소개, 진단 지표점 설정, 진단자간의 진단 일치도 평가 등의 후향적 연구 형태로, **추나의학적 영상자료 기반 평가방법의 실제 임상에서의 유효성 평가에 대한 연구가 부재**하였음.
- 영상자료를 통한 **추나의학적 변위 평가에 따라 표준화된 추나의학적 치료방법을 적용함으로써, 임상적 유효성을 확인 가능한 임상연구 설계가 필요하다 사료됨**.

○ **유효성 및 안전성이 입증된 치료 중재의 적용**

- 실용적 연구디자인으로 선행 연구 및 가이드라인에 따른 통상치료군을 설정하고, 선행연구를 통해 안전성 및 유효성이 입증된 질환을 대상으로 추나요법을 적용함으로써 효율적 임상연구 진행이 가능함.

○ 한의 임상현장에서의 관련 근거 제공

- 영상의학적 자료를 기초로 바이오마커 추출을 통한 추나 영상진단은 추나 시술 전후의 효과를 비교하고 평가하는데 객관적 근거로 활용 가능하며, 추후 관련 추나 임상연구의 관련하여 보다 합리적이고 객관적 임상데이터 축적이 가능함.
- 합리적 추나진단을 위한 바이오마커 및 진단기술 개발은 추나 진단 급여화를 위한 선행 연구로 활용 가능

4. 시행되는 중재에 대한 설명

1) 임상시험에 활용되는 단순방사선영상 기반 추나의학적 진단 방법

(1) 영상 촬영 방법

- 요추 척추분절의 배열 평가를 위한 재현성을 검증한 선행연구에서 활용한 촬영 자세 이용
: 이 촬영 방법은 Chiropractic Biophysics(CBP) 표준화 절차에 따라 수행되며, 방사선 촬영의 재현성과 신뢰도를 높이기 위해 고안됨

	**AP view**<br><br>**Ap view 대상자 준비:**<br>대상자는 맨발로 서 있으며, 골반의 중심선이 방사선 장비의 중심선과 일치하도록 위치시킴.

<table>
<tr>
<td>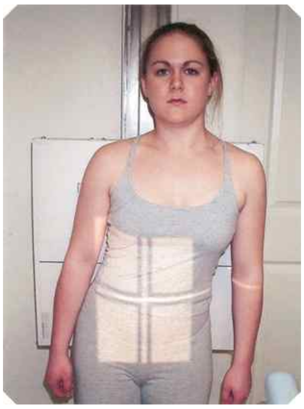</td>
<td>엉덩이를 방사선 촬영 장비에 평행하게 배치.

자세 설정:
골반의 정중면(mid-sagittal plane)이 방사선 중심선에 맞추어지고, 엉덩이가 촬영 장비의 중앙에 정렬. 대상자는 머리를 두 번 숙였다가 들어 올리며, 중립 자세(neutral position)를 취하도록 함.
눈을 감았다가 뜨고, 자연스럽게 전방을 응시합니다. 골반의 비정상적인 자세(예: 좌우 기울어짐)는 인위적으로 수정하지 않음.

광선의 위치와 거리:
방사선 광선은 L3 레벨 중심에 맞춰지고, 101.6cm(40인치)의 표준 촬영 거리에서 촬영.
촬영 각도는 일반적으로 수평으로 유지함.

특징
측정 변수
: 골반 중심축, 요추 곡률, 요추 분절의 기울기 등을 정확히 측정하기 위해 설계

중립 자세 유지
: 촬영 중 자세를 인위적으로 교정하지 않음으로써 자연스러운 체중 분배와 골반 정렬을 반영</td>
</tr>
<tr>
<td>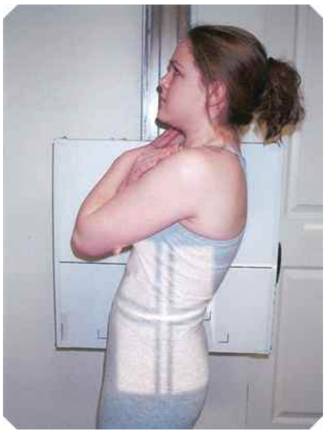</td>
<td>lateral view

대상자 준비
어깨와 골반은 방사선 장치와 수직.
대상자는 머리를 두 번 움직여 중립 자세를 설정.
팔은 가슴 앞에서 교차시켜 골반 또는 흉부 자세의 변화를 최소화.
비정상적인 요추 자세는 수정하지 않음

촬영 장치 준비
방사선 광선은 101.6cm(40인치) 거리에서 L4 레벨을 향함</td>
</tr>
</table>

(2) 추나의학적 척추변위 진단방법

- 수집된 요추부 단순방사선 영상의 dicom 파일을 바탕으로, 프로그램을 통해 선행연구에서 제시한 디지털마커를 활용하여 각 추체별 굴곡, 측굴, 회전 각도를 도출함.
- 디지털마커 레이블링 프로그램(Dicomlabel. Korea Institute of Oriental Medicine, Daejeon, Korea)을 사용하여 분절별 각도를 산출
- 디지털 마커 생성 방법

① lateral view

Lateral 요추에서는 각 요추 추체(vertebral body)의 윗면 전후 끝점(a1, a2)을 연결하여 상연선을, 아랫면 전후 끝점(b1, b2)을 연결하여 하연선을 생성한다(Figure 2-A). L5 의 척추 변위 진단을 위해서는 천추의 추체 기준점이 필요하므로 1 번 천추의 전후 끝점 2 개, 끝점을 이은 1 개의 선으로 이루어진 디지털 마커를 생성한다.

② AP view

AP 요추에서는 각 요추 추체의 윗면 좌우 끝점(c1, c2)을 연결하여 상연선을, 아랫면 좌우 끝점(d1, d2)을 연결하여 하연선을 생성한다. 또한, 양측 척추뿌리(pedicle)의 가장 내측점(e1, e2)을 추가로 생성한다(Figure 2-B). L5 의 척추 변위 진단을 위해 S1 의 위쪽면 좌우 끝점(f1, f2)과 끝점을 이은 선, sacral ala 의 가장 외측 끝점(g1, g2), S2 의 극돌기 중점(h)으로 이루어진 디지털 마커를 생성한다.

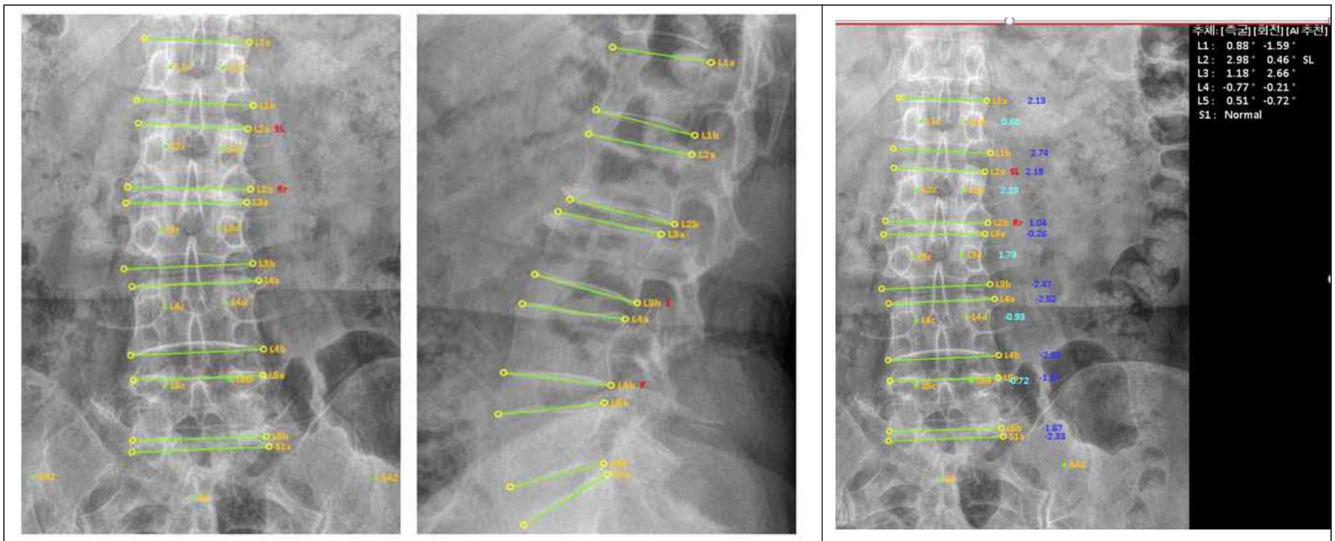

<디지털 마커 적용점(좌, 중) 및 프로그램을 통해 도출되는 각도정보 예(우)>

- 프로그램을 통해 도출된 각도를 바탕으로 변위 유무를 결정
  · 선행연구를 통해 도출된 아래와 같은 변위 진단을 위한 각도 문턱값을 기준으로, 문턱값 이상의 각도를 나타내는 분절을 추나의학적 변위가 있는 레벨로 선정 (예: L1 의 Flexion 각도가 3 도인 경우 굴곡변위로 진단).
  (단, **L5 의 회전변위의 경우 하위 요추 분절이 별도로 존재하지 않으므로, 양측 sacral ala 와 S2 tubercle 사이의 좌우 거리차를 비교하여 프로그램이 제시한 정상 범주에 들어온 경우에만 그 값을 활용하고 그렇지 않은 경우 증례기록서에 기록은 하되 진단 및 통계처리에서는 결측 값**으로 둔다)

	Vertebral body				
	L1	L2	L3	L4	L5
Flexion(°)	0.64	3.56	5.29	8.90	8.53
Extension(°)	10.46	14.95	17.61	20.76	24.47
Lateral bending(°)	2.21	2.10	1.92	2.06	2.31
Rotation(°)	9.49	5.19	4.59	5.87	7.13

2) **임상시험에 활용되는 치료 술기**

(1) 통상 치료 (공통 처치) : 물리치료 + 환자 교육

- 주 2 회간격으로 4 주간, 총 8 회의 치료 및 교육 시행

○ 물리치료

- 물리치료 : 경피적 전기신경자극치료 (Interferential Current Therapy, ICT)
  ① 통증이 가장 유발되는 부위를 기준으로 ICT 4 개의 패드를 서로 교차시켜 간섭전류가 통증 부위에 집중되도록 부착한다. 15 분 동안 4000Hz~4100Hz 중주파 교류전류를 통전 시 강도는 환자의 주관적 감각에 따라 통증을 느끼지 않을 정도로 시행, 주 2 회 간격으로 4주간, 총 8회의 치료를 시행.
  ② 사용되는 치료 기기명
  · 가톨릭관동대학교 국제성모병원 한의과 - 제조사: ㈜ 스트라텍/한국, 모델명: STT-570
  · 원광대학교 한방병원 - 제조사 : OG Giken Co/Japan, 모델명 : EF-160

○ 환자 운동 지도

- 환자의 운동과 관련하여서는 골반경사운동, 체중부하없이 허리돌리기, 무릎당기기, 상체들기 등의 운동을 방문시 15 분 정도 지도

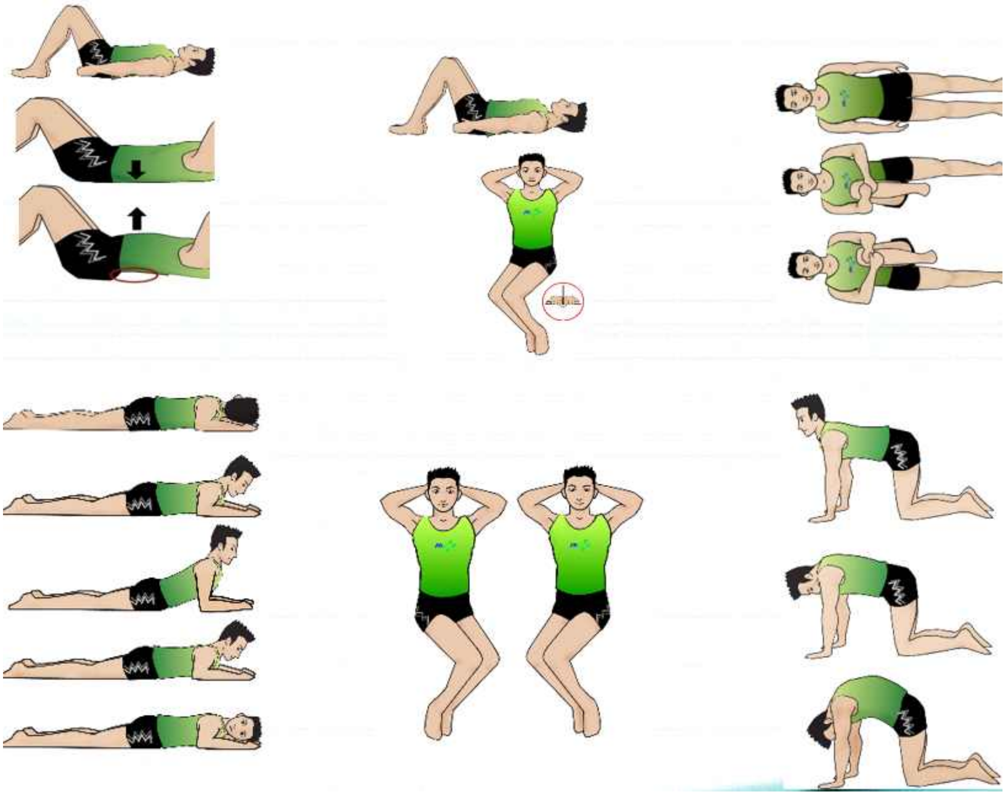

○ 환자교육

- 요통의 예방자세, 올바른 작업자세와 관련한 환자 교육자료 제공 및 설명

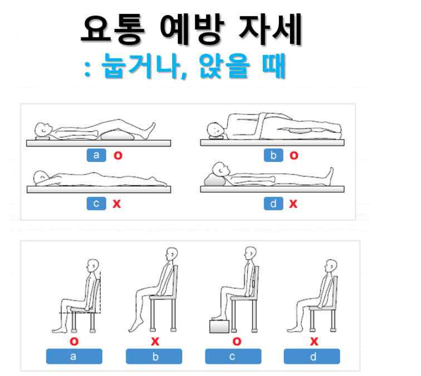

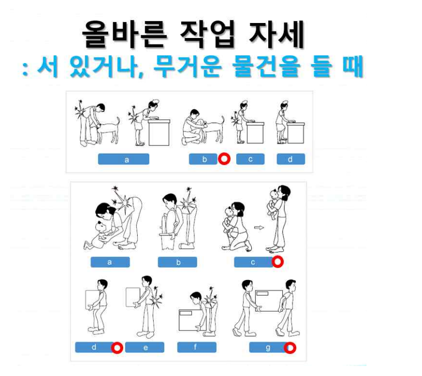

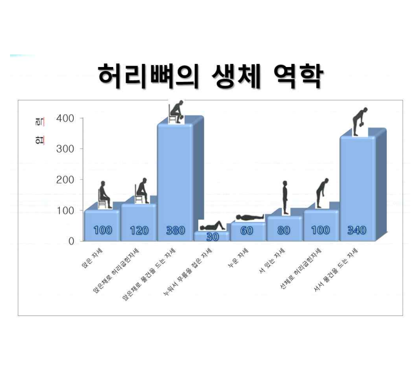

(2) 추나요법 : 추나치료군에만 적용

○ 단순방사선영상 기반 추나의학적 변위 진단결과에 따른 추나요법 시행

- 주 2 회간격으로 4 주간, 총 8 회 (1 회치료당 15 분 내외)의 치료를 시행.
- 추나의학적 변위 진단 결과에 따라 다음 항부터 제시되는 표준 작업지침을 활용하여 치료 시행
  · 추나의학적 변위 진단에 결과 분류 (단순변위, 복합변위)에 따라 해당레벨부위에 표준작업지침(SOP)에 따르는 추나의학적 치료 방법을 적용
  : 한 변위에 대한 복수 이상의 교정방법이 SOP 에 제시되어 경우 시술자의 판단에 따라 적절한 기법을 적용
- 임상의의 판단에 요추를 제외한 다른 부위(골반)에 대한 추가적인 추나요법이 필요하다고 사료되는 경우 관련 기법을 시행할수 있으며, 관련 내용을 증례기록서에 기록할 예정임 (단, 요추부에는 제시된 표준작업지침 이외의 추나 치료는 불가)

○ 시술전 초음파를 통한 정확한 시술 레벨 선정

- 단순방사선 영상에서 확인된 변위 레벨에 대한 정확한 시술 부위 접촉을 위해 초음파로 환자의 척추 레벨을 확인 후 예정된 시술 부위의 표면 위에 별도의 마킹을 시행
- longitudinal view 에서 spinous process 의 모양을 확인하여 S1 부터 순차적으로 상행하는 방법을 통해 레벨 확인

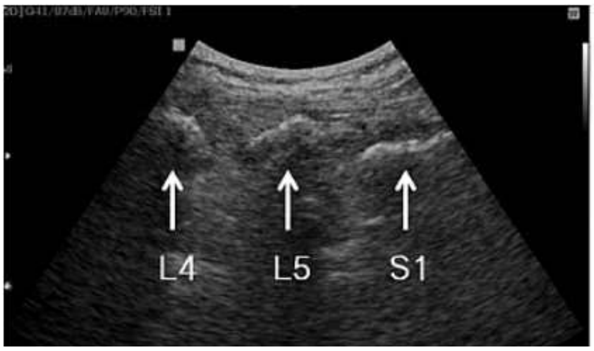

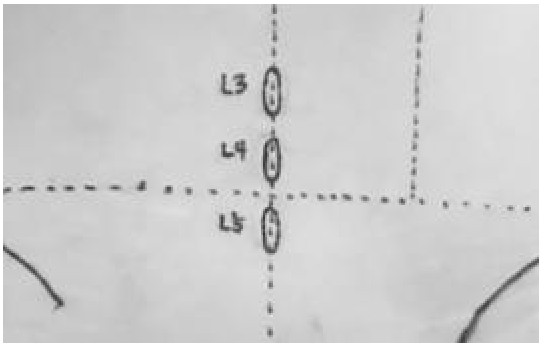

<초음파상 요추의 극돌기 모양 확인(좌) 및 체표 마킹(우)>

○ 연구에 활용하는 요추부 추나요법

① 단순변위에 따른 단순 추나요법

변위	환자 자세	의사 자세	접촉 지점 및 손의 형태	교정 방향
양측성 굴곡변위 좌위 요추 신전교정 기법	좌위, 양손으로 반대쪽 어깨를 감싸쥠	환자의 후면에 섬	주동수 장근부로 하위분절 극돌기 접촉<br>보조수로 환자의 양팔을 감싸잡아 상체를 고정<br>주동수로 후방에서 전방으로 병진 압력을 줘서 신전 제한장벽에 접근	환자는 체간을 구부리는 힘을 주고, 의사는 그에 저항함
양측성 신전변위 좌위 요추 굴곡교정 기법	좌위, 양손으로 반대쪽 어깨를 감싸쥐거나 다리 사이로 팔을 늘어뜨림	환자의 전측면에 펜싱 자세로 섬	술자의 한쪽 손으로 어깨를 고정하고 같은 쪽 겨드랑이 부위로 환자의 반대쪽 어깨를 고정<br>술자의 손가락을 변위가 있는 분절 사이의 극간에 접촉하여 움직임을 읽음<br>신전 변위가 있는 분절이 벌어질 때까지 환자의 체간을 구부림	환자는 체간을 펴려는 힘을 주고 의사는 이에 저항함
회전 변위 측와위 요추 신연기법	회전 방향을 아래로 한 측와위 후 아래다리의 오금에 위 다리 발을 위치시킴	환자의 전면에서 펜싱 자세를 취함	족방수의 손가락으로 환자의 위 다리 오금을 잡음<br>두방수의 손바닥은 환자 위 어깨의 전면에 접촉함	두방수는 두방, 족방수는 바닥쪽으로 누르며 회전, 신연시킴
측굴 변위 요추 굴곡신연 기법 중 측굴 기법	복와위 환자의 족부를 벨트로 고정	COX 테이블에서 골반대 측면에 섬	치료 분절의 위쪽 척추 극돌기를 두방수 장근부로 고정	보조수로 테이블 손잡이를 조작하여 추나테이블의 골반대를 좌측/우측으로 움직이게 함

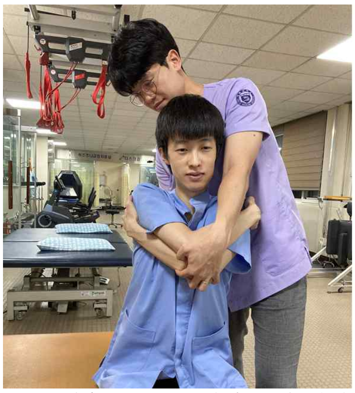
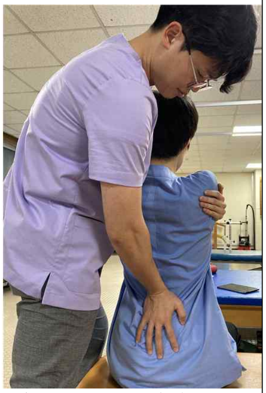

<양측성 굴곡변위에 대한 좌위 요추 신전교정 기법>

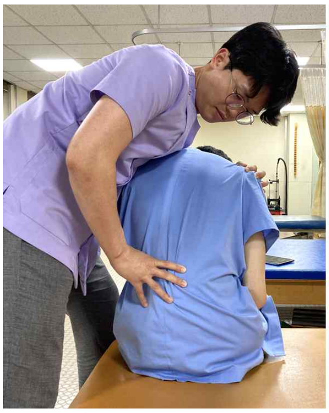
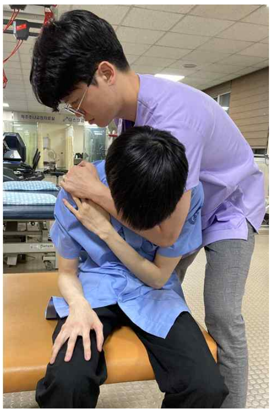

<양측성 신전변위에 대한 좌위 요추 굴곡교정 기법>

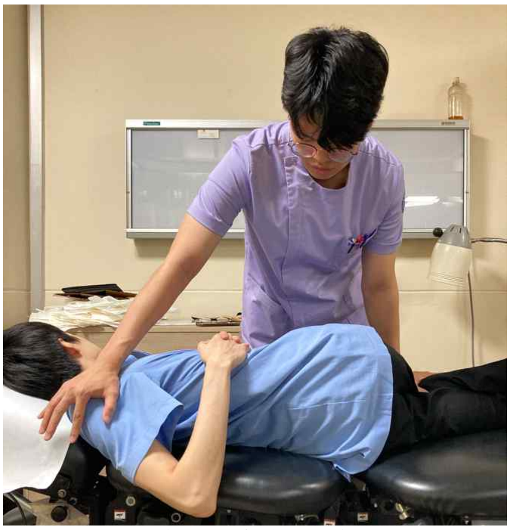

<회전 변위에 대한 측와위 요추 신연기법>

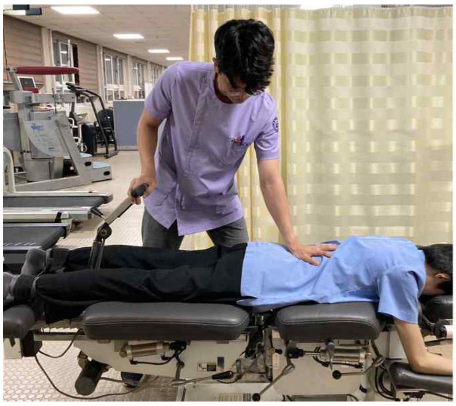
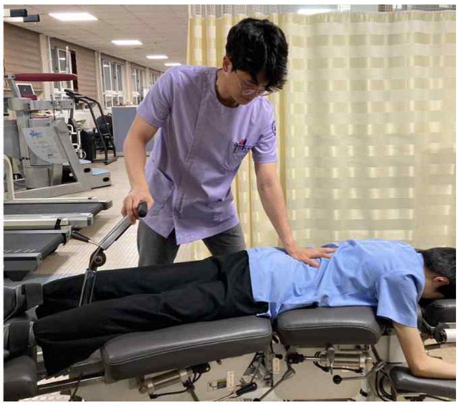

<측굴 변위에 대한 요추 굴곡신연기법 중 측굴기법>

② 단순 변위에 대한 전문추나요법

변위	환자 자세	의사 자세	접촉 지점	환자 접촉점	**손의 형태**	**교정 방향**
**양측성 굴곡 변위**<br>소어제부 극돌기 추법	머리 부위에 베개를 받치거나 테이블의 경추부분을 상승시킴<br>**어느 한쪽을 밑으로 한 측와위**<br>아래쪽 하지는 쭉 뻗고, 위쪽 하지는 아래쪽 하지의 오금에 걸쳐놓음	환자의 얼굴 방향을 향해 45도로 각도로 선 펜싱 자세<br>족방측 대퇴부로 환자의 대퇴부를 접촉하여 지지함	족방수의 소어제부로 접촉하며, 손가락은 척추와 각을 이루도록 함	**변위 분절의 상위 추골의 극돌기 상단**에 접촉	보조수를 환자의 위쪽 견관절에 접촉하여 환자의 손 위에 포개어 놓음	전하방으로 스러스트 시행<br>추골의 신전을 유도
**회전 변위**<br>소어제부 유두돌기 추법 (저항기법)	머리 부위에 베개를 받치거나 테이블의 경추부분을 상승시킴<br>**회전된 방향을 아래로 한 측와위**<br>아래쪽 하지는 쭉 뻗고, 위쪽 하지는 아래쪽 하지의 오금에 걸쳐놓음	환자의 얼굴 방향을 향해 45도로 각도로 선 펜싱 자세<br>족방측 대퇴부로 환자의 대퇴부를 접촉하여 지지하거나, 의사 대퇴부 사이에 환자의 하지를 끼워 지지함	족방수의 두상골부로 접촉하며 손가락은 척추에 평행하게 함	**변위 분절의 하위 추골의 유두돌기**에 접촉	보조수를 환자의 위쪽 견관절에 접촉하여 환자의 손 위에 포개어 놓음	전상방으로 스러스트 시행.<br>후관절에서의 회전과 신연을 유도.
**측굴 변위**<br>소어제부 유두돌기 추법 (opened wedge side로 접근)	머리 부위에 베개를 받치거나 테이블의 경추부분을 상승시킴<br>**측굴된 방향을 아래로 한 측와위**. 체간부 테이블 아래를 내려 측굴을 만들어 줌<br>아래쪽 하지는 쭉 뻗고, 위쪽 하지는 아래쪽 하지의 오금에 걸쳐놓음	환자의 얼굴 방향을 향해 45도로 각도로 선 펜싱 자세<br>족방측 대퇴부로 환자의 대퇴부를 접촉하여 지지하거나, 의사 대퇴부 사이에 환자의 하지를 끼워 지지함	족방수의 두상골부로 접촉하며 손가락은 척추에 평행하게 함	**변위 분절의 하위 추골의 유두돌기**에 접촉	보조수를 환자의 위쪽 견관절에 접촉하여 환자의 손 위에 포개어 놓음	전방, 내방, 상방으로 스러스트 시행.<br>Opened wedge를 닫아줌
**측굴 변위**<br>소어제부 유두돌기 추법 (closed wedge side로 접근)	머리 부위에 베개를 받치거나 테이블의 경추부분을 상승시킴<br>**측굴된 반대 방향을 아래로 한 측와위**. 허리 아래 부분에 쿠션을 대어 줌<br>아래쪽 하지는 쭉 뻗고, 위쪽 하지는 아래쪽 하지의 오금에 걸쳐놓음	환자의 얼굴 방향을 향해 45도로 각도로 선 펜싱 자세<br>족방측 대퇴부로 환자의 대퇴부를 접촉하여 지지하거나, 의사 대퇴부 사이에 환자의 하지를 끼워 지지함	족방수의 두상골부로 접촉하며 손가락은 척추에 평행하게 함.	**변위 분절의 상위 추골의 유두돌기**에 접촉	보조수를 환자의 위쪽 견관절에 접촉하여 환자의 손 위에 포개어 놓음	전방, 상방으로 스러스트 시행<br>Closed wedge를 열어줌

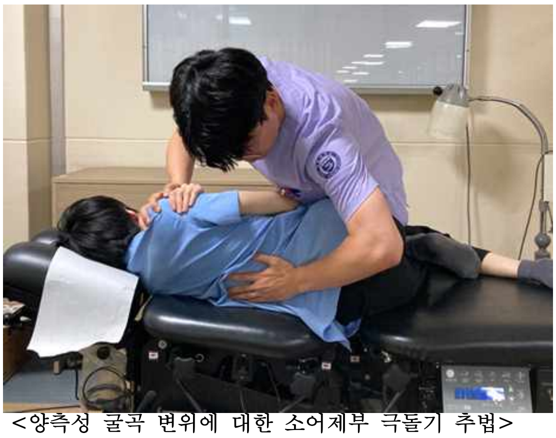

<양측성 굴곡 변위에 대한 소어제부 극돌기 추법>

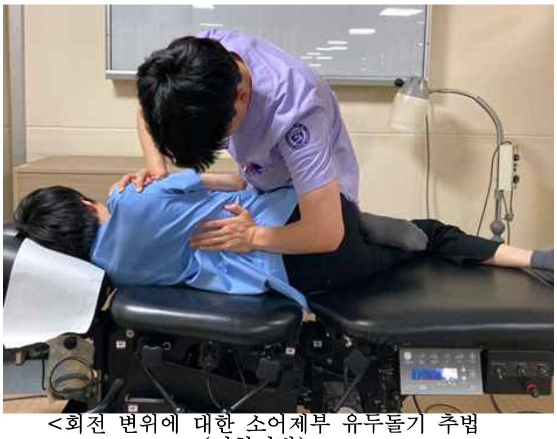

<회전 변위에 대한 소어제부 유두돌기 추법 (저항기법)>

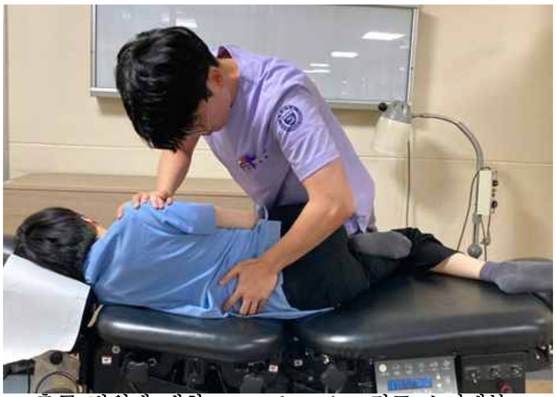

<측굴 변위에 대한 opened wedge 접근 소어제부 유두돌기 추법>

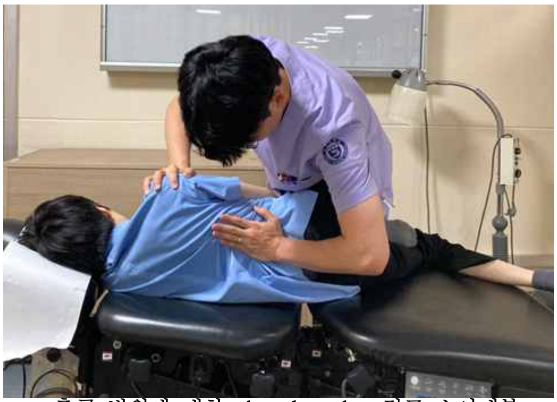

<측굴 변위에 대한 closed wedge 접근 소어제부 유두돌기 추법>

③ 복합변위에 대한 단순 추나요법

변위	환자 자세	의사 자세	접촉 지점 및 손의 형태	**교정방향**
**신전 회전 동측측굴 변위** 측와위 요추 교정기법	회전 방향을 아래로 한 측와위	환자의 전면에서 펜싱 자세를 취함	환자의 체간을 굴곡시켜 변위 분절의 굴곡을 만듦<br>술자의 전완으로 어깨를 뒤로 밀어 회전을 만들며 변위 부위의 움직임을 확인함<br>환자의 아래 다리 발목 부위를 통해 두 다리를 위로 들어 측굴을 만듦	환자는 다리를 치료대 방향으로 내리는 힘을 주며 술자는 그에 저항함
**굴곡 회전 동측측굴 변위** 측와위 요추 교정기법	회전 방향을 아래로 한 측와위	환자의 전면에서 펜싱 자세를 취함	환자의 체간을 신전시켜 변위 분절의 신전을 만듦<br>술자의 전완으로 어깨를 뒤로 밀어 회전을 만들며 변위 부위의 움직임을 확인하고, 환자는 치료대 모서리를 잡아 회전을 유지함<br>환자의 위 다리를 전체적으로 감싸 천장으로 들어올려 측굴을 만듦	환자는 다리를 치료대 방향으로 내리는 힘을 주며 술자는 그에 저항함
**측굴 대측회전 변위** 좌위 요추 교정기법 (좌측굴 우회전 변위를 예시로 설명)	좌위, 왼손으로 우측 어깨를 잡음	환자의 뒤에서서 좌측 팔로 환자의 좌측 액와를 지나 우측 어깨를 잡고 상부 체간을 조절	시술자의 우측 엄지로 우측으로 볼록한 요추 만곡의 정점을 전내측 방향으로 누름	우측 엄지로 국소화된 만곡의 첨부를 좌측으로 병진시키면서 동시에 상부 체간을 조절하여 우측굴 좌회전을 유도<br>환자는 3-5초간 우측굴 힘에 저항

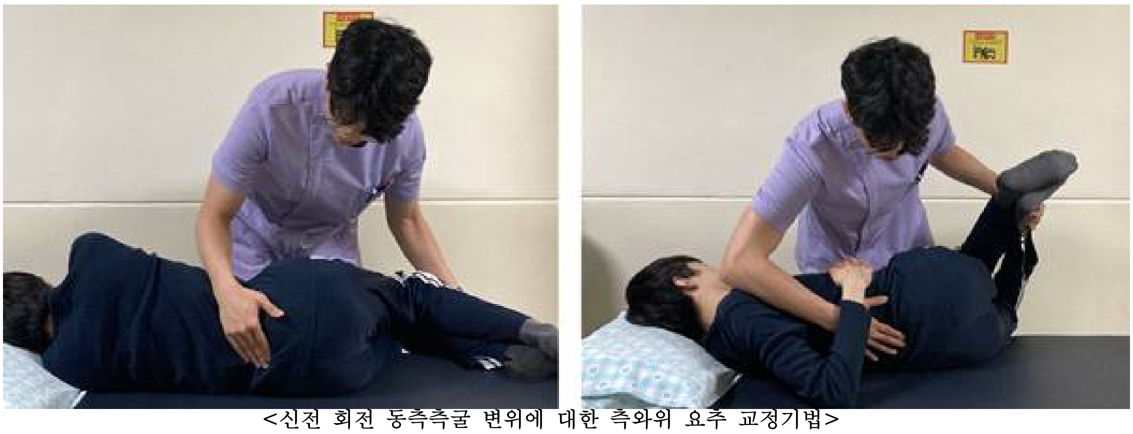

<신전 회전 동측측굴 변위에 대한 측와위 요추 교정기법>

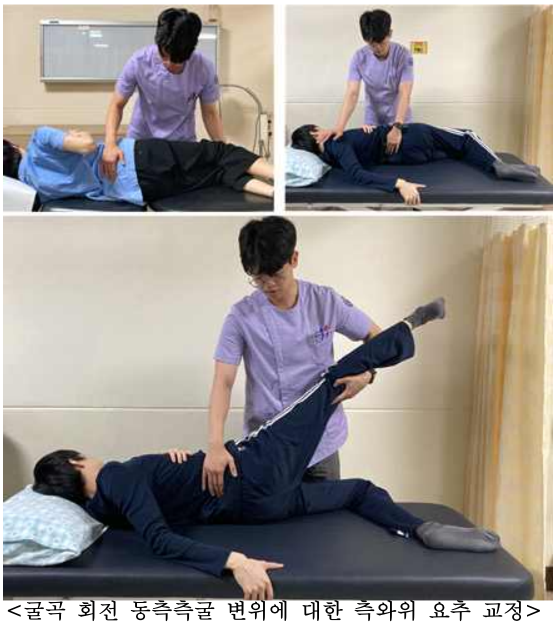

<굴곡 회전 동측측굴 변위에 대한 측와위 요추 교정>

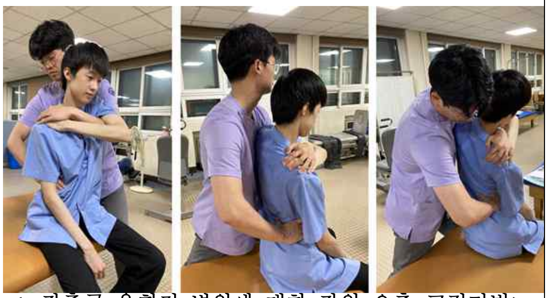

<. 좌측굴 우회전 변위에 대한 좌위 요추 교정기법>

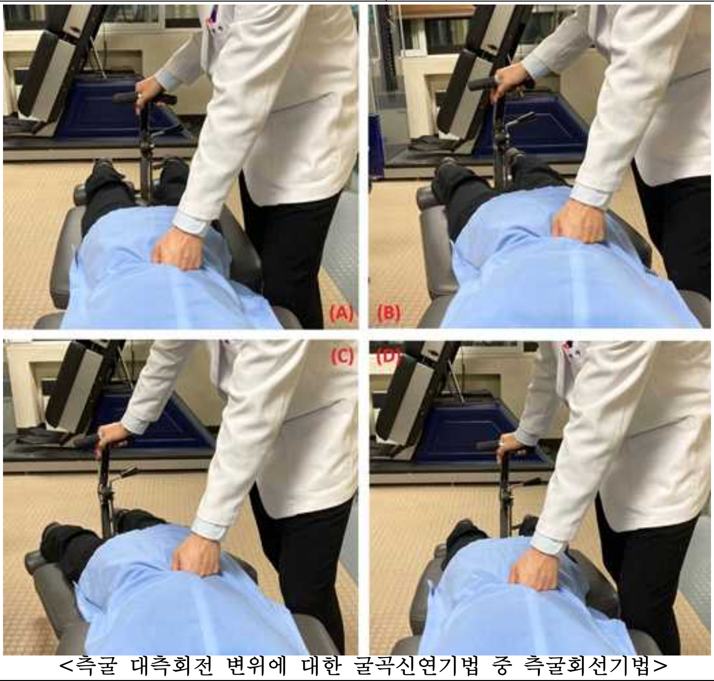

<측굴 대측회전 변위에 대한 굴곡신연기법 중 측굴회선기법>

④ 복합 변위에 대한 전문 추나요법

변위	환자 자세	의사 자세	접촉 지점	손의 형태	교정 방향
**신전 회전 동측측굴 변위** 측와위 요추 교정기법	회전 방향을 아래로 한 측와위	환자의 전면에서 펜싱 자세를 취함	주동수(족방수) 전완부를 장골에 접촉 보조수(두방수) 전완부를 액와부에 접촉	교정하려는 분절을 촉지하여 움직임을 확인함	보조수로 체간을 후방회전시키며 주동수로 장골을 전상방으로 당기며 체중을 이용한 순간 교정 시행
**굴곡 회전 동측측굴 변위** 측와위 요추 교정기법	회전 방향을 아래로 한 측와위	환자의 전면에서 펜싱 자세를 취함	주동수(족방수) 전완부를 장골에 접촉 보조수(두방수) 전완부를 액와부에 접촉	교정하려는 분절을 촉지하여 움직임을 확인함	보조수로 체간을 후방회전시키며 주동수로 장골을 전상방으로 당기며 체중을 이용한 순간 교정 시행
**측굴 대측회전 변위** 측와위 요추 교정기법	회전 방향을 아래로 한 측와위	환자의 전면에서 펜싱 자세를 취함	주동수(족방수) 전완부를 장골에 접촉 보조수(두방수) 전완부를 액와부에 접촉	교정하려는 분절군을 촉지하여 움직임을 확인함	보조수로 체간을 후방회전시키며 주동수로 장골을 전방으로 당기며 체중을 이용한 순간 교정 시행

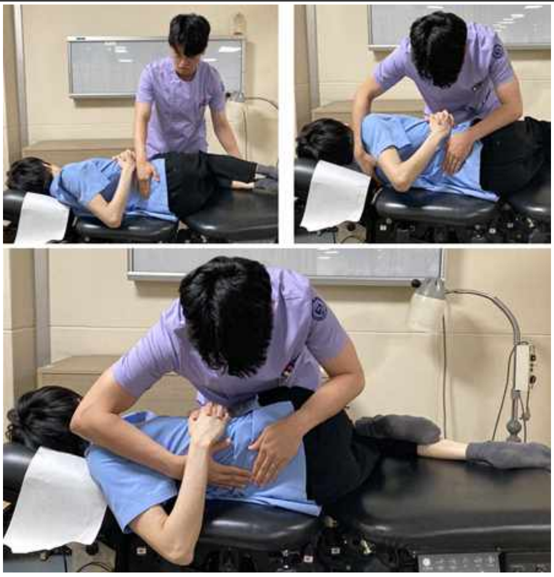

<신전 회전 동측측굴 변위에 대한 측와위 요추 교정기법>

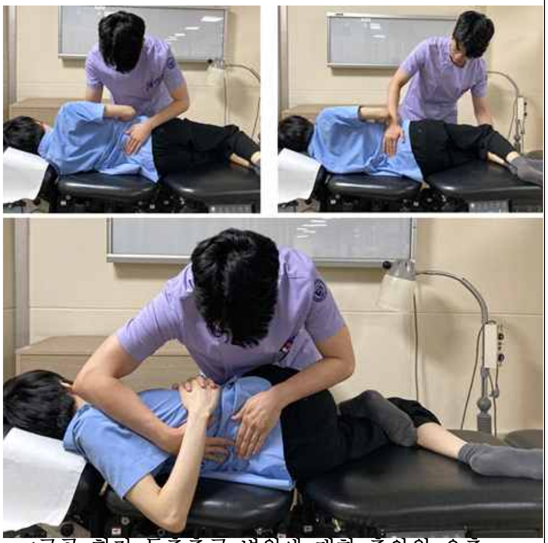

<굴곡 회전 동측측굴 변위에 대한 측와위 요추 교정기법>

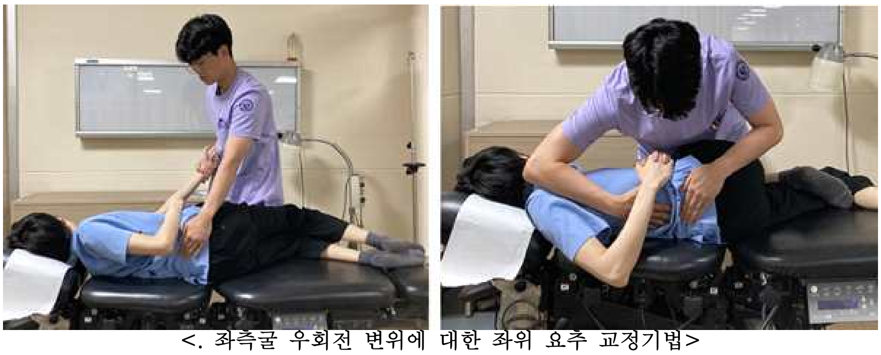

<. 좌측굴 우회전 변위에 대한 좌위 요추 교정기법>

5. 대상자의 포함기준, 제외기준, 목표한 대상자의 수 및 그 근거

본 임상시험의 대상자는 발병 후 3주이상 경과한 비급성 요통환자로, 아래와 같은 포함/제외기준에 따라 연구에 선정된 대상자를 대상으로 함.

1) 포함기준(Inclusion Criteria)

① 연령: 만 19세 이상 만 70세 이하
② 비급성(발병 후 3주이상 경과) 요통으로 최근 일주일간의 평균 통증 강도 Numeric Rating Scale (NRS) ≥4 인 자
③ 단순방사선 영상을 통해 추나의학적 변위가 있는 것으로 확인된 환자
④ 임상시험 참여에 자발적으로 동의하고 동의서에 서명한 자

2) 제외기준(Exclusion Criteria)

① 요통의 원인이 될 수 있는 심각한 특정 질병을 진단 받은 경우 (종양의 척추 전이, 급성 골절 및 척추탈구, 중증도 이상의 척추의 측만증 등)
② 요추의 구조적 이상 (sacralization, lumbarization), 골다공증의 기왕력이 있거나, 골절이 의심되는 경우
③ 요추 수술 후 3개월 이내의 환자인 경우
④ 치료효과나 결과의 해석을 방해할 수 있는 다른 만성적인 질환이 있는 경우 (만성 신부전)
⑤ 진행성의 신경학적 결손이 있거나, 마미증후군 등의 심각한 신경학적 증상이 동반된 경우
⑥ 요추 수술로 내부 고정 및 안정 장치가 있는 경우
⑦ ICT 치료의 금기증인 경우(암성 동통, 심박조정기 착용환자 등)
⑧ 현재 스테로이드제제, 면역억제제, 정신질환 약물 또는 연구 결과에 영향을 줄 수 있는 기타 약물을 복용하고 있는 경우
⑨ 최근 1주일 이내에 추나치료를 받았거나, 강한 마약성 진통제 및 마취제 등의 약물을 투여받은 경우 또는 침, 주사 등 침습적 치료를 받은 경우
⑩ 최근 2개월이내에 요추부에 신경차단술 시술을 받은 경우
⑪ 임신중이거나 모유 수유중인 자 또는 임신을 계획 중인 경우
⑫ 시술기간 (4주) 동안 담당의사의 지시 없이 연구 결과 판정에 영향을 미칠 수 있는 수술이나 시술, 약물 등의 치료를 받을 예정인 경우
⑬ 기타 연구담당자가 판단하기에 임상시험 참여에 부적절 할 것으로 판단한 자

3) 대상자의 수 및 그 근거

(1) 1차 예비임상 연구

- **대상자수 46명** : 다기관 모집
  · 가톨릭관동대학교 국제성모병원 : 12명
  · 원광대학교 한방병원 : 34명

군별	대상자 수	예정된 시술 중재
I	23명	통상치료 (ICT+운동지도) + 추나요법
II	23명	통상치료 (ICT+운동지도)

(2) 예비임상 연구의 대상자수 산출근거

□ 선행연구 분석 결과 - 본 연구의 표본 크기는 선행 유사연구에서 보고된 결과를 바탕으로 Cohen의 효과 크기를 기반으로 산출하였다. 선행연구에서 추나요법+통상치료 그룹의 요통 NRS 점수는 3.02±1.72였으며, 통상치료 그룹은 1.36±1.75였다($p<0.001$). 이 데이터를 바탕으로 효과 크기(Cohen's d)를 계산한 결과, d=0.96이었다. 양측 검정(two-tailed test), 유의수준(α) 0.05, 검정력(power) 80%를 적용하여 다음 공식을 사용하였다:

$$n = 2 \times [(Z_{\alpha/2} + Z_{\beta})^2/d^2]$$

여기서 $Z_{\alpha/2}$ 는 유의수준 0.05 에 대한 양측 검정 Z 값인 1.96 이고, Z_{β} 는 검정력 80%에 대한 Z 값인 0.84 이다. 계산 결과, 각 그룹당 필요한 최소 표본 수는 18 명으로 산출되었다. 연구 과정에서 예상되는 20%의 탈락률을 고려하여, 최종적으로 각 그룹당 23 명, 총 46 명의 대상자를 모집하기로 하였다.

6. 시험기간

IRB 승인일 – 2026.12.31

7. 시험방법

- 총 대상자수: 23 명 X 2 중재군 = 총 46 명

군별	대상자 수	예정된 시술 중재
I	23명	통상치료 (ICT+운동+생활습관지도) + 추나요법
II	23명	통상치료 (ICT+운동+생활습관지도)

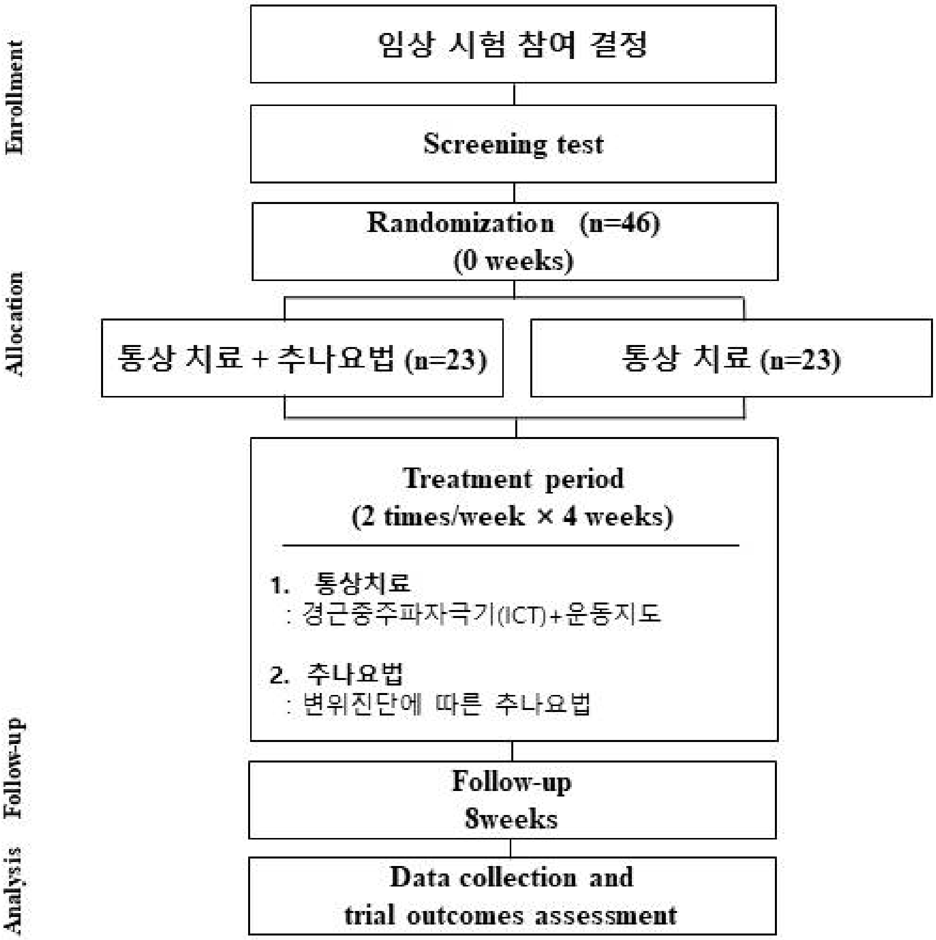

<연구흐름도>

1) 대상자번호 부여 (Randomization)

임상시험 참여를 서면으로 동의한 자를 대상으로, 스크리닝 시 외래 방문 순서에 따라 가톨릭관동대학교 국제성모병원 CK -S001, 원광대학교 한방병원 WK-S001 순서로 스크리닝 번호를 부여한다. 스크리닝 테스트 후 선정/제외기준에 따라 최종 대상자로 선정한다. 대상자 선정 이후 연구자 중 연구 결과에 영향을 끼칠만한 위험이 없으며, 연구 결과 분석에도 영향을 주지 않는 1 인 (시술 및 평가에 관여하지 않음)을 Unblind 하여 통계학자에게 의뢰하여 작성된 블록무작위배정리스트를 바탕으로 투과되지 않는 봉투에 배정될 군이 들어있는 무작위배정봉투를 대상자의 배정 순서에 따라 개봉하여 가톨릭관동대학교 국제성모병원 CK-R001, 원광대학교 한방병원 WK-R001 형식으로 대상자 번호를 배정한다. 한 명의 대상자에게 두 개 이상의 무작위 배정번호를 부여할 수 없으며, 한 개의 무작위배정번호가 오직 한 대상자에게만 부여된다. 각 대상자에게 부여된 스크리닝 번호, 무작위 배정번호, 이니셜은 임상시험이 끝날 때까지 대상자를 인식하는 대상자식별코드(subject identification code)로 사용된다. 무작위 배정목록은 통상 치료군+추나요법(I 군)과 통상치료 치료군(II 군) 1:1 의 비율로 배정될 것이며, 무작위배정표는 독립된 전문 통계학자에 의해 생성되며, 무작위배정봉투도 연구에 관여되지 않은 제 3 자가 제작한다.

2) 눈가림 및 눈가림 해제

본 임상시험에서는 치료 중재 및 연구 설계의 특성상 임상시험 담당자와 임상시험 대상자가 각 군별로 눈가림(blind)을 하는 것은 불가능하다. 대신 평가자는 눈가림을 유지함으로써 대상자를 평가하는 데 있어 bias 를 방지할 계획이다. 임상연구 담당자와 대상자가 눈가림을 시행치 않으므로, 별도의 눈가림 해제 절차를 시행하지 않는다.

3) 시술 방법

(참고) 중재에 대한 세부 정보는 앞서 언급한 **6-2) 임상시험에 활용되는 치료 술기** 항목 참조.

○ 통상치료 (경근 중주파 치료 + 운동 및 생활습관 교정)

(1) 경근 중주파 치료(간섭파 전류치료; Interferential Current Therapy, ICT)

- 2024년도 한국보건의료원(NECA)에서 발행된 의료기술재평가보고서에서 요통에 효과 및 안정성이 있다고 보고한 ICT 를 통상치료로 활용
- 연구에 enrollment 된 이후 통증부위에 2 회/주 간격으로 4 주간, 회당 15 분, 총 8 회를 시행

(2) 운동 및 생활습관 교정

- 내원시 골반경사운동, 체중부하없이 허리돌리기, 무릎당기기, 상체들기 등의 운동을 15 분 정도 지도
- 요통의 예방자세, 올바른 작업자세와 관련한 환자 교육자료 제공 및 설명

○ 추나요법

(1) 시술 부위 및 방법

· Screening 방문시 확인된 요추의 변위 위치를 확인 후 해당 척추분절에 변위유형에 따른 SOP에 제시된 표준 추나기법을 적용 (필수 추나)

· 한 변위에 대한 복수 이상의 교정방법이 SOP 에 제시되어 경우 시술자의 판단에 따라 적절한 기법을 적용

· 시술자의 판단에 요추를 제외한 다른 부위(골반)에 대한 추가적인 추나요법이 필요하다고 사료되는 경우 관련 기법을 시행할 수 있으며, 관련 내용을 증례기록서에 기록할 예정임 (단, 요추부에는 제시된 표준작업지침 이외의 추나 치료는 불가)

(2) 시술 시간 및 횟수

- 2 회/주 간격으로 4 주간, 총 8 회 (1 회치료당 15 분 내외)의 치료를 시행.

4) 병용 허용 의약품 및 주의사항

- 본 임상시험은 임상시험 기간 중에는 제공되는 중재를 제외하고, 일체의 다른 의약품을 투여하지 않는 것을 원칙으로 한다. 단, 본 임상시험은 실용적 임상시험 형태로 이상반응의 처치, 환자의 통증 관리 등을 위해 구제약물의 투약이 필요한 경우에는 시험책임자의 판단에 따라 투여할 수 있다. 투여된 의약품이 본 임상시험의 약력학적 평가 및 안전성 평가에 영향을 줄 수 있다고 예상되는 경우, 해당대상자는 탈락하게 된다(사용가능한 구제약물은 '13. 구제약물' 항을 참조). 투여된 모든 의약품과 투여사유는 반드시 근거문서와 증례기록서에 기재하고 시험책임자/담당자가 서명한다. 이외의 의약품 투여는 대상자가 반드시 연구진과 상의하여 결정할 수 있도록 한다.

8. 관찰 및 검사 항목

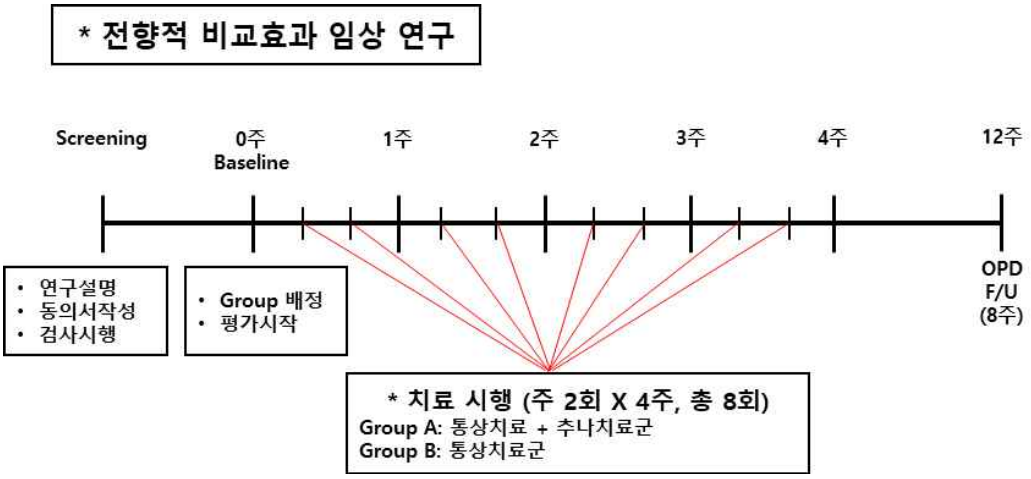

1) Screening Visit(visit 1, day –7 ~ 0)

대상자의 적합성 스크리닝 검사는 서면동의서를 작성한 대상자에 한해 실시된다. 검사 결과 다음 항목에 의해 임상적으로 유의한 이상이 있는 대상자는 제외한다.
(단 screening visit 에서 연구 등록이 확정된 경우 해당일에 baseline test 을 동시에 수행할 수 있음)

(1) 인구학적 조사 : 성별, 나이, 신장, 체중, BMI, 흡연력, 음주력
(2) 병력 조사 : 요통의 기간, 약물복용, 과거 병력
(3) 신체검사, 활력징후
일반상태, 영양상태, 피부/점막, 눈, 이비인후계, 갑상선, 폐, 심장/순환계, 복부, 신장/비뇨생식계, 신경/정신계, 척추/사지/종양, 말초순환, 림프계 등에 대한 문진 및 신체검사를 시행한다.
혈압(좌위), 맥박수, 체온을 측정한다(반드시 급격한 체위 변동 없이 5 분 이상 좌위를 유지한 상태에서 혈압과 맥박수를 측정).
(4) 영상검사 및 추나 변위 유무 파악
① 앞서 언급한 6-1.임상시험에 활용되는 단순방사선영상 기반 추나의학적 진단 방법 항목의 Chiropractic Biophysics 표준화 절차에 따라 L-spine AP & Lateral standing view 영상을 촬영.
② 영상 DICOM 파일을 바탕으로 추나의학적 변위 유무 및 유형을 확인
(5) 선정/제외기준 확인 : NRS 검사, 기존 병력, 치료력 등을 확인하여 선정/제외 기준을

평가 한다.

2) Baseline Visit(visit 2, day 0) - Visit 1 과 같은날 시행 가능

Screening test 를 통과하여 임상시험에 참여하게 된 환자의 경우블록 무작위배정을 실시하여 대상자 번호를 부여한 뒤 다음과 같은 평가를 진행한다.

(1) 환자의 주관적 증상, 기능 제한, 삶의 질에 대한 설문평가
· NRS (요통 및 하지통으로 구분지어 측정)
· Oswestry disability index (ODI)
· European Quality of Life 5 Dimension (EQ-5D)-5L,
· Roland Morris Disability Questionnaire (RMDQ)]

(2) 이학적 평가
· 요추의 관절가동범위 검사 (ROM)
: Flexoin, Extension, lateral flexion (Rt. & Lt.), Rotation (Rt. & Lt.)

(3) 활력징후
- 혈압(좌위), 맥박수, 체온을 측정하며, 측정 전에는 반듯이 급격한 체위 변동 없이 5 분 이상 좌위를 유지한다.

(4) 영상검사
- Visit 2 에서의 영상평가는 별도로 시행하지 않고 visit 1 의 screening 검사로 대체함

3) 치료방문 (통상치료+추나요법군, 통상치료군 동일 방문 - 총 8 회)

(1) 통상치료+추나요법군 (I 군, visit 3, 4, 5, 6, 7, 8, 9, 10)
- Baseline visit 직후부터 2 회/주 간격으로 4 주간, 총 8 회 시행

(2) 통상치료군 (II 군, visit 3, 4, 5, 6, 7, 8, 9, 10)
- Baseline visit 직후부터 2 회/주 간격으로 4 주간, 총 8 회 시행

(3) 매 시술전 활력증후를 측정한다.
- 혈압(좌위), 맥박수, 체온을 측정하며, 측정 전에는 반듯이 급격한 체위 변동 없이 5 분 이상 좌위를 유지한다.

(4) 시술전 이상반응을 확인한다.
- 이전 방문 이후 발생한 이상반응과 타각적 이상반응에 대해서 확인한다.

4) 평가 방문 (I 군, II 군 동일 - visit 2, 10, 11)

(1) 스크리닝을 통과한 모든 대상자는 baseline visit, 치료시행 종료 후(baseline 이후 4 주) 모든 시술이 종결된 8 주 (baseline 후 12 주) 후 외래를 방문하여 아래 검사를 실시한다.

(2) 활력징후

(3) 환자의 주관적 증상 및 기능 평가
- 1 차 유효성 평가 및 2 차 유효성 평가를 시행
: 환자의 통증 강도 및 기능 평가 설문지 활용 NRS, ODI, EQ-5D-5L, RMDQ

(4) 이학적평가
- 요추 ROM : Flexoin, Extension, lateral flexion (Rt. & Lt.), Rotation (Rt. & Lt.)

(5) 영상평가
- 표준화 절차에 따른 L-spine AP & Lateral standing view 영상을 촬영.
- 촬영된 영상을 바탕으로, dicom labeling 프로그램을 통해 추나의학적 변위 유형 진단을 위한 추체별 상대적 각도(flexion, extension, lateral bending, rotation) 및 추나의학적 변위 진단 결과 값을 비교.
(참고: visit 2에서의 영상평가는 별도로 시행하지 않고 visit 1의 screening 검사로 대체함)

(6) 탐색적 유효성 평가
- Treatment period 후 추가적인 의과의 침습적 시술[주사치료(신경차단술) 고주파

열응고술 등] 시행 여부, 강한마약성 진통제 사용여부(strong opioid), 허리 수술 여부에 대한 조사를 시행

(7) 이상반응 모니터링

- 평가방문 이외에도 치료기간 중 수시로 문진 또는 질문 등을 통하여 확인한다.

5) 조기 종료 방문 (Early Discontinuation Visit)

임상시험참여 후 중재를 1회 이상 실시한 이후 조기 종료 기준에 부합하여 임상시험이 종료된 환자는 마지막 시술 8주 뒤 시험기관을 일정 외 방문하여 평가방문을 시행한다 (단, 평가방문을 원칙으로 하되, 환자의 요청이 있는 경우 신체검사, 활력징후, 이학적평가, 영상평가를 제외한 최대한의 항목을 대해서는 전화 상담으로 대체할 수 있음). 중지/탈락기준에 의거하여 시험을 조기에 중지하거나 시험대상자가 탈락하는 경우에는 별도의 추적관찰 평가를 시행치 않는다.

9. 중지/탈락 및 임상시험 조기 종료 기준

○ 중지/탈락 기준

1) 대상자가 중재방법 유효성을 평가하는데 영향을 줄 것으로 예상되는 아래와 같은 의약품은 스크리닝 이후부터 임상시험 완료시까지 복용할 수 없다.

① 강한 마약성 진통제(strong opioid), 임상시험 담당의가 투약과는 별도로 투여된 국소 마취제 및 스테로이드제제
단, 임상의의 판단을 기초로 필요에 의해 처방되는 소염진통제, 근이완제 등은 투약가능 (11. 구제약물 참조)

② 기타 임상시험담당의가 판단하기에 환자에게 위험을 초래하거나 연구의 평가결과에 비뚤림을 유발할 수 있을 것이라 판단되는 약물

2) 대상자가 임상시험 중 임상시험 중재 시술의 중단을 요구하거나, 시험참여 동의를 철회하는 경우

3) 중대한 이상반응이 발생하여 시험자가 시험을 계속할 수 없다고 판단하는 경우

4) 임상시험 중 포함/제외 기준 등 중대한 계획서 위반 사항이 새롭게 발견되는 경우

5) 임상시험 조기 종료 기준에 부합하지 않지만 예정된 8회 치료 중 5회 이상 참여를 시행치 아니한 경우

6) 기타 이유로 대상자가 임상시험을 지속하기 어려울 것으로 보여 임상시험책임자/담당자가 시험을 중지하여야 한다고 판단한 경우

○ 임상시험 조기 종료

1) 임상시험 기간 중 증상이 호전되어, 환자 개인적 요청이 있고 임상시험 담당 의료진의 판단에 의해 더 이상의 시술이 필요 없다고 판단되는 경우에는 임상시험을 조기 종료 할 수 있다.

2) 임상 시험이 조기 종료된 대상자는 중지/탈락에 포함시키지 않으며, 치료에 모두 순응한 것으로 평가한다.

10. 치료순응도 (Treatment compliance)

임상시험자(또는 위임을 받은 자)는 임상시험과 관련한 통상치료 및 추나요법이 시행 될 때마다, 시험대상자의 치료 순응도가 지켜질 수 있도록 격려 및 지도를 시행한다. 계획된 치료 회수, 실제로 치료된 회수를 근거로 하여, 계획된 치료 횟수 대비 실제 치료받은 횟수의 비율을 통해서 치료 순응도를 계산한다. 치료 순응도는 무작위배정 이후 계획된 한의치료기술 예정된 치료 회수 8회 중 5회 이상이 되어야 한다. 치료 순응도를 평가하기 위해 임상시험자(또는 위임을 받은자)는 치료에 대한 기록을 남겨야 한다. 단, 증상호전으로 임상시험 조기 종료된 대상자는 환자가 치료에 모두 순응한 것으로 판단한다.

11. 구제약물(Rescue Drug)

스크리닝 방문 시점부터 참을 수 없는 통증이 발생하는 경우 임상시험담당자의 판단에 따라 마약성 진통제 및 마취제 등을 제외한, 제한적 소염진통제, 근이완제의 복용이 허용된다 (구제약물의 부작용 감소를 위한 소화기관 용약도 병용 투여 가능) 구제 약물을 복용할 경우, 복용일자 및 복용용량을 시험대상자 일지에 기록한다.

▷ 활용가능한 구제약물의 종류

골격근이완제, 항경련제, 소염진통제, 진통제, 신경통 치료제, 항경련제, 항우울제), 비마약성 진통제, 해열제, 약한 마약성 진통제(weak opioid), 소화용제.

12. 임상시험 조기 중단 기준

임상시험 진행 중 대상자의 안전과 임상시험의 진행에 심각한 영향을 줄 수 있는 중대한 이상반응(SAE)이 발생할 경우, 시험자의 판단에 따라 그 시점에서 진행중인 대상자의 임상시험 참여를 종료하고, 해당 이상반응이 임상시험 중재와 관련이 있다고 생각되는 경우, 모든 임상시험 절차의 진행을 중단한다.

중대한 이상반응(SAE) 이외의 이상 반응이라 할지라도, 부작용의 빈도나 양상 등에 있어서 더 이상 임상시험을 진행하는 것에 대한 윤리적 타당성이 의심되는 경우 연구진의 논의를 거쳐 임상시험을 중단한다. 이상 반응이 발생한 해당 대상자의 임상시험 지속 참여 여부는 대상자 자신과 연구진이 논의하여 결정하며, 연구진이 의학적으로 해당 대상자가 더 이상 임상시험의 참여를 지속할 수 없다고 판단하는 경우에는 강제적으로 참여를 종료시킬 수 있다.

13. 임상시험 종료 후 대상자의 진료 및 치료기준

임상 시험 참가 중 혹은 종료 후에 대상자가 비급성요통에 관련된 증상의 악화 혹은 부작용 등으로 관련 치료를 요구하는 경우 본원의 지침에 따라 적절한 진료를 제공한다. 치료를 요하는 대상자가 발생할 경우 모든 의료비용은 임상시험 보험계약에 의한 약관에 의거하여 제공한다. 임상시험이 종료 된 환자는 기대하지 않았던 지연 이상반응 발생에 대비하여 담당의사의 지시에 따라 언제든지 진료를 받을 수 있도록 한다. 다만, 임상시험 중에 이상반응이 발생한 경우 시험기관은 대상자가 회복할 때까지 적절한 의료조치를 취하며 추후 시험에 지속적으로 참가하는 것이 가능한지 여부에 관하여는 연구진들이 협의를 통해 결정하도록 한다.

14. 유효성평가 및 안전성 평가

1) 유효성 평가 (I군 & II군 동일 - visit 2, 10, 11)

- 1차 유효성 평가

  Baseline 및 이후 4주, 12주 시점에 임상시험에 참여하기 전과 비교하여 평가 당시 시점까지 시험대상의 요통에 대한 NRS 평가를 시행한다.

1) Numeral Rating Scale (NRS) - 요통

NRS는 주로 전반적인 통증의 강도를 평가하는데 사용되며, 환자가 자신의 통증 정도에 해당하는 숫자를 주어진 숫자중에서 선택하는 방식으로 평가를 시행하게 된다 (0부터 10까지의 숫자를 사용하는 NRS-11 사용 예정).

- 현재 환자분의 허리부위 통증의 강도는 통증이 없는 상태를 0, 상상할 수 있는 가장 심한 통증을 10이라 가정하여 숫자로 표현할 때 어느정도에 해당합니까?

통증 없음	0	1	2	3	4	5	6	7	8	9	10	상상할수 있는 가장 심한 통증

■ 2차 유효성 평가

1) NRS - 하지부 방사통

Baseline 및 이후 4주, 12주 시점에 임상시험에 참여하기 전과 비교하여 평가 당시 시점까지 시험대상의 하지부 방사통에 대한 NRS 평가를 시행한다.

- 현재 환자분의 하지부위 통증의 강도는 통증이 없는 상태를 0, 상상할 수 있는 가장 심한 통증을 10이라 가정하여 숫자로 표현할 때 어느정도에 해당합니까?

통증 없음	0	1	2	3	4	5	6	7	8	9	10	상상할수 있는 가장 심한 통증

2) Oswestry disability index (ODI) - 오스웨스트리기능장애 평가

(ODI)를 통한 기능 평가를 시행하여 이에 따른 증상 개선도를 평가한다(단, 한국의 문화적 특성을 반영하여 제 8항 성생활에 관한 질문은 시행치 않기로 함).

<Oswestry Disability Index(오스웨스트리 요통장애 지수)>

본 설문지는 당신이 허리(혹은 다리)의 문제로 인해 일상 생활에서 얼마나 제한이 있는지를 알기 위해 제작되었습니다. 모든 문항에 답하여 주시되, 각 문항마다 오늘의 상태에 가장 적당한 한 칸에만 표기하십시오.

제 1 항 - 통증 정도
□0 나는 현재 통증이 전혀 없다.
□1 현재 매우 가벼운 통증이 있다.
□2 현재 통증이 조금 있다.
□3 현재 통증이 조금 심하다.
□4 현재 통증이 아주 심하다.
□5 현재 통증이 상상할 수 없이 심하다.

제 2 항 - 개인 위생 (씻기 , 옷 입기 등)
□0 나는 별다른 통증이 없이 나 자신을 챙길 수 있다.
□1 보통 나 자신을 챙길 수 있으나, 통증이 있다.
□2 나 자신을 챙기는데 고통스러워서, 천천히 조심스럽게 해야 한다.
□3 통증 때문에 어느 정도 도움이 필요하거나, 혼자서 할 수는 있다.
□4 매일 도움이 없이는 나 자신을 챙기기가 어렵다.
□5 옷을 입거나 씻는 게 어렵고, 보통은 누워있다.

제 3 항 - 물건 들기
□0 나는 무거운 물건을 통증 없이 들 수 있다.
□1 무거운 물건을 들 수 있으나, 약간 통증이 있다.
□2 통증 때문에 바닥에 있는 무거운 물건을 들지 못하나, 들기 쉬운 곳에 있으면 들 수 있다.
□3 통증 때문에 무거운 물건을 들 수 없지만, 들기 쉬운 곳에 있는 무겁지 않은 물건은 들 수 있다.
□4 아주 가벼운 물건만 들 수 있다.
□5 아무것도 들거나 나를 수 없다.

제 4 항 - 걷기
□0 나는 걷는데 아무런 지장이 없다.
□1 통증 때문에 1 Km 이상 걷지 못한다.

□2 통증 때문에 500 m 이상 걷지 못한다.
□3 통증 때문에 100 m 이상 걷지 못한다.
□4 지팡이나 목발이 있어야만 걷는다.
□5 대부분 자리에 누워있으며, 화장실도 기어가야 한다.

제 5 항 - 앉기
□0 나는 어떤 의자에서든지 오래 앉아 있을 수 있다.
□1 편한 의자라면 오래 앉아 있을 수 있다.
□2 통증 때문에 1 시간 이상 앉아 있을 수 없다.
□3 통증 때문에 30 분 이상 앉아 있을 수 없다.
□4 통증 때문에 10 분 이상 앉아 있을 수 없다.
□5 통증 때문에 전혀 앉아 있을 수 없다.

제 6 항 - 서있기
□0 나는 통증 없이 얼마든지 서 있을 수 있다.
□1 오래 서 있을 수 있으나 약간 통증이 있다.
□2 통증 때문에 1 시간 이상 서 있을 수 없다.
□3 통증 때문에 30 분 이상 서 있을 수 없다.
□4 통증 때문에 10 분 이상 서 있을 수 없다.
□5 통증 때문에 전혀 서 있을 수 없다.

제 7 항 - 잠자기
□0 나는 통증 없이 잘 잔다.
□1 통증 때문에 가끔 잠자는 데 방해를 받는다.
□2 통증 때문에 6 시간 이상 잠을 자지 못한다.
□3 통증 때문에 4 시간 이상 잠을 자지 못한다.
□4 통증 때문에 2 시간 이상 잠을 자지 못한다.
□5 통증 때문에 전혀 잠을 자지 못한다.

제 8 항 - 성생활 (해당 시)
□0 나는 정상적으로 성생활을 하고 통증이 없다.
□1 정상적으로 성생활을 하나 가끔 통증을 느낀다.
□2 거의 정상적으로 성생활을 하나 통증을 심하게 느낀다.
□3 통증 때문에 성생활이 매우 제한적이다.
□4 통증 때문에 성생활을 거의 할 수 없다.
□5 통증 때문에 성 관계를 전혀 갖지 않는다.

제 9 항 - 사회생활
□0 나는 밖에서 사람들과 어울리는 데 지장이 없다.
□1 밖에서 사람들과 어울리는데 지장은 없으나, 그로 인해 통증이 심해진다.
□2 밖에서 사람들과 어울리는데 지장은 없으나, 통증 때문에 운동하는 데에는 지장이 있다. (예: 스포츠 등)
□3 통증 때문에 밖에서 사람들과 어울리는데 지장이 있으며, 자주 외출하지 못한다.
□4 통증 때문에 집에서만 사람들과 어울린다.
□5 통증 때문에 사람들과 전혀 어울리지 못한다.

제 10 항 - 여행
□0 나는 통증 없이 어디든 여행할 수 있다.
□1 어디든 여행할 수 있으나, 약간 통증이 있다.
□2 통증은 있으나, 2 시간 이상 차를 탈 수는 있다.
□3 통증 때문에 1 시간 이상 차를 탈 수 없다.
□4 통증 때문에 30 분 이상 차를 탈 수 없다.
□5 통증 때문에 치료를 받으러 가는 일 외에는 차를 탈 수 없다.

<한국어판 오스웨스트리 장애지수 평가>

3) European Quality of Life 5 Dimension (EQ-5D)

임상시험 참여전 screening test(0주), 4주, 12주째에 EQ-5D의 설문 문항을 활용하여 임상시험에 참여하기 전과 비교하여 해당시점까지의 삶의질 변화를 평가한다. EQ-5D는 운동능력(mobility), 자기관리(self-care), 일상활동(usual activity), 통증/불편감(pain/disability), 그리고 불안/우울(anxiety/depression)과 같은 5개 항목에 대한 현 상태를 평가하는데 널리 활용되는 삶의 질 평가 도구이다.

본인의 건강 상태 평가 (EQ-5D-5L)

문 1) 아래의 각 문항에서, 오늘 귀하의 건강 상태를 가장 잘 나타낸 박스 한 개를 체크해 주십시오.

16 **이동성**

- 나는 걷는데 전혀 지장이 없다 ☐ 1
- 나는 걷는데 약간 지장이 있다 ☐ 2
- 나는 걷는데 중간 정도의 지장이 있다 ☐ 3
- 나는 걷는데 심한 지장이 있다 ☐ 4
- 나는 걸을 수 없다 ☐ 5

17 **자기 관리**

- 나는 혼자 씻거나 옷을 입는데 전혀 지장이 없다 ☐ 1
- 나는 혼자 씻거나 옷을 입는데 약간 지장이 있다 ☐ 2
- 나는 혼자 씻거나 옷을 입는데 중간 정도의 지장이 있다 ☐ 3
- 나는 혼자 씻거나 옷을 입는데 심한 지장이 있다 ☐ 4
- 나는 혼자 씻거나 옷을 입을 수 없다 ☐ 5

18 **일상 활동 (예 : 일, 공부, 가사일, 가족 또는 여가 활동)**

- 나는 일상 활동을 하는데 전혀 지장이 없다 ☐ 1
- 나는 일상 활동을 하는데 약간 지장이 있다 ☐ 2
- 나는 일상 활동을 하는데 중간 정도의 지장이 있다 ☐ 3
- 나는 일상 활동을 하는데 심한 지장이 있다 ☐ 4
- 나는 일상 활동을 할 수 없다 ☐ 5

19 **통증/불편감**

- 나는 전혀 통증이나 불편감이 없다 ☐ 1
- 나는 약간 통증이나 불편감이 있다 ☐ 2
- 나는 중간 정도의 통증이나 불편감이 있다 ☐ 3
- 나는 심한 통증이나 불편감이 있다 ☐ 4
- 나는 극심한 통증이나 불편감이 있다 ☐ 5

20 **불안/우울**

- 나는 전혀 불안하거나 우울하지 않다 ☐ 1
- 나는 약간 불안하거나 우울하다 ☐ 2
- 나는 중간 정도로 불안하거나 우울하다 ☐ 3
- 나는 심하게 불안하거나 우울하다 ☐ 4
- 나는 극도로 불안하거나 우울하다 ☐ 5

<EQ-5D-5L 한국어판>

4) RMDQ 한국어판[2]

RMDQ는 Roland M, Morris R이 1983년에 고안한 설문지로 요추 질환과 관련된 많은 임상연구에서 주된 평가지표로 활용하고 있다. 요통으로 인해서 발생할 수 있는 일상적인 장애에 대한 상황을 24개의 설문 항목으로 구성하여, 각 항목에 대해서 본인이 해당하지 않는지 '예/아니오'로 응답하는 방식으로 구성되어 있다. 본 연구에서는 baseline test(0주), 4주, 12주째에 설문 문항을 활용하여 임상시험에 참여하기 전과 비교하여 해당시점까지의 통증의 변화정도

[2] Lee JS, Lee DH, Suh KT, Kim JI, Lim JM, Goh TS. Validation of the Korean version of the Roland-Morris Disability Questionnaire. Eur Spine J. 2011;20(12):2115-9

를 측정하게 된다.

1. 나는 허리 통증 때문에 대부분의 시간을 집에서 보낸다.
2. 나는 허리를 편하게 하기 위하여 자세를 자주 바꾼다.
3. 나는 허리 통증 때문에 평소보다 천천히 걷는다.
4. 나는 허리 통증 때문에 평소에 하던 집안 일들을 하지 않는다.
5. 나는 허리 통증 때문에 계단을 오를 때 난간을 잡고 오른다.
6. 나는 허리 통증 때문에 예전 보다 자주 누워서 쉰다.
7. 나는 허리 통증 때문에 소파에서 일어날 때 무언가를 잡고 일어나야 한다.
8. 나는 허리 통증 때문에 다른 사람들에게 무언가를 해달라고 한다.
9. 나는 허리 통증 때문에 평소보다 옷을 늦게 입는다.
10. 나는 허리 통증 때문에 잠시 동안만 서 있을 수 있다.
11. 나는 허리 통증 때문에 허리를 구부리거나 무릎 꿇고 앉기가 힘들다.
12. 나는 허리 통증 때문에 의자에서 일어나기가 힘들다.
13. 나는 거의 항상 허리 통증이 있다.
14. 나는 허리 통증 때문에 잠자리에서 돌아눕기 힘들다.
15. 나는 허리 통증 때문에 입맛이 없다.
16. 나는 허리 통증 때문에 양말 (혹은 스타킹)을 신기가 힘들다.
17. 나는 허리 통증 때문에 가까운 거리만 걷는다.
18. 나는 허리 통증 때문에 잠을 잘 못 잔다.
19. 나는 허리 통증 때문에 옷을 입을 때 다른 사람의 도움을 받는다.
20. 나는 허리 통증 때문에 대부분의 시간을 앉아서 지낸다.
21. 나는 허리 통증 때문에 집안의 힘든 일들을 하지 않는다.
22. 나는 허리 통증 때문에 평소보다 더 짜증이 난다.
23. 나는 허리 통증 때문에 평소보다 천천히 계단을 오른다.
24. 나는 허리 통증 때문에 대부분의 시간을 누워서 지낸다.

< Roland Morris Disability Questionnaire 한국어판>

5) 이학적평가 – thoraco lumbar spine range of motion (ROM)

- 본 연구에서는 baseline test(0주), 4주, 12주째시점의 흉요추 ROM 평가를 시행하며, 굴곡(flexion), 신전(extension), 측굴(lateral flexsion –우,좌), 회전(rotation- 우,좌)로 나누어 시행한다.

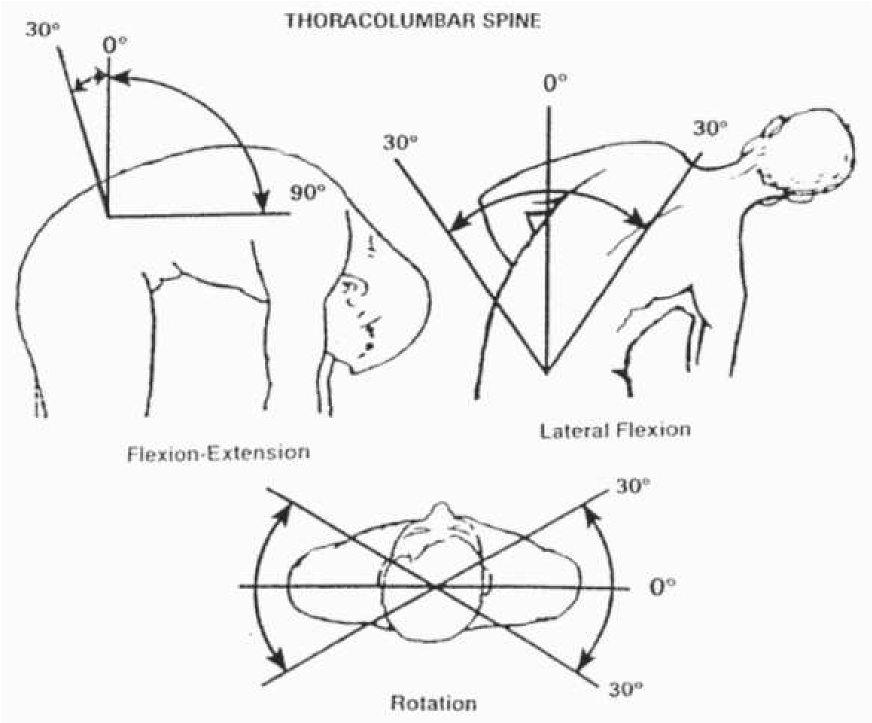

5) 영상평가

- screening test(-1주), 4주, 12주째시점에서 표준화 절차에 따른 L-spine AP & Lateral standing view 영상을 촬영
- 촬영된 영상을 바탕으로, 한국한의학연구원에서 제공하는 dicom labeling 프로그램을 통해 추나의학적 변위 유형 진단을 위한 추체별 상대적 각도(flexion, extension, lateral bending,

rotation) 및 추나의학적 변위 진단 결과 값을 비교한다.

요추 레벨	lateral view	Ap view		변위 진단 유형
	굴곡/신전각도	측굴각도 (우:+, 좌:-)	회전각도 (우:+, 좌:-)	
L1				
L2				
L3				
L4				
L5				

<영상 평가를 통한 요추 레벨별 각도 및 변위 진단 값 표기 양식>

2) 탐색적 유효성 평가

(1) 추가적인 시술 및 수술 시행 여부

- 모든 임상시험 대상자를 대상으로 처치 종료 8 주 후 최종 방문일에 허리통증과 관련한 의과의 침습적 시술[주사치료(신경차단술) 고주파 열응고술 등] 시행 여부, 강한마약성 진통제 사용여부(strong opioid), 허리 수술 여부를 조사하게 된다. 추가적 시술 및 수술 시행 여부에 대한 평가는 증상이 호전되어 임상시험이 조기 종료된 환자에게는 시행하지 않으며, 중도탈락 환자의 경우 탈락 8 주 후 해당 평가를 시행한다. 단, 중도 탈락자에 대한 탐색적 유효성 평가는 직접내원 대신 전화 상담으로 대체할 수 있다.

(2) 치료 조기 종료 여부

- 임상시험 기간 중 증상이 호전되어, 환자 개인적 요청이 있고 임상시험 담당 의료진의 판단에 의해 더 이상의 시술이 필요 없다고 판단되어 임상시험이 조기 종료된 환자를 각 치료 군별 나누어 확인한다.

(3) 구제 약물 사용량

- 각 군간 임상시험 기간 및 최종 평가 기간까지 사용된 구제 약물의 사용 횟수, 용량, 종류, 투여시기에 대한 정보를 수집 후 비교 평가한다.

3) 치료 반응군(responder) 및 비반응군(non-responder) 평가

(1) NRS 를 기준으로 요통의 minimal clinically important difference (MCID)인 2를 기준으로 하여, screening 에 비해 2이상 NRS 점수가 줄어든 경우 치료에 반응한 집단으로, 그렇지 않은 경우 비반응 집단을 구분한다.
(2) 증상이 호전되어 치료 조기 종료가 이루어진 경우 치료 반응군으로 포함한다.

4) 안전성 평가

(1) 신체검사, 활력징후(Vital signs) : 무작위 배정 이후 모든 방문에서 시행

- 신체검사, 활력징후 결과를 총괄적으로 검토하여 각 검사의 정상/비정상은 개별 대상자에서 판정한다.

(2) 자·타각 증상 등 이상반응 등 이상반응 확인 : 무작위 배정 이후 모든 방문에서 시행

- 시험담당자는 임상시험 중 발생하는 모든 이상반응 및 병용의약품을 기록해야 한다.
- 증례기록서에는 이상반응 및 병용의약품에 대해 기록한다. 이상반응의 경우 이상반응의 증상 및 징후, 지속시간(시작일/종료일), 중증도, 결과, 중대성, 중재와의 인과관계, 이상반응과 관련하여 취해진 조치 등에 관하여 기록한다.
- 임상시험 참여 이전부터 존재한 증상, 징후가 있는 경우, 이상반응으로 기록하지 않는다.

단, 임상시험 참여 이후 해당 증상, 징후의 빈도, 중증도, 범위 등에 변화가 있는 경우에는 이상반응으로 기록한다.

- 또한, 병용의약품의 경우에는 성분명, 투여량, 투여기간, 투여사유 등에 관하여 자세히 기록한다(구체적인 평가방법은 18. 이상반응의 보고방법 및 평가기준 참고)

15. 통계분석

본 임상시험으로부터 획득한 자료의 평가를 위한 분석군(analysis set)은 크게 다음과 같이 구분한다.

- Intention-to-treat (ITT) set: 무작위 배정을 받고 최소 한번 이상 임상시험용 치료를 받은 모든 임상시험 대상자로 구성된 분석군
- Full analysis (FA) set: ITT 원칙을 준수하면서, 무작위 배정을 받고 최소 한번 이상 임상시험용 치료를 받은 모든 대상자에 대해 적어도 한 번 이상 유효성 평가변수를 획득한 분석군
- PP(Per-Protocol)군은 다음 조건을 만족하는 대상자들을 포함한다.
  1) 선정기준, 제외기준을 모두 충족시키는 대상자
  2) 미리 정한 최소 횟수 이상의 치료를 완료한 경우(총 8 회의 치료 중 5 회 이상의 침도 시술을 받은 환자)
  3) 증상 호전으로 인해 치료 종결 기준에 부합하여 치료가 종결된 경우
- Safety analysis (SA) set: 무작위 배정 이후 최소 한 번 이상 임상시험용 치료를 받고 안전성 평가가 이루어진 모든 대상자로 구성된 분석군

본 임상시험에서 분석의 일반적인 원칙은 통계적 검정의 유의수준은 양측 α=0.05 으로 한다. 유효성 평가변수를 포함한 모든 유효성 분석은 full analysis set(FAS) 분석군을 주분석으로 시행하고, 보조분석으로 PP(Per-Protocol)군 분석도 시행한다. 안전성 분석은 무작위 배정을 받아 한번이라도 시술을 받은 ITT 군에 대하여 시행한다. 결측치 대체는 FAS 군에서만 적용된다. 유효성 평가변수 중, 연속형 평가변수의 결측치에 대해서는 결측치가 발생한 시점 이전 시점에서 얻어진 자료로 대체(Last observation carried forward, LOCF)하여 분석한다. 나머지 변수에 대해서는 결측치 대체하지 않고 원자료 그대로 분석한다.

1) 인구학적 정보 및 치료 전 특성에 대한 분석 방법

인구학적 정보 및 특성에 대해 기술 통계량을 기술한다. 연속형 자료는 정규성 만족 여부에 따라 관측치 수, 평균, 표준편차 또는 중앙값, 25%분위수, 75%분위수로 나타내고, 범주형 자료의 경우 빈도수와 백분율로 나타낸다. 연속형 자료에 대한 군간 평균차이 검정을 위하여 정규성 가정을 만족하면 Independent t-test 를 시행하고, 만약 정규성 가정을 만족하지 않으면 Wilcoxon's rank sum test 를 시행한다. 그리고 범주형 자료에 대하여 군간 차이 검정을 위하여 Chi-square test 를 시행하거나 셀 기대도수가 5 미만인 경우가 25%를 넘을 경우 Fisher's exact test 를 시행한다.

2) 유효성 평가

유효성 분석은 모든 분석 대상자군(FAS)를 주 분석군으로 하고, 순응 임상시험대상자군(PP sets)을 보조 분석군으로 병행하여 분석한다. 모든 통계 검정은 유의수준 5%에서 양측 검정으로 수행하는 것을 원칙으로 하며, 양측 95% 신뢰구간을 제시한다. 각 치료군별 및 방문별로 기술통계량을 제시하며, 기저치 대비 각 방문과의 차이에 대해서는 기술통계량 및 95% 신뢰구간을 제시한다.

가. 일차 유효성 평가 변수 분석

▶ 각 측정 시점의 요통과 관련하여 NRS 점수의 기저시점(0 주) 대비 치료 종료(4 주)와 추적관찰(12 주)에서의 변화량에 대하여 치료군별 기술 통계량(시험대상자 수, 평균, 표준편차)을 제시한다.

▶ 기저시점(0 주) 대비 치료종료(4 주) 시점에서의 요통의 NRS 변화량에 대한 각 군내 검정은 정규성 만족 여부에 따라 paired t-test 또는 wilcoxon signed rank test 를 실시한다. 대조군 대비 치료군의 효과는 기저시점 대비 치료종료 시점의 NRS 변화량에 대한 치료군을 effect 로 하고, 기저시점 NRS 점수를 공변량으로 포함하는 ANCOVA model 을 이용하여 분석한다. 대조군 대비 치료군간 least-square mean(LSM) 차이를 구하고, 그 차이에 대한 양측 95% 신뢰구간 및 p-value 를 제시한다.

나. 이차유효성 평가변수분석

▶ 이차유효성 평가는 다음과 같은 항목을 시행하며, 일차 유효성 평가변수의 분석 방법을 준용해서 기저시점(0 주) 대비 치료 종료(4 주)와 추적관찰(12 주) 사이의 변화량을 측정한다.

① 기저시점(0 주) 대비 치료 종료(4 주)와 추적관찰(12 주)에서의 하지통증의 NRS 변화량

② 요통과 관련한 주관적 통증 강도 및 삶의 질 의 평가

- 기저시점(0 주) 대비 치료 종료(4 주)와 추적관찰(12 주)에서의 ODI, EQ-5D, RMDQ 의 변화량

다. 탐색적 유효성 평가

▶ 연구 종료 후 신경차단술의 추가 시술 또는 수술 여부와 관련한 평가

시술 종료후 8 주이내의 기간에 환자의 통증 제어를 위한 추가적인 시술 (예: 신경차단술)이 수행되었거나, 수술이 시행된 경우에 대해 조사를 시행하여, 이를 바탕으로 추가 시술율을 평가하고자 함. 군간 차이가 확인될 경우 사후 검증을 시행한다. 군간 추가 시술과 수술의 시행율 비교는 Chi-square test 또는 Fisher's exact test 등을 실시한다.

**추가 시술 시행율 = (추가적인 시술 또는 수술을 시행한 환자/전체 연구를 종료한 환자)*100**

▶ 치료 조기 종결율 평가

예정된 시술이 종료되기 전 치료 종결 기준에 부합하여, 치료가 조기 종결된 경우 각 군별 연구 조기 종료 비율을 평가하고자 한다. 치료조기 종료율의 군간 비교는 Chi-square test 또는 Fisher's exact test 등을 실시한다.

**연구 조기 종료 비율 = (연구가 조기 종료된 환자 / 전체 연구를 종료한 환자)*100**

▶ 구제 약물 사용 분석

각 피험자별 사용된 구제약물의 유형, 투여량, 투여시기에 대한 정보를 수집하여 각 군 별 구제약의 종류에 따른 사용 횟수 및 사용량에 대한 군간 비교는 independent t test 혹은 Wilcoxon signed rank test를 시행한다.

라. Respondor 및 Non-responder 구분 및 Post-hoc analysis

▶ 치료 반응자의 정의

① Baseline에서 평가시일 까지의 NRS의 감소량을 기반으로 구체적인 minimal clinically important difference (CID)인 NRS 2를 기준으로 한다. 따라서 다음과 같이 정의한다.

예시) NRS 2.5 이상의 통증감소가 이루어진 환자를 CID 로 규정하고 이를 바탕으로<br>치료반응집단 / 비반응 집단으로 구분

② 앞서 언급한 기준을 바탕으로, 증상 호전으로 인한 연구가 조기 종결된 경우 치료 반응 집단으로 합계한다.

▶ Post-hoc analysis

결정된 Respondor 및 Non-responder 의 기준을 기준으로 각 치료군 별 치료 결과차이에 대해 통계전문가를 통한 적절한 Post-hoc analysis 를 시행한다.

3) 안전성 평가

이상반응 발생에 대해서 발생건수, 발생한 대상자수, 중증도(severity), 중재와의 인과관계를 용량 군에 따라 기술통계학적으로 분석하고 필요에 따라 비모수적 방법을 적용할 수 있다.

활력징후, 신체검사, 환자의 주관적 호소 등의 결과를 총괄적으로 검토하여 연구자에 의해 임상적으로 유의 하다고 판단된 검사 항목에 대하여 필요에 따라 통계분석을 실시한다. 자료의 성격에 따라 연속형 자료는 방문별로 관측치 수, 평균, 표준편차, 중앙값, 최소값, 최대값을 제시하고, 그 변화를 군내차이 검정 paired t-test 혹은 Wilcoxon signed rank test 를 이용하여 분석한다. 이상반응에 대한 분석은 Chi-square test 혹은 Fisher's Exact test 를 실시한다.또한, 임상적으로 유의할 만한 변화가 발생하여 군간 비교가 필요할 때 GEE(generalized Estimating Equation)분석 등을 이용하여 통계적으로 유의한 차이가 있는지 비교한다.

16. **이상반응의 보고방법 및 평가기준**

추나요법시술 후 예측되는 부작용으로는 시술부위 통증의 악화, 피로감 등이며, 드물게 신경손상 및 골절등이 발생할 수 있다. ICT 의 경우 시술부위 피부 자극이나, 화상, 수포, 시술 후 불편감 등이 있을 수 있다. 본 연구에서 시술과 관련한 이상 반응의 범주는 이와 같은 내용을 기본으로 하며, 추나요법 시술과 관련한 이상 반응의 범주는 통증의 악화, 피로감, 골절, 신경손상으로 하며, 통상치료군의 경우 화상, 수포, 통증의 악화까지를 이상반응의 범주로 설정한다.

본 연구의 중재시술과 관계없는 이상반응은 중대한 이상반응으로 분류하지 않는다. 이상반응 보고반응 및 평가 기준은 이후 기술하는 바에 따르며, 일반적인 약물중재 연구에서 활용하는 바탕으로 이상반응의 정의, 보고방법 및 평가기준을 적용한다. 시험자는 임상시험 중 발생한 모든 이상반응에 대하여 기록하여야 한다. 증례기록서에는 이상반응의 증상 및 징후, 지속시간(시작일/종료일), 중증도, 경과, 결과, 중대성, 임상시험 시술과의 인과관계, 이상반응과 관련하여 취해진 조치 등에 관하여 기록한다.

1) 정의

(1) 이상반응(Adverse Event, AE)

이상반응이란 임상시험시술을 받은 시험대상자에서 발생한 바람직하지 않고 의도되지 않은 증후, 증상(symptom) 또는 질병을 말하며, 해당 임상시험시술과 반드시 인과관계를 가져야 하는 것은 아니다.

(2) 이상시술반응(Adverse treatment Reaction, ATR)

본래 이상 약물 반응 (adverse drug reaction)이란 임상시험용의약품의 임의의 용량에서 발생한, 모든 유해하고 의도되지 않은 반응으로서, 임상시험용 의약품과의 인과관계를 배제할 수 없는

경우를 말한다. 본 연구는 약물투여 연구가 아닌, 시술과 관련한 연구로 이러한 기준을 바탕으로 적어도 임상시험 시술과 관련한 이상반응과의 관련성이 있을 수 있다는 합리적인 가능성, 즉 관련성을 완전히 배제할 수 없는 경우를 이상시술 반응으로 규정하기로 하였다.

(3) 예상하지 못한 이상시술반응(Unexpected Adverse treatment Reaction)
이용 가능한 시술 관련 정보 (예를 들어 임상시험자자료집 또는 신경차단술에 활용되는 의약품의 첨부문서)에 비추어 이상시술반응의 양상이나 위해의 정도에서 차이가 나는 것을 말한다.

(4) 중대한 이상반응(Serious AE)
임상시험에 사용되는 시술에서 발생한 이상반응 중에서 다음 중 하나에 해당하는 경우를 말한다.

- 시험대상자가 시험기간 중 사망
- 생명을 위협하는 경우 (시험대상자가 그 사건의 발생시점에서 죽음의 위협에 놓여 있는 경우이며, 만약 더 심각해지면 죽을 수도 있다는 가정적인 것이 아님)
- 입원을 요하거나 이미 입원한 시험대상자의 입원 기일을 늘려야 하는 경우 (단, 단순요양등을 목적으로 한 입원은 중대한 이상반응에 해당하지 않는다)
- 지속적 또는 의미 있는 불구나 기능 저하를 초래 경우

위에서 열거한 상황이 아니더라도 의학적으로 시험대상자의 안위와 건강상태에 중대한 영향을 미칠 것으로 사료되는 상황이 발생한 경우, 담당의사 및 관련 전문가의 의학적 판단에 따라 중대한 이상반응으로 간주할 것인가의 여부를 결정하고 이에 따라 적절한 조치를 취한다

(5) 중대하고 예상하지 못한 이상 시술반응(Serious and Unexpected Adverse Treatment Reaction)
예상하지 못한 이상시술반응이란 이용 가능한 시술 관련 정보에 모순되는 성질(nature) 또는 중대한 정도(severity)를 말하며, 중대하고 예상하지 못한 이상시술반응이 발현되었을 경우에는 의뢰자에게 신속보고가 필요하다.

2) 이상반응의 평가

- 본 임상시험에서 이상반응 평가는 임상시험 시작 전에 관찰되지 않은 증상이 투여기간 중에 새로이 나타난 증상으로서 임상시험용의약품과의 인과관계에 상관없이 의도하지 않았던 증후 및 증상과 관련된 일시적인 현상 등을 총칭한다.
- 임상시험 시술과 관련한 이상반응으로 예상되는 현상(증세 및 증상, 시작일, 지속기간 등)은 이상반응란에 빠짐없이 기록되어야 한다.
- 이상반응 정도에 대한 평가는 임상시험책임자 및 담당자가 '**4) 임상시술과의 인과관계 평가**'를 참고하여 증상의 경중에 따라 단계별로 평가하는 것을 원칙으로 한다.
- 임상시험용의약품과의 인과관계는 임상시험책임자 및 담당자가 '**4) 임상시험시술과의 인과관계 평가**'에 따라 6 단계로 분류하여 평가를 실시한다.

3) 이상반응의 기록

이상반응의 중등도는 임상시험책임자 및 담당자의 진료를 통하여 다음의 기준에 따라 기록한다.

Grade 1	Mild AE – 징후 또는 증상의 자각, 쉽게 내약성을 나타냄
Grade 2	Moderate AE – 정상적인 일상생활에 방해를 일으킬 수 있는 불편감
Grade 3	Severe AE – 정상적인 일상 생활 수행 불가능

시험기간 중 나타나는 모든 이상반응은 시험책임자 및 임상시험담당자가 기록한다.
< 증례기록지(CRF)에 기록되어야 할 사항 >
1. 이상반응 증상 및 징후
2. 이상반응 시작일
3. 이상반응 종료일
4. 이상반응 중증도
5. 이상반응 결과
6. 이상반응과 임상시험용의약품과의 인과관계
7. 이상반응에 대해 취해진 조치

4) 임상시술과의 인과관계 평가

이상반응 발현 시 임상시험용시술과의 연관성 여부는 임상시험담당자가 다음과 같이 분류하고, 필요 시 임상시험담당자의 견해를 기재한다. 이상반응 발생 시 이상반응과 임상시험용의약품 투약과의 관련 정도는 다음의 기준에 따라 판정한다.

① 확실함 (Certain)
- 임상시험용 시술 등의 적용, 사용과의 전후관계가 타당하고 다른 의약품이나 화학물질 또는 수반하는 질환으로 설명되지 아니하며, 임상시험용 시술 등의 적용 중단 시 임상적으로 타당한 반응을 보이고, 필요에 따른 재적용 시 현상학적으로 결정적인 경우

② 상당히 확실함 (Probable/likely)
- 임상시험용 시술의 적용, 사용과의 시간적 관계가 합당하고 다른 의약품이나 화학물질 또는 수반하는 질환에 따른 것으로 보이지 아니하며, 임상시험용 시술 적용 중단 시 임상적으로 합당한 반응을 보이는 경우(재적용 정보 없음)

③ 가능함 (Possible)
- 임상시험용 시술 등의 적용, 사용과의 시간적 관계가 합당하나 다른 의약품이나 화학물질 또는 수반하는 질환에 따른 것으로도 설명되며, 적용 중단에 관한 정보가 부족하거나 불명확한 경우

④ 가능성 적음 (Unlikely)
- 임상시험용 시술의 적용, 사용과 인과관계가 있을 것 같지 않은 일시적 사례이고, 다른 의약품이나 화학물질 또는 잠재적 질환에 따른 것으로도 타당한 설명이 가능한 경우

⑤ 평가 곤란 (Conditional/unclassified)
- 적정한 평가를 위해 더 많은 자료가 필요하거나 추가 자료를 검토 중인 경우

⑥ 평가 불가 (Unassesable/unclassifiable)
- 정보가 불충분하거나 상충되어 판단할 수 없고 이를 보완하거나 확인할 수 없는 경우

5) 이상반응과 관련하여 취해진 조치

1= 시술 중지	Treatment withdrawn
2= 시술 감소	Treatment reduced
3= 시술 증량	Treatment increased
4= 시술에 변화 없음 (기존 시술 유지)	Treatment not change
5= 치료약물 병용 투여	Concomitant medication taken
6= 비약물 치료	Non-drug therapy given
7= 알 수 없음	Unknown
8= 해당사항 없음	Not Applicable

6) 이상반응의 결과

1= 회복됨/해결됨	Recovered/Resolved
2= 회복중임/해결중임	Recovering/Resolving
3= 회복되지 않음/해결되지 않음	Not recovered/Not resolved

4= 회복되었으나 후유증이 남음/해결되었으나 후유증이 남음	Recovered/Resolved with sequelae
5= 사망	Fatal
6= 알 수 없음	Unknown

7) 이상반응의 보고

임상시험기간 중 중대한 이상반응/이상약물반응이 발생할 경우, 임상시험책임자 및 담당자는 대상자의 안전에 만전을 기해야 하며, 신속하고 적절한 조치를 취하여 이상반응을 최소화하여야 한다. 임상시험담당자는 즉시 임상시험책임자에게 보고하여야 하며 각 실시기관 IRB SOP 에 정해진 기한에 따라 IRB 에 보고하여야 한다.

임상시험책임자 및 담당자는 시험대상자 또는 보호자에게 임상시험시술 후 나타날 수 있는 모든 이상반응에 대하여 교육을 실시하고 투여 후 나타나는 모든 현상에 대하여 보고하도록 교육을 실시한다. 임상시험시술 후 전신적 또는 임상병리학적으로 나타나는 제반 증상에 대하여 종류, 발생시간, 정도, 처치, 치료약제, 경과, 임상시험시술과의 인과관계 등에 대한 기록 및 보관은 임상시험 관리기준에 준하여 증례기록서에 기입한다.

임상시험책임자는 임상시험결과보고 시 임상시험 기간 중 발생된 모든 증상에 대하여 서술하고 평가를 실시하며, 시험기간 중 "중대한 이상반응(serious adverse event)" 발생시에는 임상시험심사위원회 (의학연구 윤리심의위원회, IRB)에 보고하며, 또한 연구주관기관인 가톨릭관동대학교 국제성모병원 한의과에도 보고하여 시험의 지속 또는 중단 여부를 결정한다.

중대하고 예상하지 못한 이상시술반응의 경우에는 임상시험심사위원회(의학연구윤리심의위원회, IRB)에도 보고하도록 한다. 추가적인 안전성 정보를 주기적으로 해당 이상시술반응이 종결(해당 이상시술 반응의 소실 또는 추적조사의 불가 등)될 때까지 보고하여야 하며, 임상시험이 종료된 후에도 임상시술 후 7 일 내에 발생한 이상반응의 경우에는 위의 이상반응 보고원칙에 따라 보고하는 것으로 한다. 임상시험책임자는 임상시험을 시행함에 있어서 모든 제반 사항을 Declaration of Helsinki 에 준하여 시행한다.

8) 이상반응의 추적관찰

임상시험 책임자 또는 담당자는 이상반응이 나타난 대상자에 대해 증상이 완화되고 비정상적 진단검사실 검사치가 기준치로 회복되거나, 혹은 관찰된 변화에 대해 만족스러운 설명이 될 때까지 추적 관찰한다. 시험담당자는 IRB의 요구가 있는 경우에는 관찰보고서를 IRB에 제출한다.

이상반응 발생시 조치사항

본 시험기간 중 임상시험책임자 및 담당자는 시험대상자의 안전에 만전을 기하여야 하며, 중대한 이상시술반응 발생시에는 해당 시험대상자의 시험을 중지하고 신속하고 적절한 조치를 취하여 이상반응을 최소화하여야 한다. 임상시험 중 "중대한 이상시술반응" 발생시 각 담당자의 의무는 다음과 같다.

(1) 임상시험책임자의 의무

임상시험책임자는 임상시험 중 중대한 이상반응이 발생한 때에는 임상시험의뢰자에게 24 시간 이내에 즉시 보고하며, 적시에 임상시험심사위원회 (의학연구윤리심의위원회, IRB)에 보고하여야 한다.

(2) 임상시험담당자의 의무

임상시험담당자는 임상시험 실시 중에 중대한 이상반응 등이 발생한 경우에는 임상시험책임자 및 임상시험심사위원회 (의학연구윤리심의위원회, IRB)에게 IRB SOP 규정에 따른 기한 내에 보고하여야 한다.

(3) 임상시험심사위원회(의학연구윤리심의위원회, IRB)의 의무
임상시험심사위원회(의학연구윤리심의위원회, IRB)는 중대한 이상반응이 나타난 경우에는 임상시험의 일부 또는 전부에 대하여 중지명령 등 필요한 조치를 임상시험책임자에게 할 수 있다.

(4) 임상시험책임자는 담당자로부터 중대하고 예상치 못한 이상약물반응 보고를 받은 경우 이상반응 보고서에 담당자로부터 제출 받은 보고서 사본을 첨부하여 사망을 초래하거나 생명을 위협하는 경우에는 임상시험책임자가 이 사실을 보고 받거나 알게 된 날로부터 7일 이내. 다만, 이 경우 상세한 정보를 최초 보고일로부터 8일 이내에 추가로 보고하여야 한다.

임상시험책임자는 추가적인 안전성 정보를 주기적으로 해당 이상약물반응이 종결(해당 이상약물반응의 소실 또는 추적조사의 불가능) 될 때까지 보고하여야 한다.
이상반응 발생시 즉시 임상시험책임자 및 담당자로부터 필요검사 및 치료를 받을 수 있도록 관리한다. 중대한 이상시술반응 발생시에는 임상시험책임자 또는 임상시험담당자는 시험을 중지 할 수 있으며 "이상반응 발생시 조치사항"에 따라 신속하고 적절한 조치를 취한다.

17. 자료 관리

본 임상시험의 자료관리는 ICH-GCP 및 KGCP 규정에 준하여 시행한다.

1) 증례기록서 기록 및 근거문서 확인(Source document verification)

근거문서(source document)에는 기록해야 할 자료가 발생할 때 즉시 기록한다. 만약 임상 종료시까지 기록되지 않은 경우 적절한 누락사유를 기록하여야 한다. 근거문서의 모든 수정사항은 먼저의 기록이 보이도록 한 줄로 그어 표시한 후 수정자료, 수정자, 수정사유, 수정일을 기록한다. 먼저의 기록이 보이지 않도록 하는 수정액 등을 사용하여서는 안 된다. 근거문서 작성이 완료된 대상자의 자료는 증례기록서에 입력한다. 모든 자료는 관련 정부기관, IRB 등의 요구에 의해 확인될 수 있도록 근거문서는 보관한다. 또한 시험자는 임상시험을 시작하기 전, 증례기록서 등 기타 적절한 곳에 정상범위나 참고치를 제시하여 자료의 확인(verification) 및 검증(validation)하는데 사용할 수 있도록 한다.

2) 증례기록서의 작성

시험책임자로부터 위임을 받은 증례기록서 작성자는 근거문서의 내용을 증례기록서에 정확하게 입력한다.

18. 대상자 동의서 양식

임상시험의 실시에 있어서 대상자에게 본 시험의 내용 및 임상시험용 의약품의 효과, 이상반응에 대해 사전에 충분히 설명한 후 대상자의 동의를 얻어 동의서를 작성하고 증례기록양식에 동의 취득연월일을 기재한다(대상자 동의서 양식 참조).

19. 피해자 보상에 대한규약

만일, 본 임상시험에 참여하는 대상자에게 예기치 않은 사고 혹은 피해 발생시 별첨 2의 대상자 보상에 대한 규약에 따라 적절한 보상을 한다(별첨 2. 피해자 보상에 대한 규약 참조).

20. 대상자의 안전 보호에 관한 대책

1) 스크리닝 검사를 통하여 대상자가 본 임상시험에 적절한지 엄격히 평가한다.

2) 임상시험계획서에 따라 임상시험을 실시하고 시험기간 중 정기적인 검사와 검진을 통하여 이상반응 및 이상약물반응의 출현 여부와 그 정도를 평가하고 적절한 조치를 취한다.
3) 채혈 시 감염방지를 위해 대상자들의 혈액채취는 일반인들의 출입이 통제된 방에서 실시하며, 사용하는 기구는 완전 멸균된 1 회용으로 한다.
4) 응급상황 발생 시 각 실시기관에서 정하고 있는 응급상황에 대한 대처방안에 준하여 조치한다.

21. 기타 임상시험을 안전하고 과학적으로 실시하기 위하여 필요한 사항

1) 임상시험관리기준(KGCP)

본 임상시험을 실시함에 있어 KGCP 및 Helsinki 선언의 근본 정신을 준수하여 윤리적이고 과학적인 배려 하에 연구를 실시하도록 한다.

2) 임상시험계획서의 준수 및 변경

본 임상시험은 IRB가 승인한 임상시험계획서(대상자 설명문 및 동의서 포함)에 따라 실시한다. 임상시험계획서의 모든 변경은 의뢰자와 시험책임자가 논의해야 한다. 시험자는 대상자 에게 위해가 발생되는 것을 즉각적으로 막기 위한 경우를 제외하고는 임상시험계획서 변경에 대하여 사전에 IRB로부터 승인을 얻어야 한다. 만일, 즉각적으로 대상자 에게 위해가 발생하지 못하게 하기 위하여 IRB의 승인을 얻기 전에 임상시험계획서의 변경을 적용하게 되는 경우, 가능한 한 빨리 이러한 변경에 대하여 IRB에 보고한다.

3) 대상자 동의

공고를 통하여 모집한 자원자를 대상으로, 임상시험담당자는 시험의 성격, 범위, 예상되는 결과 등에 대하여 이해하기 쉽도록 설명하고, 자원자로부터의 다양한 질문에 성실히 답변을 한다. 충분한 질의응답 시간을 가진 후, 준비된 동의서 양식에 대상자가 서명을 하도록 하며, 이러한 내용을 설명하고 동의서를 취득한 시험자도 서명을 한다(별첨 1. 대상자 모집 공고 참조, 대상자 동의서 양식 참조).

4) 비밀보장

모든 대상자의 신상에 대한 정보는 이니셜 혹은 기호 등으로 익명화하여야 하며, 임상시험으로부터 얻어진 결과에 대해서 관련된 모든 시험자는 비밀을 유지해야 한다. 또한 시험책임자는 서명을 받은 대상자 동의서를 보관하고, 대상자번호 및 대상자 명과 신원 확인에 필요한 자료가 기록된 리스트를 작성하여 나중에 기록을 찾을 수 있도록 하여야 한다.

5) 임상시험 모니터링

임상시험이 임상시험계획서와 KGCP 에 따라 실시되도록 임상시험 실시기관에서 경희대학교한방병원 한의약임상시험센터를 통하여 모니터링을 실시한다. 모니터링 시에는 증례기록이 완전하고 명확한지 근거문서와의 대조검토가 필요하다.

<참고문헌>

1. Engers, A.J.; Jellema, P.; Wensing, M.; Van Der Windt, D.A.; Grol, R.; Van Tulder, M.W. Individual patient education for low back pain. Cochrane Database Syst. Rev. 2008
2. Hoy, D.; March, L.; Brooks, P.; Biyth, F.; Woolf, A.; Bain, C.; Williams, G.; Smith, E.; Vos, T.; Barendregt, J.; et al. The global burden of low back pain: Estimates from the Global Burden of Disease 2010 study. Ann. Rheum. Dis. 2014, 73, 968–974.
3. Rubinstein, S.M.; van Middelkoop, M.; Assendelt, W.J.; de Boer, M.R.; van Tulder, M.W. Spinal manipulative therapy for chronic low-back pain. Cochrane Database Syst. Rev.
4. Kuijpers, T.; van Middelkoop, M.; Rubinstein, S.M.; Ostelo, R.; Verhagen, A.; Koes, B.W.; van Tulder, M.W. A systematic review on the effectiveness of pharmacological interventions for chronic non-specific low-back pain. Eur. Spine J. 2011, 20, 40–50
5. Manchikanti, L.; Boswell, M.V.; Singh, V.; Benyamin, R.M.; Fellows, B.; Abdi, S.; Buenaventura, R.M.; Conn, A.; Datta, S.; Derby, R.; et al. Comprehensive evidence-based guidelines for interventional techniques in the management of chronic spinal pain. Pain Physician 2009, 12, 699–802
6. Chou, R.; Deyo, R.; Friedly, J.; Skelly, A.; Hashimoto, R.; Weimer, M.; Fu, R.; Dana, T.; Kraegel, P.; Griffin, J.; et al. Nonpharmacologic Therapies for Low Back Pain: A Systematic Review for an American College of Physicians Clinical Practice Guideline. Ann. Int. Med. 2017, 166, 493–505.
7. Qaseem, A.; Witt, T.J.; McLean, R.M.; Forciea, M.A. Clinical Guidelines Committee of the American College of Physicians Noninvasive Treatments for Acute, Subacute, and Chronic Low Back Pain: A Clinical Practice Guideline From the American College of Physicians. Ann. Intern. Med. 2017, 166, 514–530.
8. Park, S.-Y., Hwang, E.-H., Cho, J.-H., Kim, K.-W., Ha, I.-H., Kim, M.-r., Nam, K., Lee, M. h., Lee, J.-H., Kim, N., & Shin, B.-C. (2020). Comparative effectiveness of Chuna manipulative therapy for non-acute lower back pain: A multi-center, pragmatic, randomized controlled trial. Journal of Clinical Medicine, 9(1), 144. https://doi.org/10.3390/jcm9010144
9. 신병철 외. 척추변위 명명체계에 대한 문헌고찰. 척추신경추나의학회지. 2011;6(1):141-8.
10. 이진현 외. 단순 방사선 영상 검사를 통한 추나의학적 진단방법 – 척추·골반변위 명명체계를 중심으로-, 척추신경추나의학회지. 2014;9(1):1-14.
11. 장준수. 합성곱 신경망을 이용한 요추 특징점 검출에 관한 연구. 차세대컨버전스정보서비스기술논문지. 2020;9(3):263-72.
12. Jin-hyun Lee, et al. Comparison of Concordance between Chuna Manual Therapy Diagnostic Methods (Palpation, X-ray, Artificial Intelligence Program) in Lumbar Spine: An Exploratory, Cross-Sectional Clinical Study. Diagnostics (Basel). 2022 Nov 8;12(11):2732. doi: 10.3390/diagnostics12112732.
13. Degenhardt BF, Snider KT, Snider EJ, Johnson JC. Interobserver reliability of osteopathic palpatory diagnostic tests of the lumbar spine: improvements from consensus training. J Am Osteopath Assoc. 2005;105(10):465-73
14. Deed E. Harrison, Donald D. Harrison, Christopher J. Colloca, Joseph Betz, Tadeusz J. Janik P, Burt Holland. Repeatability Over Time of Posture, Radiograph Positioning, and Radiograph Line Drawing: An Analysis of Six Control Groups. J Manipulative Physiol Ther 2003;26:87-98.
15. Lee, J.H.; Choi, M.h.; Kim, J.I.; Jang, J.S.; Park, T.Y. Radiograph-based Diagnostic Methods for Thoracic and Lumbar Spine Malposition in Chuna Manual Therapy Using Biomarkers. The Journal of Churna Manual Medicine for Spine and Nerves 2023, 18, 1-8.
16. 주민수. A Study on the Diagnosis of Spinal Malposition in Chuna Manual Therapy Using X-ray Images Based on Digital Markers. 우석대학교 일반대학원. 박사학위 논문. 2025
17. 한국보건의료원. 의료기술재평가보고서 2024 요통 환자에서의 간섭파 전류치료. 2024.05.31.
18. 전창훈, 김동재, 김동준, 이환모, 박희천. 한국어판 Oswestry Disability Index(장애지수)의 문화적 개착. 대한척추외과학회지. 2005;12(2):146-52.
19. Kim Tae-Hyup. Validity and reliability evaluation for EQ-5D in the general population of south Korea [Doctorate thesis]. Ulsan : University of Ulsan the Graduate School. 2012. Korean Language.
20. Lee JS, Lee DH, Suh KT, Kim JI, Lim JM, Goh TS. Validation of the Korean version of the Roland-Morris Disability Questionnaire. Eur Spine J. 2011;20(12):2115-9
21. Childs JD, Piva SR, Fritz JM. Responsiveness of the numeric pain rating scale in patients with low back pain. Spine (Phila Pa 1976). 2005 Jun 1;30(11):1331-4. doi: 10.1097/01.brs.0000164099.92112.29. PMID: 15928561.
