# Supplementary material for: The effectiveness of Chuna manual therapy based on radiographic malposition diagnosis in patients with non-acute low back pain: A study protocol for a randomized, assessor-blind, parallel-group, controlled trial
Source: PLoS One. 2026 May 11;21(5):e0347321. doi: 10.1371/journal.pone.0347321 (PMC13160342; doi:10.1371/journal.pone.0347321)
Supplement: S3 Appendix — (PDF) [file pone.0347321.s004.pdf]

1. Title of the clinical trial

The Effectiveness of Chuna Manual Therapy Based on Radiographic Malposition Diagnosis in Patients with Non-acute Low Back Pain (a randomized controlled, two-arm, parallel study, assessor-blind)

---

2. Names and addresses of the trial institutions

- **Coordinating institution for the clinical trial**
  Catholic Kwandong University International St. Mary's Hospital 25, Simkok-ro 100-gil, Seo-gu, Incheon, Republic of Korea
- **Participating trial institutions**
  1. Catholic Kwandong University International St. Mary's Hospital 25, Simkok-ro 100-gil, Seo-gu, Incheon, Republic of Korea
  2. Wonkwang University Korean Medicine Hospital 895, Muwang-ro, Iksan-si, Jeollabuk-do, Republic of Korea

---

3. Sponsor of the clinical trial

- **Sponsor**: Ministry of Health and Welfare
- **Implementing agency**: Korea Health Industry Development Institute

4. Objectives and background of the clinical trial

4.1 Objectives

This clinical trial aims to explore the **therapeutic effects of Chuna manual medicine based on radiographic malposition diagnosis** in patients with non-acute low back pain.

4.2 Background

4.2.1 Low back pain

- Low back pain is one of the most common musculoskeletal disorders worldwide; 70–80% of people experience it at least once in their lifetime.
- In many countries, low back pain is a leading cause of disability and medical expenditure, imposing a great socioeconomic burden.
- In particular, **non-acute** (subacute or chronic) low back pain often shows a recurrent or persistent course and tends to become chronic, requiring long-term management.

4.2.2 Usual non-invasive treatments for low back pain

- Recent clinical practice guidelines on non-invasive treatment of low back pain recommend various options such as pharmacologic therapy, psychological therapy, physical therapy, exercise, manual therapy, and patient education.
- Among these, **spinal manual therapy** has been recommended with low-moderate levels of evidence as a treatment option for acute, subacute, and chronic low back pain.

4.2.3 Chuna manual therapy

- **Chuna manual therapy (CMT)** is a Korean manual treatment performed by Korean medicine doctors that includes techniques aimed at restoring balance between anatomical structures and their functions.
- Since 2019, Chuna manual therapy has been covered by the Korean National Health Insurance for musculoskeletal disorders, and various studies have verified its efficacy and safety.
- Diagnostic procedures performed before Chuna treatment can broadly be divided into:
  1. **Manual palpation-based diagnosis**, and
  2. **Instrument-assisted diagnosis** using imaging devices (radiological imaging) or posture-analysis devices.
- However, there is still a serious lack of data on:
  - The objectivity of each diagnostic method,
  - Inter- and intra-rater reliability, and
  - Analyses of treatment outcomes according to diagnostic results.

4.2.4 Previous studies on imaging-based diagnostic methods for Chuna medicine

(1) Domestic clinical studies

(a) Overview

- To date, diagnostic methods for Chuna based on radiological data include:
  - Establishing a diagnostic system,
  - Developing an AI-based diagnostic program using vertebral landmarks, and

    - Clinical studies assessing concordance of Chuna diagnoses and applying AI programs.
- Chuna radiographic diagnosis performed on the basis of a standard operating procedure (SOP) developed through expert consensus has been shown to demonstrate **higher inter-rater agreement** than palpation-based diagnostic methods.

(b) A Summary Study on the Composition of a Diagnostic Method for Chuna Based on Plain X-ray Images

1. **Stage 1 – Establishment of a diagnostic system (2011–2014)**
    - *Literature review on nomenclature systems for spinal malposition*
        - Classified and presented nomenclature systems for Chuna-based spinal malposition.
    - *Development of a diagnostic method for Chuna medicine using plain radiography of the spine.*
        - Performed Chuna malposition diagnosis by comprehensively considering bony structures visible on radiographs.
2. **Stage 2 – Development of an AI-assisted diagnostic program (2015–2017)**
    - *Development of an AI program based on vertebral body landmarks on plain radiographs*
        - Constructed an AI model that identifies vertebral landmarks on lumbar radiographs and outputs malposition patterns corresponding to Chuna diagnostic categories.
3. **Stage 3 – Clinical studies on diagnostic concordance and application of AI (2017–2020)**
    - *Study on concordance between different diagnostic methods (palpation, radiographic diagnosis, AI program)*
        - Reported that radiographic diagnosis showed higher inter-rater reliability than palpation-based diagnosis, and that the AI program achieved a level of diagnostic agreement similar to that of Chuna experts.
    - *Application of the AI program in clinical practice*
        - Demonstrated the feasibility of applying the AI-based diagnostic program in real-world clinical settings.

(2) Overseas research related to AI-based vertebral position analysis

- Overseas studies on AI programs for vertebral position analysis have mainly focused on **evaluation of scoliosis** (e.g., Cobb angle measurement) rather than on assessing vertebral alignment and malposition from the perspective of manual medicine.
- Studies that diagnose vertebral malposition patterns in a way directly applicable to manual medicine techniques are largely lacking.

(3) Key previous work

1. Chuna diagnostic method using plain radiography (2014)
   - Presented a new diagnostic approach using plain radiographs as a way to compensate for limitations of manual Chuna diagnosis and to ensure objectivity and reproducibility.
   - Conducted Chuna malposition diagnosis by comprehensively evaluating bony structures on plain films.
   - Based on these findings, standard operating procedures (SOPs) for classification of malposition patterns and corresponding Chuna treatment methods were developed.
2. Development and validation of AI-assisted vertebral malposition diagnosis
   - Used vertebral body landmarks extracted from lumbar radiographs to train AI models that output malposition categories used in Chuna medicine.
   - Reported that the AI-based method shows acceptable agreement with expert Chuna diagnoses and has potential for use as a clinical decision-support tool.
   - Jin-hyun Lee, et al. Comparison of Concordance between Chuna Manual Therapy Diagnostic Methods (Palpation, X-ray, Artificial Intelligence Program) in Lumbar Spine: An Exploratory, Cross-Sectional Clinical Study. Diagnostics (Basel). 2022 Nov 8;12(11):2732. doi: 10.3390/diagnostics12112732.)

Rationale and strengths of this study

◯ Need for a study evaluating the effectiveness of applying Chuna interventions based on radiological assessment

- To date, previous studies on radiological assessment in Chuna medicine have mainly taken the form of retrospective studies, such as conceptual overviews, establishment of diagnostic landmarks, and evaluation of inter-observer agreement between different diagnostic methods. However, no study has actually evaluated, in real clinical practice, the effectiveness of a Chuna treatment approach that is guided by radiological data–based Chuna diagnostic methods.
- Therefore, a clinical trial is needed in which standardized Chuna treatment methods are applied according to displacement (malposition) patterns assessed on imaging, in order to verify their clinical effectiveness.

◯ Application of interventions with demonstrated efficacy and safety

- By adopting a pragmatic study design, we set a usual-care control group according to previous studies and clinical guidelines, and apply Chuna therapy to patients with conditions for which safety and effectiveness have already

been demonstrated in prior research. This allows the clinical trial to be conducted efficiently in a real-world clinical context.

○ Provision of relevant evidence for clinical practice in Korean medicine

- Chuna imaging diagnosis based on radiological data, together with extraction of imaging biomarkers, can serve as an objective basis for comparing and evaluating the effects of Chuna treatment before and after the intervention. This will enable the accumulation of more rational and objective clinical data for future Chuna-related clinical research.
- The development of biomarkers and diagnostic techniques for rational Chuna diagnosis can be used as foundational research to support future insurance coverage (reimbursement) of Chuna diagnostic procedures.

5. Description of the interventions

5.1 Radiograph-based Chuna diagnostic method used in this clinical trial

5.1.1 Radiographic acquisition method

For evaluation of lumbar spinal segment alignment, we will use the radiographic positions that were employed in previous studies that verified the reproducibility of lumbar alignment measurements. These radiographic procedures follow the standardized protocols of Chiropractic Biophysics (CBP) and were designed to increase the reproducibility and reliability of radiographic acquisition.

(1) Anteroposterior (AP) view

- The participant stands barefoot, and the midline of the pelvis is aligned with the central beam of the X-ray unit.
- The pelvic mid-sagittal plane is aligned to the central beam, and the buttocks are centered relative to the detector.
- The participant is instructed to nod the head forward and backward twice and then assume a neutral head position.
- The participant then closes the eyes, opens them, and looks straight ahead naturally.
- Abnormal pelvic posture (for example, lateral tilt) is not artificially corrected, so that the image reflects the participant's habitual alignment.
- The X-ray beam is centered at the L3 level, with a standard source-to-image distance of 101.6 cm (40 inches), and is generally kept horizontal.

(2) Lateral view

- The shoulders and pelvis are positioned perpendicular to the X-ray beam.
- The participant moves the head twice to find a neutral position.

- The arms are crossed in front of the chest to minimize changes in pelvic or thoracic posture.
- Abnormal lumbar posture is not corrected, so that the natural alignment is reflected.
- The X-ray beam is directed toward the L4 level from a distance of 101.6 cm (40 inches).
- These positions are designed to allow accurate measurement of pelvic axis, lumbar curvature, and segmental inclination of the lumbar vertebrae.

5.1.2 Chuna diagnostic method for spinal malposition

- Using DICOM files of the acquired lumbar radiographs, we apply the digital markers proposed in previous studies and derive the flexion, lateral bending, and rotation angles for each vertebral body.
- A DICOM labeling program (Dicomlabel, Korea Institute of Oriental Medicine, Daejeon, Korea) is used to generate the segmental angles.

(1) Generation of digital markers

- Lateral view
  - For each lumbar vertebral body, the anterior and posterior end points of the superior endplate are connected to form the superior line, and the anterior and posterior end points of the inferior endplate are connected to form the inferior line.
  - To diagnose malposition of L5, reference points on the sacrum are also required. Therefore, digital markers are created using two endplate end points on S1, the line connecting them, and additional sacral landmarks as specified in the standard operating procedure.
- AP view
  - For each lumbar vertebral body, the left and right end points of the superior endplate are connected to form the superior line, and the left and right end points of the inferior endplate are connected to form the inferior line.
  - In addition, the most medial points of the bilateral pedicles are identified as additional landmarks.
  - For diagnosis of L5 malposition, digital markers are generated using the left and right end points of the superior surface of S1 and the line connecting them, the most lateral points of the sacral ala, and the midpoint of the S2 spinous process.

(2) Determination of malposition based on angular values

- Based on the angles derived by the program, the presence or absence of malposition is determined.

- Using the previously derived threshold values for malposition diagnosis (cut-off angles), segments with angles equal to or exceeding the threshold are classified as having Chuna-defined vertebral malposition (for example, if the flexion angle of L1 is 3°, it is diagnosed as a flexion malposition).
- For rotational malposition of L5, there is no separate segment below L5. Therefore, the value is used only when the left–right distance difference between the sacral ala and the S2 tubercle falls within the normal range specified by the program; otherwise, the value is recorded in the case report form but treated as missing in malposition diagnosis and statistical analysis.
- The reference threshold angles for each lumbar vertebral body are as follows

	Vertebral body				
	L1	L2	L3	L4	L5
Flexion(°)	0.64	3.56	5.29	8.90	8.53
Extension(°)	10.46	14.95	17.61	20.76	24.47
Lateral bending(°)	2.21	2.10	1.92	2.06	2.31
Rotation(°)	9.49	5.19	4.59	5.87	7.13

5.2 Treatment procedures used in this clinical trial

5.2.1 Usual care (common to both groups): physiotherapy and patient education

Usual care consists of physiotherapy and education, provided twice per week for 4 weeks, for a total of 8 sessions.

(1) Physiotherapy (Interferential Current Therapy, ICT)

- Two pairs of electrodes (four pads) are attached in a crossed pattern centered on the area where pain is most severe, so that the interferential current focuses on the painful region.
- A medium-frequency alternating current of 4,000–4,100 Hz is applied for 15 minutes, and the intensity is adjusted according to the patient's subjective sensation so that pain is not felt.
- Treatment is administered twice per week for 4 weeks, for a total of 8 sessions.
- Devices used:
    - Catholic Kwandong University International St. Mary's Hospital, Department of Korean Medicine: STRATEC STT-570 (STRATEC Co., Ltd., Korea)
    - Wonkwang University Korean Medicine Hospital: EF-160 (OG Giken Co., Japan)

(2) Exercise guidance for patients

- At each visit, approximately 15 minutes of exercise instruction is provided, including: pelvic tilt exercises, trunk rotation without weight bearing, knee-to-chest exercises, partial curl-ups, and other exercises appropriate for low back pain.
- Patients are instructed in how to perform these exercises safely at home.

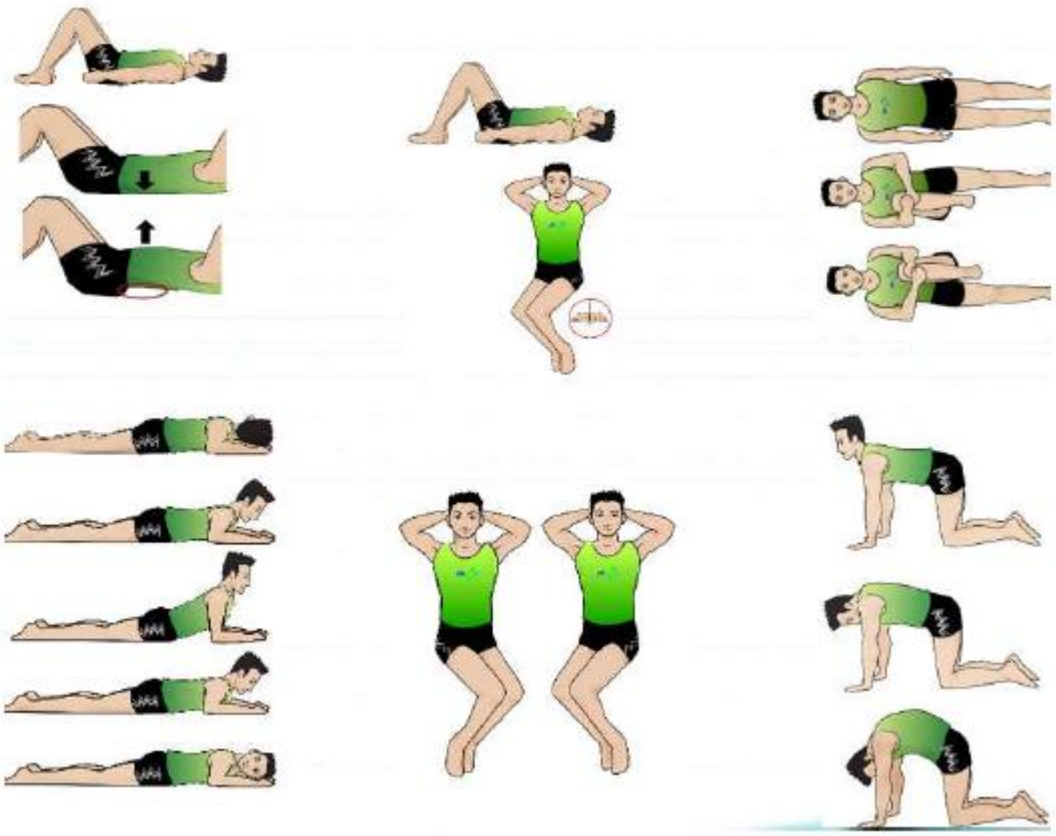

(3) Patient education

- Educational materials and explanations are provided regarding postures that help prevent low back pain and appropriate working postures and ergonomics.
- Education focuses on modifying daily activities and occupational tasks to reduce mechanical stress on the lumbar spine.

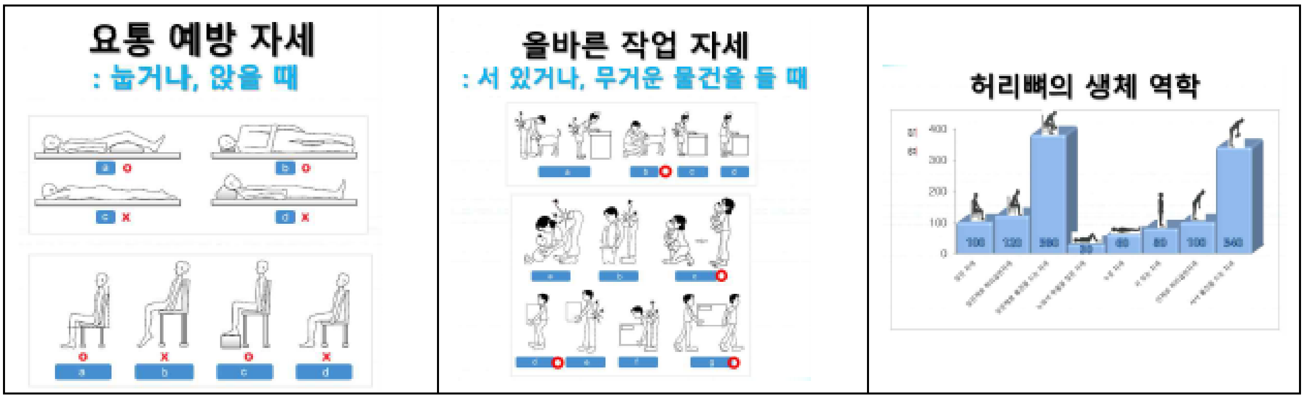

5.2.2 Chuna manual therapy (applied only in the Chuna treatment group)

(1) Application of Chuna therapy according to radiograph-based diagnostic results

- In the Chuna treatment group, Chuna manual therapy is performed based on the Chuna diagnostic results derived from the plain radiographs.
- Treatment is provided twice per week for 4 weeks (approximately 15 minutes per session), for a total of 8 sessions.
- According to the diagnostic results, the standardized operating procedures (SOPs) specified in the protocol are used to select the treatment method.
- Based on the classification of Chuna diagnostic results into simple and complex malposition patterns, Chuna treatment methods specified in the SOP are applied to the corresponding vertebral levels.
- When more than one corrective technique is presented in the SOP for a given malposition, the practitioner may choose the most appropriate technique according to clinical judgement.
- If, in the clinical judgement of the practitioner, additional Chuna therapy is required for regions other than the lumbar spine (for example, the pelvis), such techniques may be applied, and these procedures will be recorded in the case report form.
- However, for the lumbar spine itself, no Chuna techniques other than those specified in the SOP may be applied.

(2) Determination of the exact treatment level using ultrasound prior to Chuna therapy

- To ensure accurate contact at the treatment level corresponding to the malposition identified on plain radiographs, ultrasound imaging is used to confirm the vertebral level before Chuna therapy.
- Using a longitudinal view, the shape of the spinous processes is visualized, and the levels are identified sequentially from S1 upwards.
- Once the target level is confirmed, the skin overlying the planned treatment site is marked.
- Chuna techniques are then performed at the marked level, following the standardized procedures described in the SOP.

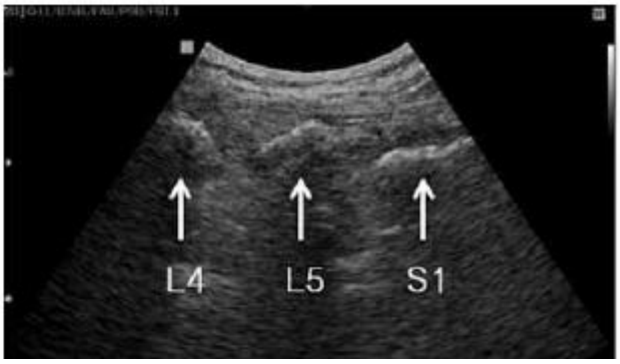

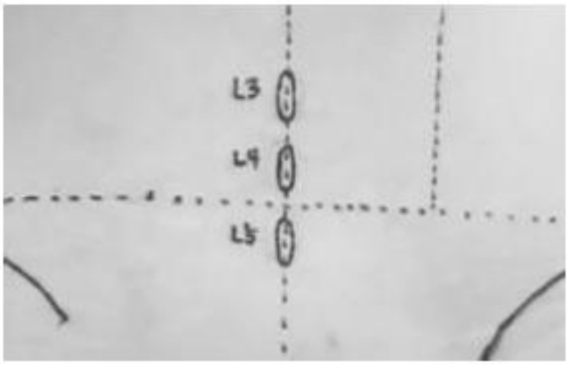

◯ Lumbar Chuna techniques used in this trial

In this clinical trial, standardized lumbar Chuna manual therapy techniques are applied according to the radiograph-based Chuna diagnostic results.
The main lumbar techniques used in the study are as follows.

① Simple Chuna techniques for simple malposition

For patients in whom a single simple malposition pattern is identified (bilateral flexion, bilateral extension, rotation, or lateral bending), the corresponding "simple" lumbar Chuna technique is applied as the primary corrective procedure.

- Bilateral flexion malposition – seated lumbar extension correction technique
    - *Patient position*: Seated.
    - *Physician position*: Standing behind the patient.
    - *Contact and hand position*:
      · The treating hand (ju-dong-su) contacts the spinous process of the lower vertebral segment with the thenar/hypothenar region.
      · The other hand wraps around and holds the patient's opposite shoulder and upper arm to fix the upper trunk.
    - *Corrective direction*:
      · With the upper body fixed, the physician extends the patient's lumbar spine by guiding the trunk backward over the contact point on the lower segment, thereby correcting the bilateral flexion malposition into extension.
- Bilateral extension malposition – seated lumbar flexion correction technique
    - *Patient position*: Seated.
    - *Physician position*: Standing beside or slightly behind the patient.
    - *Contact and hand position*:
      · The treating hand contacts the spinous process of the lower segment.
      · The other hand supports the patient's upper trunk and shoulders from the front.
    - *Corrective direction*:
      · The physician flexes the patient's trunk forward over the contact point on the lower segment, applying a controlled flexion force to correct the bilateral extension malposition.
      · The patient is instructed to gently resist by attempting to straighten the trunk, while the physician applies counter-pressure, thereby performing an active-assisted correction.
- Rotational malposition – side-lying lumbar traction technique with rotation
    - *Patient position*: Side-lying, with the side of rotation directed downward.
    - *Physician position*: Standing in front of the patient, in a fencing-like stance.
    - *Contact and limb positioning*:
      · The lower leg is flexed, and the foot of the upper leg is placed in the

popliteal fossa of the lower leg.
· The caudal (foot-side) hand grasps the popliteal area of the upper leg, while the cranial (head-side) hand contacts the anterior aspect of the upper shoulder.
    - *Corrective direction*:
    · By simultaneously applying downward pressure through both hands, the physician produces rotational traction of the lumbar segment toward the corrective direction, thereby correcting the rotational malposition.
- **Lateral bending malposition – lumbar flexion-distraction technique with lateral bending**
    - *Patient position*: Prone on a flexion-distraction (Cox) table. The feet are secured with a belt.
    - *Physician position*: Standing at the side of the pelvic section of the table.
    - *Contact and hand position*:
    · The treating hand contacts the spinous process of the vertebral segment to be treated, using the thenar/hypothenar region.
    · The other hand operates the handle of the Cox table.
    - *Corrective direction*:
    · While maintaining firm contact on the spinous process, the physician moves the pelvic section of the table to the left or right using the table handle, producing flexion-distraction combined with lateral bending at the target segment and correcting the lateral bending malposition.

② **Specialized Chuna techniques for simple malposition**

For patients with a **single simple malposition pattern** (bilateral flexion, bilateral extension, rotation, or lateral bending), more localized *segmental* techniques are used in addition to the simple techniques. These are applied when a stronger, more focused correction is required at a specific motion segment.

- **Bilateral flexion malposition – prone or side-lying lumbar extension thrust (spinous-process thrust technique)**
    - *Patient position*: Prone or side-lying, with the target segment positioned near the edge of the table.
    - *Physician position*: Standing in a fencing stance at the side of the table.
    - *Contact*: The main (foot-side) hand uses the small thenar/pisiform region to contact the spinous process of the upper vertebra at the segment to be corrected. The assisting hand supports and stabilizes the patient's upper body or head.
    - *Correction*: With the patient's trunk slightly flexed or neutral, the physician applies a quick, controlled downward and anterior thrust through the contact point to induce extension at the flexed segment and correct the bilateral flexion malposition.
- **Rotational malposition – prone "small-thenar-spinous/mammillary process" thrust technique**

- *Patient position*: Prone, with the head supported and the lumbar region centered on the table.
- *Physician position*: Standing on the side opposite to the direction of rotation, in a fencing stance.
- *Contact*:
  · For spinous-process contact: the small thenar/pisiform region of the main hand contacts the spinous process of the rotated vertebra.
  · For mammillary-process contact: the small thenar/pisiform region contacts the mammillary process on the side opposite to rotation.
- *Correction*: The physician applies a short, high-velocity thrust in the direction opposite to the malposition (toward derotation), while stabilizing the trunk with the assisting hand. This "resisted thrust" technique is used when a more distinct rotational correction is needed.

- **Lateral bending malposition – opened-wedge / closed-wedge mammillary-process thrust techniques**
  - *Patient position*: Side-lying on the flexion-distraction (Cox) or standard table, with the upper and lower legs positioned to create either an opened wedge or a closed wedge at the target segment.
  - *Physician position*: Standing in a fencing stance at the side of the pelvis.
  - *Contact*: The main hand uses the small thenar/pisiform region to contact the mammillary process of the target segment, while the assisting hand controls the patient's shoulder or pelvis.
  - *Correction*:
    · **Opened-wedge approach**: The pelvic section of the table and the patient's legs are positioned to open the wedge on the concave side, then a thrust is delivered toward the opened wedge to correct lateral bending.
    · **Closed-wedge approach**: The pelvic section and lower extremities are positioned to close the wedge on the convex side, and a thrust is delivered toward that side to restore balanced alignment.

---

③ Simple Chuna techniques for complex malposition

For **complex malposition patterns** (e.g. extension + rotation + ipsilateral lateral bending; flexion + rotation + ipsilateral lateral bending; lateral bending with contralateral rotation), *simple multi-planar correction techniques* are applied first. These techniques combine flexion/extension, rotation, and lateral bending in a single maneuver, using leverage through the trunk and lower extremities.

- **Extension + rotation + ipsilateral lateral bending malposition – side-lying lumbar correction technique**

    - *Patient position*: Side-lying with the involved side downward. The lower leg is extended, and the upper leg is placed over the lower leg or positioned to create slight lateral bending at the target segment.
    - *Physician position*: Standing in front of the patient in a fencing stance.
    - *Contact*: The physician's forearm (assisting arm) contacts the anterior shoulder of the patient, and the other hand or forearm controls the lower extremities (usually the ankle or distal leg of the lower limb).
    - *Correction*:
      · The trunk is flexed to the level of the involved segment, and rotation is induced by pushing the shoulder backward while monitoring motion at the target level.
      · Simultaneously, the lower extremities are lifted upward to create lateral bending in the opposite direction, thereby correcting the combined extension–rotation–lateral bending pattern with a smooth, non-thrust mobilization.
- Flexion + rotation + ipsilateral lateral bending malposition – side-lying lumbar correction technique
    - *Patient position*: Side-lying with the involved side downward, similar to the above technique.
    - *Physician position*: Standing in a fencing stance.
    - *Contact*: As above, the assisting forearm stabilizes the shoulder, and the other hand controls the lower extremities.
    - *Correction*:
      · The trunk is extended from a flexed position while rotation is induced by the forearm on the shoulder.
      · The legs are elevated or lowered to generate corrective lateral bending, and the physician monitors motion at the target segment during the maneuver.
- Lateral bending with contralateral rotation – seated or flexion–distraction–based correction
    - *Patient position*: Seated or prone on a flexion–distraction table, depending on the segmental pattern.
    - *Physician position*: Standing behind or beside the patient.
    - *Contact*: One hand stabilizes the pelvis or lower trunk, while the other guides the thorax or shoulders.
    - *Correction*: The physician combines flexion or extension with rotation and opposite-side lateral bending of the trunk, using continuous mobilization rather than thrust, to reduce the complex pattern.

---

④ Specialized Chuna techniques for complex malposition

- Extension + rotation + ipsilateral lateral bending malposition – side-lying trunk lever thrust technique

  - *Patient position*: Side-lying with the involved side downward.
  - *Physician position*: Standing in a fencing stance in front of the patient.
  - *Contact*:
    · Main hand (foot-side hand): the forearm contacts the iliac crest of the pelvis on the lower side.
    · Assisting hand (head-side hand): the forearm contacts the axillary region of the upper shoulder and supports the trunk.
  - *Correction*:
    · The trunk is rotated toward the correction direction by guiding the shoulder with the assisting forearm, while the main forearm on the ilium induces posterior rotation of the pelvis relative to the trunk.
    · After preloading the tissues and confirming motion at the involved segment, the physician delivers a quick thrust using body weight, pulling the ilium anterosuperiorly or anteroinferiorly (depending on the pattern) to correct the extension–rotation–lateral bending malposition.
- Flexion + rotation + ipsilateral lateral bending malposition – side-lying trunk lever thrust technique
  - *Patient position*: Side-lying, with leg and trunk positioning adjusted to emphasize flexion at the target segment.
  - *Physician position*: As above (fencing stance in front of the patient).
  - *Contact*: Main forearm on the iliac crest; assisting forearm at the axilla.
  - *Correction*:
    · The trunk is rotated and flexed to localize motion at the involved segment.
    · The physician then applies a rapid thrust through the ilium using the main forearm while stabilizing the trunk with the assisting forearm, thereby correcting the flexion–rotation–lateral bending malposition.
- Lateral bending with contralateral rotation malposition – side-lying trunk lever thrust technique
  - *Patient position*: Side-lying, with the lower trunk slightly flexed or extended and the legs positioned to emphasize the lateral bending component.
  - *Physician position*: Standing in front of the patient in a fencing stance.
  - *Contact*:
    · Main forearm on the iliac crest of the lower side.
    · Assisting forearm at the axillary region of the upper side.
  - *Correction*:
    · The assisting forearm induces controlled rotation of the trunk, while the main forearm applies a thrust through the pelvis in the opposite direction of the malposition.
    · Using body weight, the physician performs a momentary thrust at the end range of combined motion to correct the lateral bending + contralateral rotation pattern.
-

6. Inclusion criteria, exclusion criteria, target number of participants, and rationale

The participants in this clinical trial will be patients with non-acute low back pain of at least 3 weeks' duration who are selected for the study according to the following inclusion and exclusion criteria.

1) Inclusion criteria (Inclusion Criteria)

① Age: 19 years or older and 70 years or younger at the time of consent.
② Patients with non-acute low back pain (symptom duration ≥ 3 weeks) whose average low back pain intensity during the past week is Numeric Rating Scale (NRS) ≥ 4.
③ Patients in whom Chuna spinal malposition has been confirmed on plain radiographs.
④ Patients who voluntarily agree to participate in the clinical trial and sign the written informed consent form.

---

2) Exclusion criteria (Exclusion Criteria)

① Patients diagnosed with serious specific diseases that can be the cause of low back pain (e.g., spinal metastasis of tumors, acute fracture and spinal dislocation, scoliosis of moderate or greater severity).

② Patients with structural abnormalities of the lumbar spine (sacralization, lumbarization), those with a prior history of osteoporosis, or those in whom fracture of the lumbar spine is suspected.

③ Patients who are within 3 months after lumbar spine surgery.

④ Patients with other chronic diseases that may interfere with the treatment effect or with interpretation of the study results (e.g., chronic renal failure), as judged by the investigator.

⑤ Patients with progressive neurological deficits or serious neurological symptoms such as cauda equina syndrome.

⑥ Patients who have internal fixation or stabilizing devices in the lumbar spine due to previous lumbar surgery.

⑦ Patients with contraindications to interferential current therapy (ICT), such as cancer-related pain or an implanted pacemaker.

⑧ Patients who are currently taking systemic steroids, immunosuppressants, psychiatric medications, or other drugs that may affect the study results, as judged by the investigator.

⑨ Patients who have received Chuna therapy within the past week, or who have received strong opioid analgesics, anesthetic agents, or invasive treatments such as acupuncture or injection therapy during that period.

⑩ Patients who have received nerve block procedures to the lumbar region within the past 2 months.

⑪ Patients who are pregnant, breastfeeding, or planning pregnancy during the study period.

⑫ Patients who are scheduled, during the 4-week treatment period, to undergo surgery, procedures, or pharmacologic treatment that may affect evaluation of the study results, without this being under the direction of the study physician.

⑬ Any other individuals whom the investigator judges to be inappropriate for participation in the clinical trial.

---

3) Number of participants and rationale

(1) First pilot clinical study

- Total number of participants: 46 (multicenter recruitment)
  · Catholic Kwandong University International St. Mary's Hospital: 12 participants
  · Wonkwang University Korean Medicine Hospital: 34 participants
- Planned number of participants and interventions by group

Group	Planned number of participants	Planned interventions
I	23	Usual care (ICT + exercise guidance) + Chuna manual therapy
II	23	Usual care (ICT + exercise guidance)

(2) Rationale for the number of participants in the pilot clinical study

☐ Analysis of previous studies
The sample size for this study was calculated based on the results reported in a previous similar clinical study, using **Cohen's effect size.**

In the previous study, the change in low back pain NRS score in the **Chuna + usual care group** was **3.02 ± 1.72**, whereas in the **usual care group** it was **1.36 ± 1.75** ($p < 0.001$). Based on these data, the effect size (Cohen's d) was calculated to be **d = 0.96**.

Assuming a two-tailed test, significance level $\alpha = 0.05$, and statistical power $(1 - \beta) = 80\%$, the following formula was used:

$$n = 2 \times [(Z_{\alpha/2} + Z_{\beta})^2 / d^2]$$

Here, $Z_{\alpha/2}$ is the Z value for a two-sided test at significance level 0.05 (1.96), and Z_{β} is the Z value for 80% power (0.84).

The calculation indicated that the **minimum required sample size per group is 18 participants**.

Taking into account an anticipated **20% dropout rate** during the study, the final target sample size was set at **23 participants per group**, resulting in a total of **46 participants** to be enrolled.

6. Study period

- Study period: **From the IRB approval date to 31 December 2026**.

7. Study methods

- **Total number of participants:** 23 participants × 2 intervention groups = **46 participants** in total.
- **Group-specific planned number of participants and interventions**

Group	Planned number of participants	Planned interventions
I	23	Usual care (ICT + exercise + lifestyle education) + Chuna manual therapy
II	23	Usual care (ICT + exercise + lifestyle education)

<Study Flow chart>

A schematic diagram of the study flow is provided in the **Study Flowchart (Figure)**.

Enrollment

- Decision to participate in the clinical trial
- Screening test
- Randomization (n = 46) *(0 weeks)*

Allocation

- Usual care + Chuna manual therapy (n = 23)
- Usual care (n = 23)

Treatment period
(2 times/week × 4 weeks)

1. Usual care
   - Interferential current therapy (ICT) + exercise guidance
2. Chuna manual therapy
   - Chuna manual therapy based on malposition diagnosis

Follow-up

- 8 weeks

Analysis / Follow-up

- Data collection and trial outcomes assessment

1) Assignment of subject numbers (Randomization)

- For individuals who have provided **written informed consent** to participate in the clinical trial, a **screening number** is assigned in the order of outpatient visits at screening, as follows:
  · Catholic Kwandong University International St. Mary's Hospital: CK-S001, CK-S002, …
  · Wonkwang University Korean Medicine Hospital: WK-S001, WK-S002, …
- After the screening tests, participants who meet the selection criteria and do not meet any exclusion criteria are finalized as study subjects.
- After subject selection, **one person among the research personnel** who is not involved in treatment or assessment and **does not pose any risk of influencing the study results or their analysis** is designated as **unblinded**.
- This unblinded person opens, in order, the **opaque allocation envelopes** prepared based on a **block randomization list** generated by an independent statistician, and assigns a **randomization number** to each participant as follows:
  · Catholic Kwandong University International St. Mary's Hospital: CK-R001, CK-R002, …
  · Wonkwang University Korean Medicine Hospital: WK-R001, WK-R002, …
- A single participant cannot be assigned **more than one** randomization number, and each randomization number is assigned to **only one** participant.
- For each participant, the combination of the screening number, randomization number, and initials is used as the **subject identification code** to identify the participant until the end of the clinical trial.

- The randomization list allocates participants to the **usual care + Chuna group (Group I)** and the **usual care-only group (Group II)** in a **1:1 ratio**. The randomization table is generated by an independent professional statistician, and the randomization envelopes are also prepared by a third party not involved in the study.

2) Blinding and unblinding

Due to the nature of the treatment interventions and study design in this clinical trial, it is not feasible to blind the clinical investigators and participants to group allocation. Instead, outcome assessors will remain blinded in order to minimize bias in the evaluation of participants. Because the clinical investigators and participants are not blinded, no specific unblinding procedure is planned.

3) Outline of treatments in each group (summary)

- **Usual care (both groups)**
  · Interferential Current Therapy (ICT) applied to the painful lumbar region for **15 minutes per session**,
  · **Exercise guidance** (pelvic tilt, trunk rotation without weight bearing, knee-to-chest, partial curl-ups, etc.) for about **15 minutes** at each visit,
  · **Lifestyle education** and patient education materials on postures that help prevent low back pain and appropriate working postures.
  → Provided **twice per week for 4 weeks (total 8 sessions)** in both groups.
- **Chuna manual therapy (Group I only)**
  · In addition to usual care, standardized Chuna manual therapy is applied according to the **radiograph-based Chuna malposition diagnosis** confirmed at screening.
  · Techniques are selected according to the SOP for each malposition pattern (simple malposition, specialized techniques for simple malposition, simple techniques for complex malposition, specialized techniques for complex malposition).
  · Chuna therapy is provided **twice per week for 4 weeks (total 8 sessions, about 15 minutes per session)**.
  · Whenever necessary, ultrasound is used before treatment to confirm the exact vertebral level and mark the skin contact point.

4) Permitted concomitant medications and precautions

In principle, during the clinical trial period, no medications other than the trial interventions provided within this study should be administered. However, because this is designed as a pragmatic clinical trial, **rescue medications** may be administered at the discretion of the principal investigator when they are deemed necessary for the management of adverse events, pain control, and similar clinical needs.

If a concomitantly administered medication is expected to affect the pharmacodynamic evaluation or safety assessment of this trial, the corresponding participant will be withdrawn from the study (for permitted rescue medications, see **Section 13. Rescue medication**).

All concomitant medications and the reasons for their use must be documented in the source documents and the case report form (CRF), and signed by the principal investigator or sub-investigator. For any other medications, participants must be instructed to **consult the study team before taking the medication**.

---

8. Observation and examination items

1) Screening visit (Visit 1, Day −7 to Day 0)

Screening to assess eligibility will be carried out **only in participants who have signed the written informed consent form**. Participants in whom clinically significant abnormalities are identified on the following assessments will be excluded.

(If eligibility is confirmed at the screening visit, the baseline tests may be conducted on the same day.)

(1) **Demographic information**

- Sex, age, height, weight, BMI, smoking history, alcohol consumption.

(2) **Medical history**

- Duration of low back pain, current medications, past medical history.

(3) **Physical examination and vital signs**

- Medical interview and physical examination of general condition, nutritional status, skin/mucosa, eyes, ear-nose-throat, thyroid, lungs, heart/circulatory system, abdomen, kidneys/urogenital system, nervous/psychiatric system, spine/limbs/tumors, peripheral circulation, and lymphatic system.
- Vital signs: blood pressure (sitting), pulse rate, body temperature.
  Blood pressure and pulse are measured **after at least 5 minutes in the sitting position without abrupt postural changes**.

(4) **Imaging examination and assessment of Chuna malposition**

① Lumbar spine AP and lateral standing radiographs (L-spine AP & lateral standing view) will be obtained according to the Chiropractic Biophysics standardized

procedures described in **Section 6-1, Radiograph-based Chuna diagnostic method used in the clinical trial**.

② Based on the imaging DICOM files, the presence and type of Chuna spinal malposition will be determined.

(5) **Confirmation of inclusion/exclusion criteria**

- Final judgement of inclusion and exclusion criteria will be made by reviewing NRS scores, existing medical history, and prior treatment history.

---

2) Baseline visit (Visit 2, Day 0)
(Visit 1 and Visit 2 may be performed on the same day.)

For patients who pass the screening test and are enrolled in the clinical trial, **block randomization** will be performed, a subject number will be assigned, and the following assessments will be carried out:

(1) **Questionnaires on subjective symptoms, functional limitation, and quality of life**

- Numeric Rating Scale (NRS) – assessed separately for low back pain and leg pain.
- Oswestry Disability Index (ODI).
- European Quality of Life 5 Dimension (EQ-5D)-5L.
- Roland–Morris Disability Questionnaire (RMDQ).

(2) **Physical examination**

- Lumbar range of motion (ROM):
  Flexion, extension, lateral flexion (right and left), rotation (right and left).

(3) **Vital signs**

- Sitting blood pressure, pulse rate, and body temperature are measured **after at least 5 minutes in the sitting position without abrupt postural changes**.

(4) **Imaging examination**

- No separate imaging assessment is performed at Visit 2; the imaging evaluation at Visit 2 is replaced by the screening radiographs obtained at Visit 1.

---

3) Treatment visits (Visits 3–10; both groups)

(1) **Chuna + usual care group (Group I; Visits 3–10)**

- Starting immediately after the baseline visit, participants receive treatment **twice per week for 4 weeks**, for a total of **8 sessions** according to the allocated intervention (usual care + Chuna).

(2) **Usual care-only group (Group II; Visits 3–10)**

- Starting immediately after the baseline visit, participants receive **usual care only** twice per week for 4 weeks (8 sessions).

(3) **Assessments at each treatment visit (both groups)**

- Before treatment at each visit, the following items are checked:
  · Occurrence of adverse events since the previous visit.
  · Vital signs: sitting blood pressure, pulse rate, body temperature (measured after at least 5 minutes in the sitting position without abrupt postural changes).

---

4) **Assessment visits** (same for Groups I and II – Visits 2, 10, and 11)

(1) All participants who pass screening will visit the outpatient clinic at baseline, at the end of the treatment period (4 weeks after baseline), and 8 weeks after completion of all treatments (12 weeks after baseline) to undergo the following assessments.

(2) **Vital signs**

(3) **Assessment of subjective symptoms and function**

- Primary and secondary efficacy outcomes are assessed using questionnaires on pain intensity and function:
  NRS, ODI, EQ-5D-5L, and RMDQ.

(4) **Physical examination**

- Lumbar range of motion (ROM):
  Flexion, extension, lateral flexion (right and left), rotation (right and left).

(5) **Imaging evaluation**

- Standardized lumbar spine AP and lateral standing radiographs (L-spine AP & lateral standing view) are obtained.
- Based on the acquired images, vertebral-level relative angles (flexion, extension, lateral bending, rotation) and Chuna malposition diagnosis results are derived using a DICOM labeling program and compared across time points.

(Note: No separate imaging assessment is performed at Visit 2; the imaging evaluation at Visit 2 is replaced by the screening radiographs obtained at Visit 1.)

(6) **Exploratory efficacy assessment**

- From the end of the treatment period until the follow-up visit, the following items are investigated:
  whether additional invasive medical procedures related to low back pain were performed [e.g., injection therapy such as nerve block, radiofrequency ablation], whether strong opioid analgesics were used, and whether lumbar surgery was performed.

(7) **Monitoring of adverse events**

- In addition to these assessment visits, adverse events are checked frequently during the treatment period through interviews or questioning whenever necessary.

---

5) Early Discontinuation Visit

Participants who have received **at least one session** of the study intervention and then meet the criteria for early termination of the clinical trial will attend an **unscheduled visit 8 weeks after the last treatment session** to undergo an assessment visit. In principle, this assessment should be performed as an in-person visit; however, at the participant's request, items other than physical examination, vital signs, physical function tests, and imaging evaluation may be replaced by **telephone consultation** to the greatest extent possible.

If the clinical trial is terminated early or the participant drops out according to the discontinuation/withdrawal criteria, **no additional follow-up assessments** beyond this early termination visit will be conducted.

---

9. Criteria for discontinuation/withdrawal and early termination of the clinical trial

○ Criteria for discontinuation/withdrawal

1. The following medications, which are expected to affect the evaluation of the effectiveness of the interventions, **must not be taken from screening until completion of the clinical trial**.

① Strong opioid analgesics; local anesthetics and corticosteroid preparations administered separately from the trial interventions by the attending physician.

- However, based on the clinical judgement of the investigator, non-steroidal anti-inflammatory drugs and muscle relaxants that are prescribed as needed may be used (see **11. Rescue medication**).

② Any other drugs that, in the judgement of the clinical trial investigator, may pose a risk to the participant or introduce bias into the evaluation of the study results.

2. Cases in which the participant requests discontinuation of the trial intervention during the clinical trial, or withdraws consent to participate in the study.
3. Cases in which a serious adverse event occurs and the investigator judges that continuation of the trial is not possible.
4. Cases in which major protocol violations such as violation of inclusion/exclusion criteria are newly identified during the clinical trial.
5. Cases in which, although the early termination criteria are not met, the participant does **not attend at least 5 out of the planned 8 treatment sessions**.
6. Any other cases in which it is considered difficult for the participant to continue in the clinical trial, and the principal investigator/sub-investigator judges that the trial should be discontinued for that participant.

○ **Early termination of the clinical trial (for a participant)**

1. During the clinical trial, if symptoms improve and, based on both the participant's personal request and the judgement of the clinical trial physicians, it is determined that no further treatment is necessary, the clinical trial may be **terminated early** for that participant.
2. Participants whose clinical trial participation is terminated early for this reason are **not** included in the discontinuation/withdrawal group and are considered to have been **fully compliant with treatment**.

---

10. Treatment compliance (Treatment compliance)

The clinical trial investigator (or a delegated individual) will encourage and guide participants at every visit when usual care and Chuna manual therapy related to the clinical trial are provided, so that **treatment compliance is maintained**.

Treatment compliance is calculated based on the **number of planned treatment sessions** and the **actual number of sessions received**, using the ratio of the actual number of treatments to the planned number of treatments.

After randomization, treatment compliance is considered adequate when participants have received **at least 5 out of the 8 scheduled Korean medicine treatment sessions**.

To evaluate treatment compliance, the clinical trial investigator (or delegated individual) must keep a **record of the treatments provided**.

Participants whose clinical trial participation is terminated early due to **symptom improvement** according to the early termination criteria are regarded as having been **fully compliant with treatment**.

---

11. Rescue medication (Rescue Drug)

From the time of the screening visit, if **intolerable pain** occurs, the use of **certain limited medications** is permitted as rescue medication **at the discretion of the clinical trial investigator**, excluding opioid analgesics and anesthetics.

Specifically, the use of **limited non-steroidal anti-inflammatory drugs (NSAIDs)** and **muscle relaxants** is allowed (medications for protection of the gastrointestinal tract may be co-administered to reduce adverse effects of the rescue drugs).

If rescue medication is taken, the **date of administration and dose** must be recorded in the participant's **diary**.

▷ **Types of medications that may be used as rescue drugs**

- Skeletal muscle relaxants, anticonvulsants, anti-inflammatory analgesics, analgesics, drugs for neuralgia, anticonvulsants, antidepressants,
- Non-opioid analgesics, antipyretics, weak opioid analgesics, gastrointestinal medications (digestive agents).

---

12. Criteria for early termination of the clinical trial

If, during the conduct of the clinical trial, a **serious adverse event (SAE)** occurs that may have a serious impact on participant safety or on the progress of the trial:

- The investigator may terminate the participation of the affected participant in the clinical trial at that time.
- If the serious adverse event is considered to be **related to the trial intervention, all clinical trial procedures will be suspended**.

Whether the affected participant will continue to participate in the clinical trial after the adverse event will be decided through discussion between the participant and the

research team. If the research team determines, on medical grounds, that the participant can no longer safely continue in the trial, participation may be **forcibly terminated**.

---

13. Criteria for medical care and treatment of participants after completion of the clinical trial

If, during or after participation in the clinical trial, a participant experiences **worsening of symptoms related to non-acute low back pain** or **adverse effects** and requests related treatment, appropriate medical care will be provided in accordance with the hospital's standard guidelines.

If a participant requires treatment, all medical costs will be covered according to the terms of the **clinical trial insurance contract**.

Participants whose clinical trial participation has been completed may receive medical care at any time, under the direction of the attending physician, in preparation for the occurrence of unexpected **delayed adverse reactions**.

If an adverse event occurs during the clinical trial, the trial site will provide **appropriate medical management** until the participant has recovered, and the research team will decide, through discussion, whether the participant can continue to take part in the trial.

---

14. Efficacy and safety evaluation

14.1 Efficacy evaluation

◯ Primary efficacy outcome

- The **primary efficacy outcome** of this clinical trial is the **change in low back pain intensity** measured by the **Numeric Rating Scale (NRS)**.
- NRS for low back pain is assessed at **baseline (week 0), end of treatment (week 4), and follow-up (week 12)**, and the change from baseline at each time point is compared between the two groups.

◯ Secondary efficacy outcomes

① NRS for leg pain (radiating pain)

- Patients rate the intensity of radiating pain to the lower limb on an 11-point numeric rating scale (0–10), similarly to low back pain NRS.

- At baseline, week 4, and week 12, the following questionnaires are administered:

② Oswestry Disability Index (ODI)

- A standardized questionnaire that evaluates the degree of disability caused by low back pain in daily activities (such as personal care, lifting, walking, sitting, standing, sleeping, social life, and traveling).
- The total score is calculated according to the validated scoring method, and the degree of functional impairment is expressed as a percentage.

③ European Quality of Life 5 Dimension (EQ-5D-5L)

- A generic health-related quality-of-life instrument with 5 dimensions (mobility, self-care, usual activities, pain/discomfort, anxiety/depression), each with 5 levels of severity.
- In this trial, the EQ-5D-5L index score is calculated using the Korean valuation set and used as a secondary outcome.

④ Roland–Morris Disability Questionnaire (RMDQ)

- A questionnaire that assesses the impact of low back pain on daily activities and functional status.
- The total score is calculated from the number of items checked, and higher scores indicate greater disability.

2. Physical examination – Lumbar range of motion (ROM)

- To evaluate changes in lumbar mobility, lumbar ROM is measured at baseline, week 4, and week 12, including:
  · Flexion,
  · Extension,
  · Lateral flexion (right and left),
  · Rotation (right and left).
- Measurements are performed using a standardized method (e.g., inclinometer or goniometer) according to the study site's SOP, and the degrees are recorded.

3. Imaging evaluation

- At baseline (screening radiographs), week 4, and week 12, L-spine AP and lateral standing radiographs are obtained according to the standardized imaging protocol.
- Using a DICOM labeling program developed by the Korea Institute of Oriental Medicine, the following are calculated for each lumbar vertebral level:

· Relative angles for **flexion**, **extension**, **lateral bending**, and **rotation**,
· **Chuna malposition diagnosis results** based on the predefined cut-off values.

- These imaging parameters are used as secondary or exploratory outcomes to investigate changes in spinal alignment and their association with clinical improvement.

4. **Exploratory efficacy outcomes**

- From the end of the 4-week treatment period until the 12-week follow-up, the following are investigated and compared between groups as exploratory outcomes:

① Whether additional **invasive procedures** for low back pain were performed

  - e.g., injection therapy such as **nerve blocks**, **radiofrequency ablation**, or **lumbar surgery**.

② Whether **strong opioid analgesics** were used for pain control.

③ **Rate of early termination of treatment** due to sufficient symptom improvement (based on the early-termination criteria).

④ **Use of rescue medications**

  - Type, dose, and frequency of rescue medications (NSAIDs, muscle relaxants, etc.) are recorded and analyzed.

5. **Classification of responders and non-responders**

- Using the change in NRS from baseline, the **minimal clinically important difference (MCID)** for low back pain is defined as a **decrease of 2 points or more** on the NRS.

① Participants with a reduction of **≥ 2 points** on the NRS from baseline to each time point (weeks 4 and 12) are classified as the **treatment responder group**, and those with < 2-point reduction are classified as the **non-responder group**.

② In addition, participants whose trial participation is **terminated early due to symptom improvement** in accordance with the early-termination criteria are also classified as belonging to the **responder group**.

14.2 Safety evaluation

(1) **Physical examination and vital signs**: Performed at every visit after randomization

- The results of the physical examination and vital signs are reviewed comprehensively, and whether each test is normal or abnormal is determined for each individual participant.

(2) **Confirmation of adverse events such as subjective and objective symptoms**: Performed at every visit after randomization

- The clinical trial investigator must record all adverse events and concomitant medications that occur during the trial.
- Adverse events and concomitant medications are recorded in the case report form (CRF). For adverse events, the following must be documented: the symptoms and signs of the event, duration (start date/end date), severity, outcome, seriousness, causal relationship with the investigational intervention, and any actions taken in relation to the adverse event.
- Symptoms or signs that were already present before participation in the clinical trial are not recorded as adverse events. However, if after enrollment there is any change in the frequency, severity, or extent of such symptoms or signs, they must be recorded as adverse events.
- For concomitant medications, the generic name, dose, period of administration, and reason for use must be recorded in detail (for specific evaluation methods, see **Section 18. Methods for reporting and evaluating adverse events**).

---

15. Statistical analysis

The analysis sets used for the evaluation of data obtained in this clinical trial will be defined as follows:

- **Intention-to-treat (ITT) set**
  - The ITT set consists of **all participants who were randomized and received the trial intervention at least once**.
- **Full analysis (FA) set / Full analysis set (FAS)**
  - The FAS consists of all participants who:
    · were randomized, and
    · received the trial intervention at least once, and
    · have **at least one post-baseline efficacy assessment**.
  - The FAS follows the ITT principle and will be used as the **primary analysis set for efficacy**.
- **Per-protocol (PP) set**
  - The PP set includes participants who meet **all** of the following conditions:
    1. Fully satisfy the **inclusion and exclusion criteria**.

2. Have completed at least the **minimum predefined number of treatment sessions** (patients who received **5 or more** of the total 8 planned treatment sessions).
3. Patients whose treatment was completed because they **met the predefined criteria for treatment completion due to symptom improvement**.

- **Safety analysis (SA) set**
  - The safety analysis set consists of all participants who were randomized and received the trial intervention **at least once**, and for whom **at least one safety assessment** was performed.
  - The SA set will be used for **all safety analyses**.

In this clinical trial, the general principle for statistical analysis is as follows:

- All statistical tests will be **two-sided**, with a **significance level of α = 0.05**.
- Efficacy analyses will primarily be performed using the **full analysis set (FAS)**, with the **PP set** used as a **supplementary analysis**.
- Safety analyses will be performed in the **ITT (SA) set**, i.e., all randomized participants who received at least one treatment.

For missing data:

- For **continuous efficacy variables** in the FAS, missing values will be imputed using the **Last Observation Carried Forward (LOCF)** method, replacing missing values with the most recent available value prior to the missing time point.
- For all other variables, missing data will **not** be imputed, and analyses will be performed using the observed data as they are.

1) Analysis of demographic and baseline characteristics

- Demographic and baseline characteristics will be summarized using **descriptive statistics**.
- For **continuous variables**, depending on whether the normality assumption is satisfied, the number of observations, mean, and standard deviation, or the median with 25th and 75th percentiles will be presented.
- For **categorical variables**, frequencies and percentages will be presented.
- To compare continuous variables between groups:
  · If the normality assumption is satisfied, an **independent t-test** will be used.
  · If the normality assumption is not satisfied, a **Wilcoxon rank-sum test** will be used.
- To compare categorical variables between groups:
  · A **Chi-square test** will be used, or

· If more than 25% of cells have an expected frequency < 5, **Fisher's exact test** will be used.

---

2) Efficacy analysis

Efficacy analyses will be performed primarily in the **FAS**, with the **PP set** used for supplementary analyses. All statistical tests will be **two-sided** with a **significance level of 5%**, and **two-sided 95% confidence intervals** will be presented.

For each treatment group and each visit, descriptive statistics will be presented. For changes from baseline at each visit, descriptive statistics and 95% confidence intervals will be presented.

---

a. Analysis of the primary efficacy outcome

- For each measurement time point, changes in the **NRS score for low back pain** from baseline (week 0) to the end of treatment (week 4) and follow-up (week 12) will be summarized for each treatment group using descriptive statistics (number of participants, mean, standard deviation).
- For within-group tests of the change in NRS for low back pain (from baseline to week 4):
  · If the normality assumption is satisfied, a **paired t-test** will be used.
  · If the normality assumption is not satisfied, a **Wilcoxon signed-rank test** will be used.
- To evaluate the effect of the treatment group compared with the control group, an **ANCOVA model** will be used, with:
  · the **treatment group** as a fixed effect, and
  · the **baseline NRS score** as a covariate,
  analyzing the change in NRS from baseline to the end of treatment (week 4).
- The **least-squares mean (LSM) difference** between the treatment and control groups, the **two-sided 95% confidence interval** for this difference, and the **p-value** will be presented.

---

b. Analysis of secondary efficacy outcomes

Secondary efficacy outcomes will be analyzed using methods consistent with those for the primary efficacy outcome, focusing on changes from baseline (week 0) to the end of treatment (week 4) and follow-up (week 12).

- ① **Change in NRS for leg pain (radiating pain)**
  - The change in NRS for radiating leg pain from baseline to weeks 4 and 12 will be analyzed.
- ② **Evaluation of subjective pain intensity and quality of life related to low back pain**
  - Changes in the following outcomes from baseline to week 4 and week 12 will be analyzed:
    · ODI,
    · EQ-5D,
    · RMDQ.

For these continuous variables, within-group tests and between-group comparisons will be performed in the same manner as for the primary outcome (paired t-test or Wilcoxon signed-rank test for within-group changes; ANCOVA or appropriate nonparametric tests for between-group comparisons, as appropriate).

---

c. Exploratory efficacy analysis

Exploratory outcomes will be analyzed as follows:

- **Additional procedures/surgery after the study**

  ▶ Evaluation of additional nerve block procedures or surgery after the end of treatment

  - Within 8 weeks after the end of the treatment period, it will be investigated whether participants received additional procedures for pain control (e.g., **nerve block procedures**, radiofrequency ablation) or underwent **lumbar surgery**. Based on these data, the **rate of additional procedures** will be evaluated.
  - If significant differences between treatment groups are identified, **post-hoc tests** will be performed.
  - Between-group comparisons of the rates of additional procedures or surgery will be conducted using the **Chi-square test** or **Fisher's exact test**.
  - *Additional procedure rate* =
    (Number of patients who underwent additional procedures or surgery / Number of patients who completed the study) × 100
- **Rate of early termination of treatment**

  ▶ Evaluation of the early termination rate

- Among participants whose treatment was completed early because they met the predefined criteria for **treatment completion prior to the scheduled end of therapy**, the **rate of early termination** will be evaluated for each group.
- Between-group comparisons of the early termination rate will be conducted using the **Chi-square test** or **Fisher's exact test**.
- *Early termination rate* =
(Number of patients whose trial participation was terminated early / Number of patients who completed the study) × 100

- **Analysis of rescue medication use**

▶ Analysis of rescue medication use

- For each participant, information on the **type of rescue medication used**, **dose**, and **timing of administration** will be collected.
- For each treatment group, the number of uses and total amount of rescue medication by drug type will be compared between groups using an **independent t-test**, or, if the normality assumption is not satisfied, a **Wilcoxon rank-sum test**.

---

d. Classification of responders and non-responders and post-hoc analysis

- **Responder definition**

▶ Classification of treatment responders and non-responders

- Using the change in NRS for low back pain from baseline to each evaluation time point, the **minimal clinically important difference (MCID)** is defined as a **decrease of 2 points** on the NRS.
- Participants who show a decrease of **2 points or more** on the NRS are defined as having achieved MCID and are classified as the **treatment responder group**.
- Participants with less than a 2-point decrease are classified as the **non-responder group**.
- In addition, participants whose trial participation is **terminated early due to symptom improvement** because they meet the early termination criteria are also included in the **responder group**.

- **Post-hoc analysis**

▶ Post-hoc analysis based on responder classification

- Using the above definition of responders and non-responders, appropriate **post-hoc analyses** will be performed by a professional

statistician to investigate differences in outcomes between groups according to response status.

---

3) Safety analysis

- For adverse events, the **number of events**, **number of affected participants**, **severity**, and **causal relationship with the intervention** will be summarized descriptively by treatment group. If necessary, **nonparametric methods** may be applied.
- Vital signs, physical examination findings, and patients' subjective complaints will be comprehensively reviewed. For any parameters judged by the investigator to show **clinically meaningful changes**, statistical analyses may be performed as needed.
- For continuous data, the **number of observations**, **mean**, **standard deviation**, **median**, **minimum**, and **maximum** will be presented by visit. Within-group changes will be analyzed using a **paired t-test** or **Wilcoxon signed-rank test**.
- For adverse event data, between-group comparisons will be conducted using the **Chi-square test** or **Fisher's exact test**.
- If clinically meaningful changes are observed and a comparison between groups is needed, methods such as **generalized estimating equations (GEE)** may be used to evaluate whether there are statistically significant differences between groups over time.

---

16. Methods for reporting and evaluating adverse events

Following Chuna manual therapy, the expected adverse reactions include **worsening of pain at the treatment site**, **fatigue**, etc., and in rare cases **nerve injury** and **fracture** may occur. For ICT, possible adverse reactions include **skin irritation** at the treatment site, **burns**, **blistering**, and **discomfort after treatment**.

In this study, the scope of adverse events related to the study procedures is defined on this basis as follows:

- For **Chuna manual therapy**, adverse events include **worsening of pain**, **fatigue**, **fracture**, and **nerve injury**.
- For the **usual care group (ICT)**, adverse events include **burns**, **blistering**, and **worsening of pain**.

Adverse events that are clearly **unrelated to the study interventions** are **not** classified as serious adverse events for the purpose of this protocol. The methods for reporting and evaluating adverse events are described below and are adapted from the

standard procedures used in drug-intervention clinical trials, applying the same basic definitions and evaluation criteria.

The investigator must record **all adverse events** that occur during the clinical trial. In the case report form (CRF), the following must be documented for each adverse event:

- Symptoms and signs,
- Duration (start date / end date),
- Severity,
- Course and outcome,
- Seriousness,
- Causal relationship to the clinical trial procedure,
- Actions taken in relation to the adverse event.

---

1) Definitions

(1) **Adverse Event (AE)**
An adverse event is any unfavorable and unintended sign, symptom, or disease that occurs in a participant who has received a clinical trial procedure, and **does not necessarily have a causal relationship** with that procedure.

(2) **Adverse Treatment Reaction (ATR)**
Originally, an adverse drug reaction is defined as any noxious and unintended response to an investigational medicinal product at any dose, for which a causal relationship with the drug cannot be excluded.

Since this study is not a drug administration study but a **procedure-based study**, we apply this concept by defining an **adverse treatment reaction** as any adverse event for which there is a reasonable possibility of a causal relationship with the clinical trial procedure – that is, cases in which a relationship **cannot be completely ruled out**.

(3) **Unexpected Adverse Treatment Reaction**
An unexpected adverse treatment reaction is an adverse reaction whose **nature or severity** is **inconsistent** with the available information on the procedure (for example, the investigator's brochure or the package insert of drugs used for nerve block, etc.).

(4) **Serious Adverse Event (Serious AE)**
A serious adverse event is an adverse event occurring in association with the clinical trial procedure that meets **any** of the following criteria:

- Death of the participant during the trial period.

- A life-threatening event (the participant is in immediate danger of death at the time of the event; this does not refer to hypothetical situations where the participant might have died if the event had been more severe).
- An event that requires hospitalization or prolongation of existing hospitalization (however, hospitalization for simple rest or convalescence does **not** constitute a serious adverse event).
- An event that results in persistent or significant disability or substantial disruption of the ability to conduct normal life functions.

Even if a situation does not fall strictly into one of the above categories, if it is judged that the situation has a **significant impact on the participant's comfort or health status**, the responsible physician and relevant experts will determine, based on medical judgement, whether the event should be considered a **serious adverse event**, and appropriate measures will be taken accordingly.

(5) **Serious and Unexpected Adverse Treatment Reaction**
A serious and unexpected adverse treatment reaction is an adverse reaction whose nature or severity is inconsistent with the available information on the procedure, and that also meets the criteria for a **serious** event. When such a reaction occurs, **rapid reporting to the sponsor** is required.

---

2) Evaluation of adverse events

- In this clinical trial, adverse events include any undesirable and unintended signs or symptoms that were **not present before the start of the trial** and that newly appear during the treatment period, regardless of whether a causal relationship to the clinical trial procedure is established.
- All events that are expected adverse reactions to the clinical trial procedure (symptoms and signs, onset date, duration, etc.) must be fully and accurately recorded in the adverse event section of the CRF.
- The **severity** of adverse events will be graded by the principal investigator and sub-investigators according to a stepwise scale, with reference to **"4) Assessment of the relationship to the clinical trial procedure."**
- The **causal relationship** between the clinical trial procedure and the adverse event will be classified into **six categories** as described in **"4) Assessment of the relationship to the clinical trial procedure."**

---

3) Recording of adverse events

The **severity** of adverse events will be recorded by the principal investigator and sub-investigators according to the following criteria:

- **Grade 1 (Mild AE)** – Awareness of signs or symptoms, but easily tolerated.
- **Grade 2 (Moderate AE)** – Discomfort that interferes with normal daily activities.
- **Grade 3 (Severe AE)** – Inability to perform normal daily activities.

All adverse events occurring during the trial period must be recorded by the principal investigator and clinical trial staff.

Items to be recorded in the CRF for each adverse event

1. Symptoms and signs of the adverse event.
2. Onset date of the adverse event.
3. End date of the adverse event.
4. Severity of the adverse event.
5. Outcome of the adverse event.
6. Causal relationship between the adverse event and the clinical trial procedure.
7. Actions taken in response to the adverse event.

---

4) Assessment of the relationship to the clinical trial procedure

When an adverse event occurs, the clinical trial investigator will classify the relationship between the event and the **clinical trial procedure** according to the following categories, and record any additional opinion if necessary. The degree of relatedness is judged according to criteria based on the timing of onset, known characteristics of the procedure, and the exclusion of other causes. A typical 6-level classification is used:

① Certain

- A reasonable temporal relationship between the procedure and the event,
- The event cannot be explained by concurrent disease or other drugs/chemicals,
- The event improves upon discontinuation of, or reduction in, the procedure (dechallenge), and recurs upon re-administration (rechallenge), when applicable.

② Probable / Likely

- A reasonable temporal relationship,
- Unlikely to be attributed to concurrent disease or other drugs,
- Reasonable response to withdrawal of the procedure,
- Rechallenge information may be lacking.

③ Possible

- A reasonable temporal relationship,
- May also be explained by concurrent disease or other drugs,
- Information on response to withdrawal may be lacking or unclear.

④ Unlikely

- A temporal relationship that makes a causal relationship improbable,
- A plausible alternative explanation (e.g., underlying disease or other treatment) exists.

⑤ Conditional/unclassified

- Additional information is required for an appropriate assessment, or further data are currently under review.

⑥ Unassessable / Unclassifiable

- Information is insufficient or contradictory to make a judgement, and cannot be supplemented or verified.

---

5) Actions taken in relation to the adverse event

The actions taken with regard to the clinical trial procedure in response to an adverse event are classified as:

1 = Treatment withdrawn (procedure discontinued)
2 = Treatment reduced
3 = Treatment increased
4 = Treatment not changed (existing procedure maintained)
5 = Concomitant medication taken (additional medication given for treatment)
6 = Non-drug therapy given
7 = Unknown
8 = Not applicable

---

6) Outcome of the adverse event

The outcome of each adverse event is classified as:

1 = Recovered / Resolved
2 = Recovering / Resolving
3 = Not recovered / Not resolved
4 = Recovered / Resolved with sequelae

5 = **Fatal**
6 = **Unknown**

---

7) Reporting of adverse events

If a **serious adverse event** or serious adverse treatment reaction occurs during the clinical trial, the principal investigator and sub-investigators must ensure the **safety of the participant** and take prompt and appropriate measures to minimize the adverse event.

- The clinical trial investigator must **immediately report** the event to the principal investigator, who must then report it to the IRB within the timelines specified in the IRB's SOP at each institution.
- The principal investigator and sub-investigators must educate participants (or their guardians) about all potential adverse reactions that may occur after the clinical trial procedures, and instruct them to report all events occurring after the procedures.
- All systemic or laboratory findings that occur after the procedure (type of symptom, time of onset, severity, treatment, medications used, course of the event, causal relationship to the procedure, etc.) must be recorded and stored in the CRF in accordance with Good Clinical Practice.

The principal investigator will describe and evaluate all adverse events that occurred during the clinical trial in the **clinical study report**, and will report serious adverse events to the sponsor and IRB in accordance with applicable regulations.

Serious and **unexpected** adverse treatment reactions must also be reported to the IRB. Additional safety information should be reported periodically until the event is considered resolved (disappearance of the reaction or inability to continue follow-up). Even after completion of the trial, adverse events that occur **within 7 days after the clinical trial procedure** must be reported in accordance with these reporting principles.

The principal investigator will conduct the clinical trial in compliance with all aspects of the **Declaration of Helsinki**.

---

8) Follow-up of adverse events

The principal investigator or sub-investigators must follow up participants who experience an adverse event **until**:

- Symptoms improve, and

- Any abnormal laboratory findings return to baseline or to an acceptable level, **or**
- A satisfactory explanation has been obtained for the observed changes.

If required by the IRB, a **follow-up report** must be submitted to the IRB.

Measures to be taken when adverse events occur

During the entire clinical trial, the principal investigator and sub-investigators must give utmost attention to participant safety. In the event of a **serious adverse treatment reaction**:

- The participant's participation in the trial must be **discontinued**, and
- Prompt and appropriate measures must be taken to minimize the adverse event.

When a serious adverse treatment reaction occurs, the responsibilities of each party are as follows:

(1) **Responsibilities of the principal investigator**

- If a serious adverse event occurs during the clinical trial, the principal investigator must report it to the **sponsor within 24 hours**, and report it to the **Institutional Review Board (IRB)** without delay, in accordance with applicable timelines.

(2) **Responsibilities of the clinical trial investigator**

- If a serious adverse event occurs during the conduct of the clinical trial, the investigator must report it to the **principal investigator** and to the **IRB** within the timelines specified in the IRB's SOP.

(3) **Responsibilities of the Institutional Review Board (IRB)**

- The IRB reviews serious adverse events that occur during the clinical trial, assesses their impact on participant safety and on the conduct of the study, and may require modifications to the protocol, additional safety measures, suspension, or termination of the trial as necessary.

(4) **Responsibilities of the principal investigator in reporting serious and unexpected adverse treatment reactions**
When the principal investigator receives from an investigator a report of a **serious and unexpected adverse treatment reaction**, the principal investigator must attach a copy of that report to the adverse event report and, in cases that result in death or are life-threatening, submit the report **within 7 days** from the date on which the principal

investigator was first informed of, or became aware of, the event. In such cases, detailed follow-up information must be submitted **within 8 days** of the initial report.

The principal investigator must also periodically submit **additional safety information** until the adverse reaction has been concluded (i.e., resolution of the adverse reaction or inability to continue follow-up).

When an adverse event occurs, participants must be managed so that they can promptly receive any necessary examinations and treatment from the principal investigator or clinical trial investigators. In the event of a **serious adverse treatment reaction**, the principal investigator or clinical trial investigator may discontinue the trial for the affected participant and must take prompt and appropriate action in accordance with the procedures described under "Measures to be taken when adverse events occur."

---

17. Data management

Data management for this clinical trial will be conducted in accordance with **ICH-GCP** and **KGCP** regulations.

1. **Recording in the case report form and source document verification**
   All required data must be recorded in the **source documents** at the time they are generated. If any data have not been recorded by the end of the clinical trial, the reason for the omission must be documented.

All corrections to the source documents must be made by drawing a single line through the original entry so that it remains legible, and then recording the corrected data, the person making the correction, the reason for the correction, and the date of correction. Correction fluid or any method that obscures the original record must **not** be used.

Once the source documents for a participant have been completed, the corresponding data are entered into the **case report form (CRF)**. All source documents must be retained so that they can be verified upon request by the relevant governmental authorities, the IRB, and others.

Before starting the clinical trial, the investigator should indicate **normal ranges or reference values** in the CRF or other appropriate documents so that these can be used for verification and validation of the data.

2. **Completion of the case report form**
   Persons delegated by the principal investigator to complete the case report

forms must enter into the CRFs the contents of the source documents **accurately and faithfully**.

---

18. Informed consent form

Before conducting the clinical trial, the nature and scope of the study, as well as the expected effects and possible adverse reactions of the investigational procedures, must be **fully explained in advance** to the participant.

Only after the participant has understood this explanation and has agreed to participate shall the participant **sign the informed consent form**. The date (year, month, day) on which consent was obtained must be recorded in the CRF.
(See the **Participant Informed Consent Form** template.)

---

19. Regulations for compensation of trial-related injury

If an unexpected accident or injury occurs to a participant taking part in this clinical trial, appropriate compensation will be provided in accordance with the **Regulations on Compensation for Trial-related Injury** (see **Appendix 2. Regulations for Compensation of Trial-related Injury**).

---

20. Measures for protection of participant safety

1. Through screening examinations, participants will be **strictly evaluated** to determine whether they are appropriate for inclusion in this clinical trial.
2. The clinical trial will be conducted in accordance with the **approved protocol**, and during the study period, regular examinations and check-ups will be performed to evaluate the occurrence and severity of adverse events and adverse treatment reactions, and to take appropriate action when necessary.
3. To prevent infection during blood sampling, blood collection will be performed in a room where entry by the general public is restricted, and **only fully sterilized, single-use devices** will be used.
4. In emergency situations, measures will be taken in accordance with the **emergency response procedures** established at each trial site.

---

21. Other matters necessary to conduct the clinical trial safely and scientifically

1. Good Clinical Practice (KGCP)
   This clinical trial will be conducted in compliance with KGCP and the fundamental principles of the Declaration of Helsinki, so that the study is carried out with full ethical and scientific consideration.
2. Compliance with and amendments to the clinical trial protocol
   This clinical trial will be conducted in accordance with the protocol (including the participant information sheet and informed consent form) approved by the IRB.

All amendments to the protocol must be discussed between the sponsor and the principal investigator. Except in cases where immediate changes are required to eliminate an imminent hazard to participants, any changes to the protocol must receive prior approval from the IRB before being implemented.

If, in order to prevent immediate harm to participants, it becomes necessary to implement changes to the protocol before obtaining IRB approval, such changes must be reported to the IRB as soon as possible.

3. Participant consent
   For volunteers recruited through public announcements, the clinical trial investigator will explain, in a manner that is easy to understand, the nature and scope of the clinical trial and the expected outcomes, and will faithfully answer any questions from the volunteers.

After sufficient time for questions and answers has been provided, the participant will be asked to sign the prepared informed consent form, and the investigator who has provided the explanation and obtained consent will also sign the form.
(See Appendix 1. Participant Recruitment Announcement and the Participant Informed Consent Form.)

4. Confidentiality
   All personal information of participants must be anonymized using initials, codes, or similar methods. All investigators involved in the clinical trial must maintain confidentiality regarding the results obtained from the trial.

The principal investigator must securely store the signed informed consent forms and prepare a list linking participant numbers with participant names and any information necessary to identify the participants, so that the records can be retrieved at a later date.

5. Clinical trial monitoring
   To ensure that the clinical trial is conducted in accordance with the protocol and KGCP, monitoring will be carried out at the trial sites through the Korean Medicine Clinical Trial Center of Kyung Hee University Korean Medicine Hospital.

During monitoring, it must be confirmed that the entries in the CRFs are **complete and clear**, and that they are consistent with the source documents.
